# Advances in Chitosan-Based Nanoparticles for Drug Delivery

**DOI:** 10.3390/ijms22179652

**Published:** 2021-09-06

**Authors:** Veronika Mikušová, Peter Mikuš

**Affiliations:** 1 Department of Galenic Pharmacy, Faculty of Pharmacy, Comenius University in Bratislava, Odbojárov 10, 832 32 Bratislava, Slovakia; mikusova@fpharm.uniba.sk; 2 Department of Pharmaceutical Analysis and Nuclear Pharmacy, Faculty of Pharmacy, Comenius University in Bratislava, Odbojárov 10, 832 32 Bratislava, Slovakia; 3 Toxicological and Antidoping Center, Faculty of Pharmacy, Comenius University in Bratislava, Odbojárov 10, 832 32 Bratislava, Slovakia

**Keywords:** chitosan, nanoparticles, drug delivery, new formulations, targeting, controlled release, derivatives, nanocomposites, preparation schemes

## Abstract

Nanoparticles (NPs) have an outstanding position in pharmaceutical, biological, and medical disciplines. Polymeric NPs based on chitosan (CS) can act as excellent drug carriers because of some intrinsic beneficial properties including biocompatibility, biodegradability, non-toxicity, bioactivity, easy preparation, and targeting specificity. Drug transport and release from CS-based particulate systems depend on the extent of cross-linking, morphology, size, and density of the particulate system, as well as physicochemical properties of the drug. All these aspects have to be considered when developing new CS-based NPs as potential drug delivery systems. This comprehensive review is summarizing and discussing recent advances in CS-based NPs being developed and examined for drug delivery. From this point of view, an enhancement of CS properties by its modification is presented. An enhancement in drug delivery by CS NPs is discussed in detail focusing on (i) a brief summarization of basic characteristics of CS NPs, (ii) a categorization of preparation procedures used for CS NPs involving also recent improvements in production schemes of conventional as well as novel CS NPs, (iii) a categorization and evaluation of CS-based-nanocomposites involving their production schemes with organic polymers and inorganic material, and (iv) very recent implementations of CS NPs and nanocomposites in drug delivery.

## 1. Introduction

**Figure 1:**
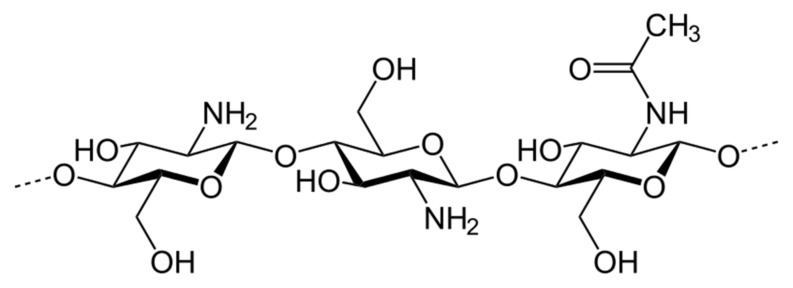
Chemical structure of chitosan.

Chitosan (CS) is one of the most exploited polymers in biomedical science, and it is the second most abundant next to cellulose, a naturally occurring amino polysaccharide [1]. It is produced from chitin (present in the exoskeleton of insects and marine aquatic animals, and microorganisms such as fungi, yeast, and microalgae) by partial deacetylation in an alkaline environment [2,3,4]. Structurally, chitosan is a linear polysaccharide composed of randomly distributed β-(1→4)-linked D-glucosamine (deacetylated unit) and N-acetyl-D-glucosamine (acetylated unit), see Figure 1. Depending on the source and preparation procedures the molecular weight of CS ranges from 300 to 1000 kD with 30–90% of deacetylation degree, DD [2]. The amino group in CS has a p*K*_a_ value of ca. 6.5, which leads to significant protonation in neutral solutions. These basic parameters (i.e., MW, DD, p*K*_a_) determine chitosan properties and action in biological systems.

Chitosan offers outstanding chemical and biological properties due to which it has gained enormous importance in various applications in pharmaceutical and biomedical areas, e.g., in drug delivery, gene delivery, tissue engineering, etc. The polycationic nature of CS makes it water-soluble and a bioadhesive that readily binds to negatively charged surfaces such as mucosal membranes. Thereby it increases the adhesion to the mucosa and as a result, enhances the time of contact for penetration of drug molecules through it [5,6]. Complexing properties of CS can be utilized in anionic drug delivery systems including low molecular drugs as well as polyanionic biomolecules such as DNA or siRNA [7,8]. Its carrier ability is increased with its charge so that it can serve as a pH-dependent drug carrier [1]. The mechanism responsible for the permeation enhancing effect of CS is also based on the positive charges of polymer, which seems to interact with the cell membrane resulting in a structural reorganization (opening) of tight junctions-associated proteins. Thus, CS of a high degree of deacetylation and high molecular mass exhibits the comparatively highest increase in epithelial permeability, and, by that, enhances the transport of polar drugs across epithelial surfaces [8]. It has excellent biocompatibility and low toxicity due to its chemical and structural similarity to the natural glycosaminoglycans. Chitosan is easily biodegraded into harmless products (amino sugars) that are absorbed completely in the body. Besides this, it is hemostatic, fungistatic, bacteriostatic, spermicidal, anticholesteric, and anticancerogenic [1,9,10,11,12,13,14,15,16]. It is worthy to mention also efflux pump inhibitory properties of CS. Here, CS inhibits certain transporter proteins on the membrane of intestinal epithelial cells or enterocytes, which pump out xenobiotics, mostly drugs, making these transporters one of the major components of drug resistance mechanisms [1].

CS properties and action in biological systems can be modified via proper physical and chemical modification of its chemical structure. Changes in its MW, DD, crosslinking, covalently bound functional groups and moieties, coordinated anions or polyanions, etc. can be advantageously utilized in various practical areas where CS is implemented. For example, introducing small chemical groups to the CS structure, such as alkyl (hydroxypropyl- or carboxymethyl groups can dramatically increase the solubility of CS [17]. Cationic property can be reversed via sulfonation to introduce an anionic character with water-soluble, better paste fluidity, high water reducing ratio, and anticoagulant property [18]. Mechanical strength of the particles increases in an increase in crosslinking of CS (e.g., with anions, dextran, sulfate, glyoxal, genipin, tripolyphosphate, formaldehyde, glutaraldehyde). Highly cross-linked particles show less swelling, less inside water penetration, and outside drug diffusion. Crosslinking can slow down drug release and prevent burst release [12]. Mucoadhesive properties can be improved by trimethylation of the primary amino groups of CS and PEG-ylation or immobilization of thiol groups on CS interacting with the cysteine-rich region of the mucous glycoprotein. Mucoadhesion can be improved also by the formation of complexes with multivalent anionic drugs, excipients, and multivalent inorganic anions [1,8]. A glutaraldehyde cross-linking of CS enhances an ability to quickly form hydrogel without the use of any acute harmful chemical [1]. In situ gelling properties of CS can be improved by thiolation [8]. Thiolation enhances the permeation effect even more up to 30 times in some cases; trimethyl chitosan (TMC) is soluble at all pH and it was also found to reduce the trans-epithelial electrical resistance by 25–85%. TMC is a great permeation enhancer as it was found to open tight junctions. It also increases the paracellular transport of the drugs [1]. Therefore, a great emphasis is laid on the development of various CS derivatives as has been documented by several recent review papers. Negm et al. [19] and Tian et al. [20] presented advancements on modification of CS biopolymer and potential applications of new CS derivatives. Mateescu et al. [21] reviewed the self-assembled systems based on CS derivatives for drug delivery. Other authors focused on the specific CS derivatives in their review papers. Among them, Fonseca-Santos et al. [22] discussed carboxymethylated derivatives of CS and their use as biomaterials and DDS. Dimassi et al. [23] discussed sulfonated and sulfated derivatives of CS for biomedical applications, and Tekade et al. [24] dealt with thiolated CS derivatives as the novel mucoadhesive polymers for better-targeted drug delivery.

Chitosan nanoparticles (CS NPs), reflecting properties of native or chemically modified CS polymers, can further and significantly spread the application possibilities of CS. The preparation of CS NPs is often conducted in mild conditions as CS is soluble in acidic aqueous solutions at room temperature, no toxic organic solvents or heat is required. A broad category of drugs can be incorporated into CS drug delivery systems (DDS), including small molecules, proteins, and polynucleotides. CS can release the encapsulated drug in a controlled fashion. The free amine groups on CS also provide ionic crosslinking ability [25]. Indeed, the general importance of “chitosan nanoparticles” is documented by ca. 1500 papers registered under this keyword in the WoS database in the first half of 2021. This review aims to summarize comprehensively knowledge coming from the recent review as well as research papers devoted to all aspects of CS NPs intended for drug delivery. Because of the plethora of relevant papers, proper classification, and organization of particular information in individual sections of this review is a prerequisite for an easy orientation in such extensive material. Based on this principle, we discussed all aspects related to the development of new CS NPs and nanocomposites (involving the most important CS modifications and production schemes of conventional as well as novel CS NPs and nanocomposites, employing various advanced organic as well as inorganic materials) and evaluated the developed systems in term of drug delivery enhancement. In this context, we evaluated very recent implementations of CS NPs and nanocomposites in oral, ocular, nasal, pulmonary, buccal, periodontal, vaginal, dermal, and transdermal drug delivery, and wound healing, but also vaccine and gene delivery.

## 2. Enhancement of Chitosan Properties by Its Modification

Although CS possesses many functional properties, it has several limitations such as high hydrophilicity, low ductility, a high degree of swelling and it is thermally less stable [12]. A major limiting factor in its utilization is poor solubility. CS is insoluble at physiological pH (pH 7.4) and ineffective as an absorption enhancer which interferes with its biomedical application. Improving the solubility of CS is a crucial factor for judicious use of the multitude of applications.

Chemical derivatives of CS can overcome limitations of unmodified CS and have received increasing interest over the past decade due to their advocated chemical, biological, and functional advantages over unmodified CS concerning their solubility, gelling properties, design of hydrophobic derivatives with amphiphilic character, and capacity to harness self-assembling nanostructures and chemical conjugates with an assortment of bioactive and therapeutic molecules. Other noteworthy benefits include improved biocompatibility and enhanced properties for complexing biomolecules (e.g., DNA, RNA). Varieties of CS derivatives have been developed throughout these years depending upon the requirements for various applications in biomedical areas [9]. The most used derivatives in the current pharmaceutical industry are made by quaternization, acylation, thiolation, and carboxymethylation [12].

Due to a variety of substituents of different nature, various derivatization procedures, and different activity and/or use of the products, it can be quite difficult to define criteria for specific groups of CS derivatives unambiguously. Although some aspects can overlap in particular groups, a specification of the basic categories should simplify orientation in produced CS derivatives. Negm et al. [19] divided CS modifications into the following categories: (i) substituted CS derivatives such as (I) thiolated CS, (II) phosphorylated CS, and (III) phtaloylated CS, (ii) cross-linked CS derivatives, such as (I) CS-glutaraldehyde cross-linked polymers, (II) EDTA CS polymer, and (III) CS-epichlorohydrin crosslinked polymers, (iii) carboxylic acid CS derivatives such as (I) CS carboxyalkylate derivatives, (II) CS methacrylate derivatives and (III) CS benzoylate derivatives, (iv) ionic CS derivatives such as (I) cationic CS derivatives and (II) sulphated CS derivatives and (v) bounded CS to specific molecules such as cyclodextrin linked CS. Bakshi et al. [13] presented the scheme highlighting (i) chemical modifications on -NH2 of glucosamine or on -OH groups of the polymer (such as (I) alkylation, acylation, hydroalkylation, carboxyalkylation, azidation, thiolation, (II) sugar derivatives, CS dendrimer hybrid, cyclodextrin linked, crown ether bonding, (III) metal ion chelates, grafting, cross-linking) and (ii) enzymatic modifications (using tyrosinase or chlorogenic acid). In their scheme following types of CS derivatives were included: hydroxyalkyl, acyl, N-alkylated, amphiphilic, thiolated, succinyl, cyclodextrin linked, quaternary ammonium derivative, enzymatic graft, crown ether bound, sialo dendrimer hybrid, PEGylated, galactosylated derivatives. In this review, we divided CS derivatives into six groups, according to the character of substituent/derivative, namely hydrophobic, amphiphilic, ionic derivatives, derivatives with specific substituents (sugar bound CS derivatives, CS derivatives with cyclic structures, CS derivatives with thiol groups, thiosemicarbazone linked CS derivatives), CS copolymers, and cross-linked CS derivatives. Wang et al. [26] summarized basic synthetic strategies and properties of CS derivatives in their review. They discussed particular CS structural modifications with respect to their biological activities and applications. In the following subsections, particular groups of derivatives, as they are listed in Table 1, are briefly discussed in terms of their modified properties, main functions, and utilization.

### 2.1. Hydrophobic Chitosan Derivatives (with Hydrophobic Substituents)

Alkylated CS. Generally, alkyl CS derivatives are obtained by reductive amination of CS. Short alkyl chain (C5) has almost no influence on the rigidity and the interaction properties of modified CS, whereas longer chains promote progressively more efficient hydrophobic interactions and intraaggregation of the polymer. However, the effect can be important if the basicity of the amino groups is altered. The hydrophobic character of alkylated CS increases with increasing chains length from C6 to C12. Moreover, the degree of substitution (DS) is an essential parameter in the formation of hydrophobic domains by alkyl-alkyl interactions [21,27]. Recently Burr et al. [28] used two methods to alkylate high MW CS with glycidyl trimethyl ammonium chloride (GTAC) to produce CS derivatives that are water-soluble throughout the pH range. In addition, a novel CS derivative was created by alkylating one of the products with the GTAC analog Quab 342 containing C12 alkyl chains. The study of phase behavior and rheological characteristics of the CS derivatives in the presence of anionic surfactant showed that the derivatives form soluble complexes at low and high SDS concentrations and that the Quab 342 derivative was able to form gels.

Acylated CS. Acylation is more versatile than alkylation as it allows (depending on reaction conditions) the introduction of hydrophobic moieties at the amino (N-acylation to form an amide), hydroxyl (O-acylation to form an ester), or both residues. The acylation of CS requires the use of organic acid derivatives (such as anhydrides, acid halides, etc.) as acylating agents in a proper reaction medium. The amino group of CS is more active than the hydroxyl group, so the acylation reaction generally occurs preferentially on the amino group. The amino group in CS could be protected before acylation, and then the protecting group could be removed to obtain only O-acylated CS [26]. The introduction of a hydrophobic moiety with an ester linkage increases its potential for biodegradability by lipase-like enzymes [13]. Acylated CS was proposed as an excipient for drug formulation, essentially because of their hydrophobic association that can enhance the stability of the matrices and can favor nanoparticle self-assembly [21]. N-acylated CS with saturated (C8–C18) and unsaturated (e.g., oleic, linoleic, elaidoic, and erucoyl) acyl groups of different chain lengths have been successfully synthesized. The degree of substitution was easily controlled by adjusting the number of fatty acids. After being grafted on the CS chain, the fatty chains increased the hydrophobicity and changed the structural features of the copolymer, which tended to self-assemble driven by hydrophobic forces [20]. Recently, Nanda et al. [29] developed an acylated CS (myristoyl and octanoyl) coated paclitaxel-loaded liposomal formulation to overcome the cremophor EL related toxicities. Slower drug release was observed in the case of liposomes coated with acylated CS as compared to uncoated and native CS-coated liposomes. All liposomal formulations were found less cytotoxic than paclitaxel injection (Celtax™, Celon Labs, India). In vitro cell uptake and intracellular distribution studies confirmed the cytosolic delivery of uncoated and coated liposomes. The myristoyl CS coated liposomal system exhibited better pharmacokinetic, biodistribution, and tumor uptake characteristics than other formulations. These results suggested the potential application of acylated CS coated liposomal delivery systems in tumor targeting.

N-phthaloylated CS. CS polymer is poorly soluble in organic solvents, while N-phthaloylation of CS is efficient for solubilization. N-phthaloyl CS derivatives have higher reactivity than N,O-phthaloylated CS derivatives. Completely deacetylated CS polymer reacted by phthalic anhydride yields N-phthaloyl CS derivative which has a high solubility in polar-organic solvents [19]. Permadi et al. [30] acylated a CS protected with phthalic anhydride (N-phtaloylated CS) and unprotected CS (without phthalic anhydride) via acylation on both primary hydroxyl and amine groups on the backbone of CS. This chemically modified CS was proposed to enhance the drug solubilization as well as improving biocompatibility and degradability.

Benzoylated CS. Benzoyl CS biopolymers play a significant role in drug delivery and cosmetics wound healing preparation, and chromatographic separation technologies [19]. Mohamed et al. [31] cross-linked chitosan with four various quantities of 4,4′-(5,5′ carbonylbis(1,3-dioxoisoindoline-5,2-diyl))dibenzoyl isothiocyanate named BBTU-CS-1,2,3,4. In comparison with the popular COX (cyclooxygenase) inhibitor Celecoxib, these hydrogels showed an inhibition activity towards COX enzymes with selective inhibition towards COX-2. BBTU-CS-4 hydrogel exhibited a potent inhibition against COX-2 (IC50 0.42 μg/mL) compared with that observed for the standard Celecoxib (IC50 0.26 μg/mL). BBTU-CS-4 was more potent against H. pylori compared to the other hydrogels. BBTU-CS-4 showed promising potential as anti-*H. pylori* and selective anti-inflammatory agent.

### 2.2. Amphiphilic Chitosan Derivatives (with Amphiphilic Substituents)

Cholic and deoxycholic acid-modified CS. Cholic and deoxycholic acids consist of hydrophilic moieties and a hydrophobic cyclopentanophenanthrene nucleus that enables them to form micelles in water because of their amphiphilicity. Modifying CS with cholic or deoxycholic acid confers to the polymer self-assembling properties, thus permitting its use as a potential carrier for hydrophobic drug molecules. The hydrophobic character of modified CS has an important role in the protection and the controlled release of the loaded drug [21]. Hanafy et al. [32] demonstrated CS and the counter-ion, sodium deoxycholate (SD), as a suitable system for the association with poorly-water-soluble drug molecules.

### 2.3. Ionic Chitosan Derivatives (with Cationic and Anionic Substituents)

Quarternary ammonium CS derivatives. The cationic nature of the CS is essential during several applications such as absorption enhancement, bio-adhesion, and transfection efficiency as well as to biological activities such as anti-tumor, and antihypercholesterolemic effect. Highly cationic derivatives of CS (including N,N,N-trimethyl CS chloride, TMC, as one of the most popular) were prepared by reaction of CS and an alkylhalogenide at an elevated temperature in a strongly alkaline environment to neutralize the acid being generated during the reaction and to avoid protonation of the unreacted primary amino groups [19]. The degree of quaternization (DQ) depends on the number of reaction steps, the reaction time, and the deacetylation levels of CS. The water solubility of TMC can be tailored according to DQ. With a suitable DQ, TMC can be dissolved in neutral and alkaline environments. Mucoadhesive properties are enhanced by increasing the cationic character of the CS due to the interaction with anionic groups of mucin. TMC has improved mucoadhesive property and absorption enhancement due to the stronger positive charge [33]. It has been shown that a 40–60% degree of quaternization has the best absorption-enhancing properties. Further increase in the DQ does not have a significant effect on absorption and shows increased toxicity. By quaternizing CS, thermosensitive hydrogels have been formed (e.g., for nasal drug delivery) [20]. Quarternization, the opposite of alkylation, increases the hardness of CS. Quarternized CS exhibit also better hydroxyl radical scavenging activity [13].

Sulfated CS derivatives. Sulfation of CS is preceded using different sulfating reagents, including concentrated H_2_SO_4_, oleum, SO_3_, SO_3_/pyridine, SO_3_/trimethyl amine, SO_3_/SO_2_, and chlorosulfonic acid-sulfuric acid [23]. CS sulfates have been shown to possess anticoagulant and hemagglutination inhibition activities due to the structural similarity to heparin apart from its antisclerotic, antiviral, anti-HIV, antibacterial, antioxidant, and enzyme inhibition activities. They also have high sorption capacities and are used in metal ion recovery. Sulfur compounds are grafted onto CS for recovery of mercury and the uptake of precious metals. Sulphonic CS is good flocculants for metallic oxides [13]. Ravindran et al. [34] prepared curdlan sulfate–chitosan nanoparticles were through the PEC method at a mass ratio of 2:1 respectively. A first-line tuberculosis drug, Rifampicin (Rif) and a phytochemical, DdPinitol (d-PIN), were encapsulated into Curdlan Sulphate (CuS)–chitosan nanoparticles (CuS/CS NPs) of size 205.41 ± 7.24 nm. Rif-CuS/CS NPs showed superior bactericidal activity (2.4–2.7-fold) within 4 h when compared to free drug Rifampicin (1.6-fold) and suppressed the pro-inflammatory gene (TNF-α by 3.66 ± 0.19-fold). d-PIN-RIF-CuS/CS NPs increased expression of the anti-inflammatory gene (IL-10 by 13.09 ± 0.47-fold) and also the expression of the TGF-β1 gene (13.00 ± 0.19-fold). Thus, CuS/CS NPs can be a potential nanocarrier matrix for the intracellular delivery of multiple drugs.

Succinylated CS. N-succinyl-CS is obtained by introducing succinyl groups on the N-terminal of the glucosamine units and the degree of substitution and the molecular weight can be easily controlled. With a high degree of substitution, it can be dissolved in alkaline but is insoluble in acid. It is an excellent absorption enhancer for intranasal drug delivery. As a drug carrier, N-succinyl-CS can readily prepare drug conjugates with various drugs due to-H_2_ and -COOH groups in its structure [20,35]. Palacio et al. [36] prepared succinyl-chitosan (SCS) nanoparticles by the ionic crosslinking method for polyphenols encapsulation and theoretically studied the interaction between SCS and polyphenols. Modification of CS with succinyl groups was carried out through the reaction between the –NH_2_ groups within the CS structure and succinic anhydride. Three polyphenols (epigallocatechin-3-gallate (EGCG), propyl gallate (PG), and gallic acid (GA)) with different molecular weights and number of –OH groups, were encapsulated. The encapsulation efficiency and loading capacity for each polyphenol were evaluated. GA showed an encapsulation efficiency of 88%, for EGCG it was 65%, and for PG was 27%. SCS-Polyphenol interaction energy behavior was studied through density functional theory (DFT) methods and a structure-binding relationship was found.

Phosphorylated CS. Phosphorylated CS was synthetized in various ways for example by using phosphorous pentaoxide in methane sulphonic acid as a solvent at low temperature, via reaction of CS and orthophosphoric acid at 150 °C in the presence of urea as a catalyst and DMF as a solvent, or by grafting copolymerization reaction of and mono-(2-methacryloyl oxyethyl) phosphoric acid. Phosphorylated CS and phosphorylated CS derivatives acquired their importance due to their characteristics such as high-water solubility, and metal chelating tendency. These CS derivatives had important applications in tissues regeneration and drug delivery [19]. Modification of CS with phosphorylcholine compounds imparts anticoagulant property [13]. Han et al. [37] prepared a poly(D,L-lactide) (PDLLA) membrane by the solution casting method, then the surface of the membrane was modified by polydopamine (PDOPA) as a substrate, followed by adsorption of different CS derivative: sulfonated chitosan (SCS) or/and phosphorylated chitosan (PCS) to obtain different functionalized membranes. In vitro, the cell culture results showed that the membrane functionalized by CS derivative could promote the proliferation of MC3T3-E1s and enhance the osteogenic differentiation by up-regulating the expression level of osteogenic genes compared to the PDLLA and P1/PDOPA membranes. Especially, when the outermost layer was SCS, the effect of promoting cell proliferation was better than that of PCS. However, for osteogenic differentiation, PCS had better quantitative experimental results than SCS. The results suggested that PCS and SCS have the potential value to be used as a functional modified material applied in bone tissue engineering.

Carboxyalkylated CS. The carboxylation of CS introduces acidic groups into the main chain of CS to improve the solubility, and film-forming properties of the product, and expand the application range of CS. The substitution order of carboxymethyl is C6-OH > C3-OH > C2-NH_2_. *O*-carboxymethylated CS reaction occurs in the presence of monochloroacetic acid and NaOH with isopropanol/water as a solvent at room temperature or in an ice bath. *N*-carboxymethylation and *N*,*O*-carboxymethylation mainly occur at elevated temperatures. *N*-carboxymethylation and *N*,*N*-carboxymethylation could be obtained by reacting CS with glyoxylic acid and using sodium cyanoborohydride reduction. In addition, *N*-carboxymethylated products could also be obtained by direct alkylation [26]. Carboxymethyl CS is one of the most significant water-soluble, amphoteric CS derivatives. It has great potential in medical applications as it is non-toxic, biocompatible, biodegradable, shows enhanced drug bioavailability, and has better-controlled release than original CS [12]. It is used in the development of different protein drug delivery systems such as super porous hydrogels, pH-sensitive hydrogels, and cross-linked hydrogels [13]. Carboxymethyl CS has attracted great interest in various fields such as antimicrobial activity, biosensors, wound healing, food industry, and bio-imaging [19]. Carboxymethyl CS plays a significant role in tissue engineering, it prevents postsurgical tissue adhesion formation [38]. Semisolid gels containing carboxymethyl CS are used as wound healing accelerators due to the enhancement of inflammatory cell and fibroblast function, which promotes tissue granulation and organization. N-CMCS is a more effective bacteriostatic agent than pure CS, the antimicrobial activity of O-CMCS depends on MW [22]. Carboxymethyl CS can serve as a precursor in photothermal responsive systems applicable in cancer therapy [39].

### 2.4. Sugar Bound Chitosan Derivatives

Galactosylated CS. Sugar bound CS have specific receptor binding properties and are being investigated for their antiviral properties. Galactosylated CS (GCS) is useful as hepatocyte targeting carriers and also as a synthetic extracellular matrix for hepatocyte attachment. GCS can be successfully implemented also in the treatment of cancer due to higher cytotoxicity, pH responsivity, and higher cell uptake [40].

Dendrimer hybrid CS. Dendrimer-like hyperbranched polymers, a new class of topological macromolecules. Various types of dendrimers have been used for drug delivery purposes, among them, polyamidoamine (PAMAM) dendrimers have been explored extensively for various biomedical applications including molecular imaging, drug delivery, and gene therapy. Sialo dendrimer hybrid CS were investigated as antivirals [35]. Recently, Sharma et al. [41] investigated the efficacy of dendrimer PAMAM/chitosan conjugate-based temozolomide (TMZ) nanoformulation (PAMAM/CS NPs) against gliomas in vitro as well as in vivo. The attractive ex vivo cytotoxicity against two glioma cell lines; U-251 and T-98G and phase solubility studies of TMZ revealed remarkable results. In vivo studies of prepared nanoformulation were significant and promising that explored the double concentration of TMZ in the brain due to surface functionality of dendrimer.

### 2.5. Chitosan Derivatives with Cyclic Structures

Cyclodextrin-linked CS. It combines the exceptional features of CS and cyclodextrin to form non-covalent host-guest complexes with several species of proper sterical dispositions [19]. CS bearing cyclodextrin pendants have been developed for improved applications such as drug delivery, cosmetics, and analytical chemistry [13]. Evangelista et al. [42] prepared supramolecular polyelectrolyte complexes (SPEC) based on a cyclodextrin-grafted CS derivative and carrageenan and evaluated them for controlled release of a model drug silver sulfadiazine. In vitro tests have shown a clear bacterial activity toward Gram-positive Bacteria *Staphylococcus aureus* and *Enterococcus durans/hirae* and Gram-negative bacteria *Klebsiella pneumoniae* and *Escherichia coli*. βCD/CS/carrageenan SPEC holds an expressive potential to be applied as a polymer-based system for controlled drug release.

Crown ether-linked CS. Crown ethers have good complexing selectivity for metal ions due to their specific molecular structures. Hence, crown ether-linked CS has a stronger complexing capacity and better selectivity for metal ions because of the synergistic effect of host-guest association and high molecular weight [13]. This crown ether-linked CS are investigated for adsorption for preconcentration of Ag+ for medical bacteriostasis. Yi et al. [43] synthesized a new di-Schiff base type crown ethers cross-linked chitosan (CCTS-1) by the reaction of 4,4′-di-formyl dibenzo-18-c-6 crown ether with cross-linked CS. New di-secondary amine type crown ethers cross-linked chitosan (CCTS-2) was prepared by the reaction between CCTS-1 and sodium borohydride. The adsorption rates by CCTS-2 for Ag+ for 1 h were 96% at pH 6.0, and Ag+ initial concentration 0.5 mmol/l. The complexes of CCTS-1, CCTS-2, and silver ion against three bacteria were studied. The bacteriostasis zone diameters of the complex of CCTS-2 and Ag+ (CCTS-2–Ag+) containing 0.00355 mmol Ag+ against Staphylococcus Aureus, Escherichia coli, and *Pseudomonas aeruginosa* are 11, 10, and 7.5 mm, respectively, while those of the complex of CCTS and Ag+ (CCTS–Ag+) under similar conditions were 11, 10, and 6.0 mm, respectively.

### 2.6. Chitosan Derivatives with Thiol Groups

Thiolated CS. Thiolated CS is synthesized by immobilization of thiol-bearing moieties in the 2-position of glucosamine. The thiolated CS derivatives include different compounds such as CS-cysteine, CS-thiolactic acid, CS-thioglycolic acid, CS-homocysteine, CS-4-thiobutylamidine, CS-thioethylamidine, CS-glutathione, CS-N-acetylcysteine, and CS-6-mercaptonicotinic acid conjugates [24]. The sulfur groups on thiolated CS can form disulfide bonding with cysteine-rich areas on mucin glycoprotein, therefore such derivatives have significantly improved mucoadhesion. Another mechanism of mucoadhesion of thiolated CS derivatives is in situ crosslinking property between the polymers. During the interpenetration process, disulfide bonds are formed within the thiolated CS, which results in anchors chaining up to the mucus gel layer. Due to their in situ gelling properties, a liquid thiolated CS cell suspension can be poured into a mold of the desired shape of the scaffold or can be directly injected at the site of tissue damage. This in situ gelling property is particularly suitable for liquid or semisolid vaginal, nasal, and ocular formulations. The thiolated CS compounds were promising as drug carriers and efficient as nano-medicine for enhancing ocular bioavailability. Thiolated CS enhances permeation by inhibiting protein tyrosine phosphatase, which is responsible for dephosphorylation or phosphorylation of tyrosine subunit occluding that leads to the closing or opening of the tight junctions. It displays excellent cohesive properties for prolonged controlled release of embedded therapeutic ingredients [13,19,20]. Guaresti et al. [44] synthesized nanometric gel particles through water in oil emulsion by the covalent cross-linking between the primary amines of thiolated chitosan (CS-SH) and the dicarboxylic poly (ethylene glycol) (PEG) derivative as cross-linker. The amidation reaction led to the formation of stable nanosized networks positively or negatively charged by the ionization of either amines or thiol moieties depending on the surrounding pH. The presence of thiol groups in the nanogels was demonstrated to greatly improve the nanoparticle–mucus interactions. Nanogels were further labeled with folate ligand as an example of a simple and easy functionalization route via thiol–Michael addition (MA) reaction for future studies of tumor-targeting therapies of these nanocarriers.

### 2.7. Thiosemicarbazone Linked Chitosan Derivatives

Thiosemicarbazone linked CS. Various modifications of thiosemicarbazone can be effectively utilized to tune the polarity and complexing properties of the resulting derivatives. Thiosemicarbazone derivatives of CS reported by Adhikari et al. [45] improved solubility and anticancer efficacy and novel thiosemicarbazone CS derivatives reported by Qin et al. [46] show antifungal efficacy. They obtained three novel derivatives via condensation reaction of thiosemicarbazide CS with phenyl aldehyde, o-hydroxyphenyl aldehyde, and p-methoxyphenyl aldehyde, respectively. The derivatives had a broad-spectrum antifungal activity that was greatly enhanced in comparison with CS. At 0.05 mg/mL, the o-hydroxyphenyl aldehyde thiosemicarbazone CS inhibited the growth of *R. solani* at 52.6% and was stronger than polyoxin whose antifungal index was found to be 31.5%.

### 2.8. Chitosan Copolymers (with Polymer Substituents)

PEGylated CS. PEGylation (poly(ethylene glycol) is a technique to increase the circulation half-life of the particles leading to prolonged therapeutic effects, dose reduction, and improvement in patient compliance. PEGylated CS is usually unsuitable for mucus administration due to steric hindrance, which prevents close contact between the particles and the mucus, resulting in poor absorption. On the other hand, it is suitable for gene delivery since it can protect the model drug for a prolonged time to reach the targeted location [20]. Hsu et al. [47] fabricated by self-assembly of the amphiphilic benzoic-imine-containing PEGylated CS/4-(dodecyloxy)benzaldehyde (DBA) conjugates in an aqueous solution of pH 7.4 to efficiently promote loading efficiency and aqueous photostability of indocyanine green (ICG). The resulting polymeric micelles were characterized to have a hydrophobic hybrid CS/DBA core surrounded by hydrophilic PEG shells. Importantly, the encapsulation of ICG not only promoted the ICG loading but also enhanced its aqueous photostability. The robust ICG-loaded polymeric micelles showed several superior properties including the inhibition of ICG leakage under the mimic physiological and acidic conditions, favorable biocompatibility, and photo-activated hyperthermia, and showed potential in cancer theranostic.

PEG-methacrylated CS. A CS grafted-poly(ethylene glycol) methacrylate copolymer is obtained via Michael addition. The double crosslinking (ionic and covalent) procedure is carried out in the reverse emulsion. This chemical modification of CS enhances its solubility in aqueous media. PEG-methacrylated CS can serve as a polymeric nanocarrier for drug delivery [48].

### 2.9. Crosslinked Chitosan Derivatives

The mechanical strength of the particles increases with crosslinking of CS. Highly crosslinked particles show less swelling, less inside water penetration, and outside drug diffusion. The effect of crosslinkers on drug release is to slow down drug release and avoiding burst release [12].

CS-glutaraldehyde cross-linked polymers. Macro- and micro-properties such as permeability, wetting, mechanical properties, and chemical resistance were improved by the crosslinking process of CS biopolymer with glutaraldehyde. Yu et al. [49] prepared a stimuli-responsive (temperature and/or pH) three-dimensional cross-linked hydrogel system containing carboxymethyl chitosan (CMC) and poloxamer composed of a poly (ethylene oxide)/poly (propylene oxide)/poly (ethylene oxide) (PEO–PPO–PEO) block copolymer. The hydrogels designed for ophthalmic drug delivery were synthesized via a cross-linking reaction using glutaraldehyde (GA) as the cross-linking agent. The result of rheological studies showed that the gelation temperature was 32–33 °C and the viscosity of the hydrogel increased quickly after gelation. The results of a CCK-8 (Cell Counting Kit-8) assay showed that the hydrogel and its physical mixture solution were not cytotoxic to human corneal epithelial cells at a low concentration. The release rate of the model drug nepafenac was found to be maximum at 35 °C and pH 7.4.

CS-ethylene diamine tetraacetic acid cross-linked polymers. Biosorbents, particularly crosslinked CS, are widely utilized during heavy metal uptake. Cross-linker type and degree of crosslinking are largely influence the metal uptake behavior of CS-derived biosorbents. Grafting of ethylene diamine tetraacetic acid (EDTA) on CS biopolymer enhances the antibacterial potency due to the chelating magnesium ions which stabilize the outer cellular membrane of gram-negative bacteria. CS-EDTA modified biopolymer utilized as carrier matrix in the process of controlled drug release. The controlling function of the drug’s release came from the ionic crosslinking of the biopolymer by dicationic species 1,8-diaminooctane or lysine [19]. The majority of papers with CS-EDTA were devoted to heavy metal uptake. For example, Zhuang et al. [50] reported a cost-effective and efficient adsorbent for both Co2+ and Sr2+ nuclides removal, whilst their adsorption behavior and mechanism were also explored. In a multi-component system, EDTA-CS was an efficient broad-spectrum adsorbent for many types of metal ions even at a low pH level (pH = 1.2). Furthermore, its adsorption affinity was affected by the nature of grafting EDTA fragments and the remaining CS fragments. Coordination and electrostatic interaction played a vital role in the adsorption process.

CS-tripolyphosphate polymers: CS NPs, produced by ionic gelation with TPP are among the most intensely studied nanosystems for drug delivery. However, a lack of inter-laboratory reproducibility and a poor physicochemical understanding of the process of particle formation have been slowing their potential market applications. Sreekumar et al. [51] found out that for a given CS to TPP molar ratio, the average hydrodynamic diameter of the particles formed is strongly dependent on the initial CS concentration. The degree of acetylation of the CS was found to be the second most important factor involved in the system’s ability to form particles. The viscosimetry studies indicated that the particle formation and the average hydrodynamic diameter of the particles formed were highly dependent on the presence or absence of salts in the medium. By controlling the initial concentration of the solution and its solvent environment, it is feasible to control in a reproducible manner the production and characteristics of CS particles ranging in size from nano- to micrometers.

## 3. Enhancement in Drug Delivery by Chitosan Nanoparticles

There are several advantages of CS-based drug delivery systems (DDSs), mentioned in Section 3.4., therefore this area of CS use is extensively studied. The applications of NPs in the pharmaceutical industry are increasing day by day. In recent years, CS has been proven as a highly potent material in designing nanoparticle-based formulations for drug delivery, as reported by many review papers. Ali et al. [18] discussed the different types of CS nanocomposites and their use in biomedical applications. Garg et al. [52] and [53] Agnihotri et al. described methods for the preparation of CS NPs and their use in drug delivery. In addition, El Shoueir et al. [54] dealt with preparation methods of CS NPs and in addition, with physicochemical properties of CS. Rizwan et al. [12] studied the effect of CS properties on drug release applications. The following subsections are focused on a brief view of basic characteristics of NPs (Section 3.1), the most important/frequently used procedures for the preparation of CS NPs (Section 3.2), types and properties of CS-based nanocomposites (Section 3.3), and recent applications of CS NPs in drug delivery (Section 3.4).

### 3.1. Basic Aspects of (Chitosan) Nanoparticle Systems

Generally, NPs can be defined, concerning pharmaceutical use, as submicron colloidal drug carrier systems which are composed of natural or artificial polymers usually ranging in size between 10 and 100 nm [52]. Size is a key factor in determining the absorption, distribution, target site accumulation, and elimination of NPs in the body [55]. Particle size can affect the internalization of NPs (within the endocytosis pathway), and thus may influence the transmembrane transport capacity and rate of NPs. The uptake of NPs by intestinal cells or M cells after oral administration is reliant on size, which can influence their oral absorption efficiency and speed. Small particles are absorbed by intestinal cells, whereas large particles are mainly absorbed by M cells [56,57]. For effective drug delivery to target sites (e.g., tumors) or excellent sustained release, NPs should avoid being captured by the RES or filtered by the lung, liver, and spleen, and excreted by the kidneys. In general, NPs larger than 10 nm are able to avoid renal clearance and penetrate tissues. Although 10–20 nm particles can be widely distributed in various organs through tight endothelial connections, they will also be rapidly excreted by glomeruli [58]. Smaller particles, having a more curved surface than larger ones, cannot usually obtain the optimal geometry of the activated complement system, and therefore they are unlikely to be absorbed by macrophages contributing to their long circulation time in the blood. Particles larger than 200 nm can be quickly engulfed by the mononuclear phagocytic system and cumulate in the liver and spleen [59]. Thus, it can be concluded that NPs with small sizes are beneficial for long circulation and distributions in different tissues (in most cases). However, it has to be realized that the particle size should not be as small as possible because too small particles will lead to increased instability of the nanosystem under physiological conditions and will not achieve the expected effect [57]. 

The shape is another important basic parameter of NPs affecting and determining their biological processes in vivo. Phagocytosis of NPs by macrophages is the first obstacle faced by NPs in blood circulation, thus phagocytosis of macrophages is strongly shape-dependent [59]. The shapes of NPs can affect both their adhesion and internalization [60]. In addition to affecting the blood circulation time, in certain cases, the particle shape can also affect its in vivo distribution. Non-spherical nanocarriers (rod, ellipse, cylinder, ring, star, and other complex shapes) may have a profound impact on the transmission of the vasculature and may then affect their distributions [60,61]. In summary, although most of the NPs that have currently been studied are spherical, the unique properties of non-spherical NPs may have a significant effect on circulation and distribution [55].

Surface hydrophobicity and charge also belong to basic parameters of NPs affecting their biological processes in vivo. Hydrophilic nanomaterials can reduce the RES (reticular endothelial system) effect of NPs, whereas hydrophobic ones can improve the penetration effects of NPs. Hence, it seems to be reasonable to combine the two types of materials in the design of NPs at a certain proportion to allow for the best residence in vivo and optimal targeting to the disease sites. The surface charge may affect the adsorption of some specific molecules (e.g., opsonins), thus causing nanocarriers to be recognized, phagocytosed, and eliminated by macrophages, which can then further affect their transport and fate in the body [62,63].

In the area of nanomedicine, the targeting effect of nanocarriers reflects in increasing the accumulation of particles at disease sites [64]. Compared with passive targeting (size, shape, surface charge, and hydrophobicity) of NPs, active targeting (modified by ligands) can more actively and effectively transfer NPs to the target site. Furthermore, the modification of targeting ligands can also reduce the clearance of NPs. Hence, modification of NPs with ligands based on the characteristics of the disease site can be considered as one of the major future research directions for nanocarriers targeting [55].

**Figure 2:**
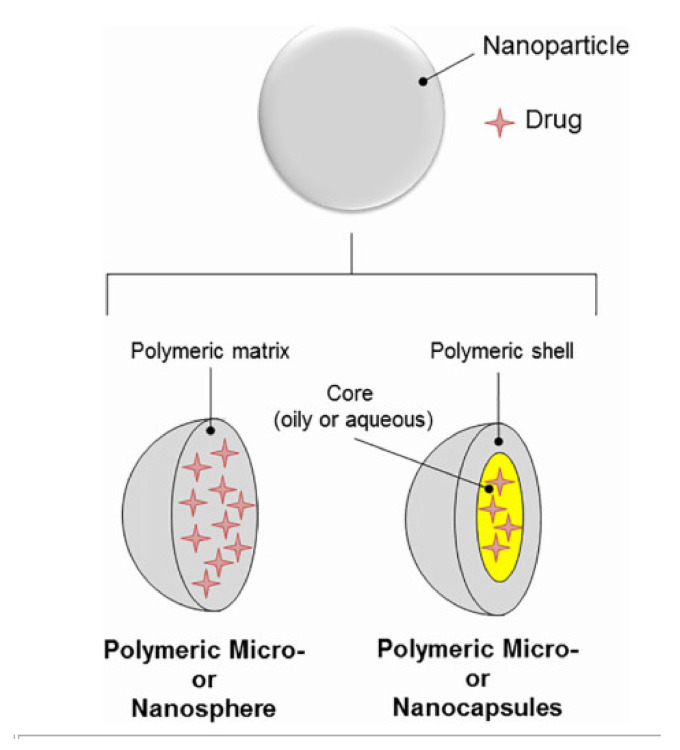
Schematic difference between polymeric micro- or nanocapsule and micro- or nanoparticle drug delivery systems [22]. Reproduced with permission from Elsevier (Copyright©2017).

NPs can be classified into two major groups based on their morphology: (i) nanospheres and (ii) nanocapsules. Nanospheres represent solid structures with a homogeneous matrix structure in which resources are distributed uniformly, while nanocapsules create a traditional hollow shell structure consisting of a polymer membrane and an internal core of the included drug [54]. The different structures of polymeric micro- or nanocapsule and micro- or nanoparticle drug delivery systems are illustrated in Figure 2. Both materials are the main target of the inclusion of active substances, have a high carrying capacity, and provide controlled release.

The advanced nano drugs are more suitable than the conventional medicines or microdrugs in terms of site-specific target capabilities, sustained and controlled release, increased absorption rate and bioavailability, and improved stability of therapeutic agents. The NP size, hydrophobicity, modified surface, high surface to volume ratio, and surface charge are the essential factors that control the targeting capabilities. These properties also help in reducing the required drug dose and frequency of administration leading to reduced toxicity and side effects of the chemotherapy drugs and thus improving patient compliance. Currently, polymers are being widely used for pharmaceutical applications ranging from general to highly complex DDS. The polymeric NPs are considered more advanced than simple NPs due to their capability towards prolonged release with improved drug safety and adjustable pharmacokinetics [12,52].

Drugs can be easily attached to nanocarriers either by adsorption or covalent linkage. Drug loading in nanoparticulate systems can be carried out by two methods, (i) during the preparation of particles (incorporation) and (ii) after the formation of particles (incubation). In these systems, a drug is physically embedded into the matrix or adsorbed onto the surface. The efficiency of loading largely depends upon the method of preparation of NPs as well as the physicochemical properties of the drug. Although maximum drug loading can be achieved by incorporating the drug during the formation of particles, it may get affected by the process parameters such as the method of preparation, presence of additives, etc. [53].

The drug release mechanism from the polymeric NPs is controlled by the degradation rate of polymer and diffusion of the drug from the polymeric matrix. In this regard, the diffusion of the drug is classified into four basic categories: (i), diffusion of the drug due to water intake, (ii), due to osmotic pressure, (iii) through the polymeric matrix, and (iv), due to erosion mechanism. In the first case, the pores in the polymeric matrix get wider with time due to their biodegradable nature. Water enters the polymeric matrix through these pores and widens them, which ultimately makes sufficient openings for the drug molecules to diffuse out of the polymeric matrix. In the second case, the drug release occurs through the water-filled pores and is based on osmotic pumping. In the third case, the drug diffusion is mainly based on the permeability and thickness of the polymer. In the fourth case, the drug release is based on the rate of degradation of the polymeric matrix. The polymeric material starts degrading from outside to inside and the drug release occurs when the erosion rate is higher than the water penetration rate. This kind of drug release has significance in terms of controllable and reproducible kinetics [12].

Polymeric nanoparticles based on chitosan (CS NPs) are acting as an excellent drug carrier because of some intrinsic beneficial properties of CS, such as biocompatibility, biodegradability, non-toxicity, bioactivity, easy preparation, and relative to some extent, target specific triggered by its cationic character [18]. The advantages and limitations of CS NPs are summarized in Table 2. CS NPs have a good mucoadhesive capacity, enhanced bioavailability, and dissolution rate of hydrophobic drugs. Besides, CS improves the stability of labile drugs in GIT, their bioavailability, and the controlled release of the drug, as the particles are beyond the nanoscale range to effectively overcome barriers and enhance permeability. However, CS NPs have also some disadvantages (disadvantages of CS itself, leading to the need for its modification, which was discussed in Section 2). Their low solubility at physiological pH affects the intake and causes a pre-systemic metabolism with the oral administration of drugs. The loading efficiency of CS alone to encapsulate water-insoluble drugs is generally quite low [54].

Both water-soluble and water-insoluble drugs can be loaded into CS NPs. Water-soluble drugs are mixed with CS solution to form a homogeneous mixture, and then, particles can be produced by any of the methods discussed in Section 3.2. Water-insoluble drugs and drugs that can precipitate in acidic pH solutions can be loaded after the formation of particles by soaking the preformed NPs with the saturated solution of the drug.

Drug release from CS-based particulate systems depends on the extent of cross-linking, morphology, size, and density of the particulate system, physicochemical properties of the drug as well as the presence of adjuvants. In vitro release also depends upon pH, polarity, and the presence of enzymes in the dissolution media. In most cases, drug release follows more than one type of mechanism (as discussed generally for NPs above in this section). In the case of a drug adsorbed on the surface of CS NPs and also in the case of a drug entrapped in the surface layer of NPs, a drug instantaneously dissolves when it is in contact with the release medium. This type of drug release leads to a burst effect in the early stages of dissolution. Drug release by diffusion is initially slow but later becomes fast [53].

CS NPs can assume various shapes. The most common are (i) nanospheres (NSs), (ii) nanocapsules (NCs), and (iii) nanofibers (NFs).

(i). CS NSs are a matrix system, where the drug may be absorbed in the surface or encapsulated within the CS particle. As an example, Liu et al. [39] constructed the photothermal sensitive carboxymethyl CS nanospheres (CMC NSs) carrier by introducing controllable heat-sensitive groups into CMC molecules. The carrier owned high drug loading and improved the stability of coated-drug DOX. The NSs generated photothermal response through NIR irradiation to improve the drug release amount and to achieve the combined treatment effect of photodynamic therapy and chemotherapy. In vitro photothermal tests proved that the NSs had excellent light stability and photothermal conversion performance. The cytotoxicity test results showed that the NSs had no obvious toxicity, but the drug-loaded nanospheres could effectively inhibit the growth of HepG-2 cells via photo-response to release DOX and Indocyanine green for achieving photothermal-chemotherapy under NIR irradiation.
(ii). CS NCs are vesicular systems in which the drug is generally confined to a cavity consisting of an oily core covered by a CS shell. As an example, Castro et al. [65] evaluated the physicochemical and biological properties of docetaxel (DCX) loaded chitosan nanocapsules (DCX-CS NCs) functionalized with the chimeric monoclonal antibody ChiTn mAb (highly specific antigen for carcinomas) (DCX-CS/PEG-ChiTn mAb NCs) as a potential improvement treatment for cancer therapy. The NCs, formed as a polymeric shell around an oily core, allowed a 99.9% encapsulation efficiency of DCX with a monodispersity particle size in the range of 200 nm and a high positive surface charge that provided substantial stability to the nanosystems. Uptake studies and viability assay conducted in A549 human lung cancer cell line in vitro demonstrated that ChiTn mAb enhanced NPs internalization and cell viability reduction.
(iii). CS NFs can be used in various fields mainly due to the presence of -NH2 and -OH groups, along with their specific structure. Their nanofibrous structure offers enormous possibilities for chemical modifications that create new properties applicable, particularly in the biomedical field. CS NFs can be prepared by electrospinning of CS into ultrafine fibers of nano size. Owing to the large specific surface area, NFs can deliver drugs, peptides, and vaccine antigens. The release of the drug may be immediate, delayed, or modified depending on the type of interactions between the polymer and the drug. Usually, an immediate release is noticed when a composition of a water-soluble substance and a water-soluble polymer is used. The prolonged release can be achieved by integrating the drug into other nanocarriers, such as NPs, liposomes, dendrimers, then loaded into NFs or use hydrophobic polymers [18,66]. As an example, Amiri et al. [67] reported the development of a local antibiotic delivery system using chitosan/polyethylene oxide (CS/PEO) NFs for delivery of teicoplanin. Uniform and bead-less NFs were prepared via electrospinning of a CS/PEO solution containing teicoplanin. The NFs were able to release teicoplanin for up to 12 days. Antibacterial test in agar diffusion and time-kill study on Staphylococcus aureus also demonstrated that loading teicoplanin in CS/PEO NFs enhanced the antibacterial activity up to 1.5- to 2-fold. An in vivo study on a rat full-thickness wound model confirmed the safety and efficacy of applying the teicoplanin-loaded NFs and significant improvement in wound closure were observed especially with the NFs containing 4% teicoplanin.

### 3.2. Preparation Procedures for Chitosan Nanoparticles

Processes of nanoparticulation can be divided into different categories: (i) physicochemical processes in which the NPs are precipitated by using preformed polymers, with yield induced by emulsification-solvent evaporation, diffusion, or reverse salting-out; (ii) by in situ chemical synthesis of macromolecules which lead to polymerizations or interfacial polycondensation reactions; and (iii) mechanical processes using high energy devices such as high-pressure homogenizers, ultrasonic devices, or wet high energy milling. Several methods have been reported for the preparation of CS NPs [18]. The main methods for producing CS NPs and nanocapsules (NCs) are ionic gelation, emulsification and crosslinking, complexation with polyelectrolytes, self-assembly, and drying processes [54]. The following subsections are describing the most important methods for the preparation of CS NPs, discussing also recent improvements in production schemes of conventional as well as novel CS NPs (such as optimized working parameters and conditions, new crosslinking agents, proper combinations of preparation schemes, etc.).

#### 3.2.1. Covalent Cross-Linking

Covalent cross-linking refers to the coupling of two or more molecules together via covalent chemical bonds (in ionic cross-linking it was physical binding through ionic interactions). In the case of CS NPs, covalent bonds are formed between CS or its derivatives and a functional cross-linking agent [52]. Crosslinkers usually possess a plurality of functional groups such as organic dibasic acid or polyhydric alcohol responsible for covalent cross-linking. Generally, the bridge bonds between the different polymer chains allow nanomaterials to form a three-dimensional structure. Mostly used crosslinkers are glutaraldehyde, genipin, polyethylene glycol, or monofunctional agents [11].

For the first time, Savin et al. [48] prepared polymeric nanocarriers based on the chitosan grafted-poly(ethylene glycol) methacrylate derivative. The technique selected for the preparation of the micro-nanoparticles (MNPs) was a double crosslinking (ionic and covalent) process in the reverse emulsion which provided the mechanical stability of the polymeric nanocarrier. The covalent cross-linking process was carried out by adding in the glutaraldehyde, the ionic crosslinking agent was TPP or Na_2_SO_4_.

#### 3.2.2. Self-Assembly

Self-assembly is described as the association of certain molecules, macromolecules, or composite materials with themselves to form 3D networks or other structures with new distinguishing properties. The self-assembling process can take place at the molecular or supramolecular level. It can occur by self-association or by an association with other structures through interactions such as hydrogen bond, van der Waals forces, and ionic or hydrophobic interactions. It can also be caused by an inclusion/complexation mechanism, like the iodine inclusion complex with starch [68].

Self-assembled NPs can be formed in the aqueous media of amphiphilic polymers. Hydrophobic interactions between amphiphilic polymer components tend to minimize interfacial energy and allow nanoparticle formation [54]. Although CS is not an amphiphilic bio-polymer, the self-organizing NPs can be obtained by a structural modification of CS, particularly the introduction of hydrophobic moieties into the CS molecules by grafting, to modify its hydrophobic–hydrophilic balance. Such balance is promoting self-assembly in an aqueous or polar medium. The grafting agent can be a hydrophobic moiety, such as cholesterol, cholic, and deoxycholic acid, 5β-cholanic acid, phthaloyl, polyester, fatty acids, etc. [69]. Hydrophobic molecules are usually immobilized on CS by N-acylation, N-alkylation, O-alkylation, and Schiff-base reaction [11]. CS self-assembled NPs are particularly useful for encapsulating hydrophilic as well as lipophilic drugs [68].

##### Ionic Cross-Linking (Ionic Gelation)

Ionic cross-linking (or also found in the literature as ionic gelation) is one of the most widely used methods for the preparation of CS NPs. It has been extensively used for loading biopharmaceuticals. A major advantage of this method of physical CS crosslinking is related to its mild conditions attained. This method avoids the use of organic solvents, high temperatures, or vigorous agitation. Therefore, it can retain bioactive molecules such as proteins, DNA, etc. [54,70].

CS as a cationic polysaccharide can gel with negatively charged compounds or specific polyvalent polyanionic molecules to form NPs. This combination leads to the formation of electrostatic interactions between opposite charges of the components. At acidic pH, there is a spontaneous formation of particles in the submicron size. As there is a formation of gels due to ionic linkage, this method is also known as the ionic-gelation method [11,52,54].

Two main groups have been used as anionic cross-linkers: (i) anionic low-MW molecules, like cyclodextrin derivatives or tripolyphosphate (TPP), and (ii) anionic macromolecules, such as poly-y-glutamic acid, dextran sulfate, hyaluronic acid, and sodium alginate [11,70].

**Figure 3:**
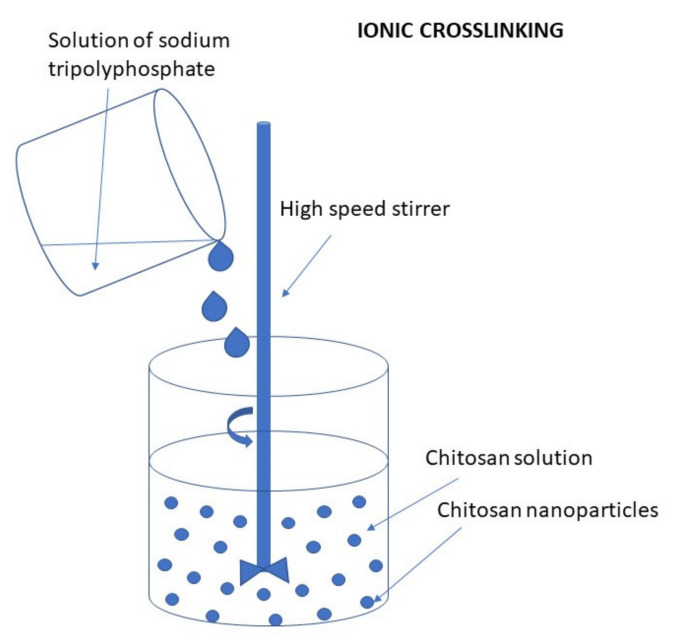
Scheme illustrating preparation of CS NPs by ionic crosslinking with TPP.

TPP is the earliest and the most used cross-linking agent developed for ionic crosslinking with CS. Scheme illustrating preparation of CS NPs by ionic crosslinking with TPP is in Figure 3. When using TPP as a cross-linking agent, the NPs are prepared at room temperature by dissolving CS in a diluted solution of glacial acetic acid and adjusting pH to the expected value by the addition of NaOH. The TPP solution is added dropwise to the CS solution with constant stirring until it forms an opaque suspension, which indicates the formation of particles with an average diameter controlled by the molar ratio of CS/TPP. Factors influencing the mechanism of CS NPs formation by this method are dependent on parameters like the degree of acetylation and MW of CS, intrinsic viscosity, concentration, and the molar ratio of -NH_3_^+^/TPP. Other factors may also influence the properties of the NPs, such as operating temperature, stirring speed, and flow rate of TPP addition. They can affect particle size and polydispersity, as they appear to significantly reduce the amount of NP aggregation. Smaller particles can be isolated from larger particles by filtration and ultracentrifugation [54].

Sang et al. [71] used the ionotropic gelation method to prepare CS NPs using polyphosphate crosslinking agents, namely TPP, phytic acid (PA), and sodium hexametaphosphate (SHMP). The encapsulation efficiency of myricetin (MYR) in the CS NPs crosslinked by PA and SHMP (67.3 ± 0.4% and 62.2 ± 0.2%, respectively) was significantly higher than that in the CS NPs crosslinked by TPP (47.7 ± 0.1%) (*p* < 0.05); their drug release rate (43.7 ± 5.1% and 44.0 ± 3.7%, respectively) was also significantly slower than that of MYR-CS NPs crosslinked by TPP (103.4 ± 4.0%) (*p* < 0.05). Furthermore, a strong mucoadhesiveness of the CS NPs crosslinked by PA was shown by a fast increase in the turbidity value and a sharp decrease in the zeta potential in the mucin solution test.

Pan et al. [72] studied the relationship between the degree of crosslinking and the properties of CS NPs. They established a potassium polyvinyl sulfate (PVSK) titration method for the determination of free amino group in CS, which showed that there was a window effect in the crosslinking degree and particle size. They examined three kinds of molecular weight CS cross-linked by different content of TPP; the NPs with a moderate crosslinking degree were smaller. The minimum inhibitory concentration (MIC) and minimum bactericidal concentration (MBC) data showed the moderate degree of cross-linking NPs had strong antibacterial properties. IR analysis revealed that the interactions between CS NPs with a different crosslinking degree and TPP were different. X-ray diffraction analysis showed that the cross-linked CS NPs were amorphous. In conclusion, the crosslinking degree of the TPP cross-linked CS NPs was related to the particle size and antibacterial properties.

Colloidal chitosan/tripolyphosphate (CS/TPP) particles prepared by ionic gelation have attracted significant attention as potential delivery vehicles for drugs, genes, and vaccines. Yet, there have been several fundamental studies that showed these particles disintegrate at physiological pH (pH 7.2–7.4) and ionic strength levels. Echeverri-Cuartas et al. [73] explored the possibility of improving a TPP-crosslinked CS NP stability through a chemical modification of CS. Specifically, CS samples with either 76% or 92% degrees of deacetylation (DD) were grafted with either polyethylene glycol (PEG), or folic acid (F). Within the degrees of substitution (~1% for PEG, and 3% and 6% for F), neither PEG nor F qualitatively improved CS NP stability at physiological conditions (pH 7.2). Another way to improve this stability was explored by Abdelgawad et al. [74]. They used hexametaphosphate (HMP) instead of TPP as a cross-linking agent. HMP is a hexavalent molecule in the neutral and slightly basic medium which offers more binding sites readily available for interaction with CS. It is thought that increasing the availability of the binding sites in the HMP molecule would result in stronger ionic complexation with CS cationic moieties. Consequently, such stronger binding should improve particles’ stability and lead to average size reduction. A comparative study between CS/TPP and CS/HMP NPs under different complexation conditions was conducted to investigate the effect of HMP on NPs formation. Although the drug loading efficiency of BSA, 96.3%, was higher than when using TPP, 91.87%, the TPP cross-linked particles showed superior stability upon storage. Cai and Lapitsky [75] proved that the particles could be stabilized by their bioactive payloads. They studied the enhancement of the CS/TPP particle stability at physiological ionic strengths, using 150 mM NaCl (pH 5.5) and PBS (pH 6.0) as the dissolution media when associating the CS/TPP particles with model anionic proteins (α-lactalbumin, α-LA, and bovine serum albumin, BSA) and polynucleotides (DNA). Light scattering and UV–VIS spectroscopy revealed that anionic protein uptake had no impact on particle stability. It was likely due to the relatively weak protein/particle binding at near-physiological ionic strengths, which caused the protein to be rapidly released. Conversely, a DNA uptake increased the CS fractions persisting in a complexed/particulate form in model dissolution media, with the DNA remaining largely complexed to the CS at all investigated conditions. The results suggested that, while most bioactive payloads do not interact with CS strongly enough to stabilize the CS/TPP particles, these particles can be stabilized to dissolution through the incorporation of polyanions.

The literature on the release performance of CS/TPP micro- and nanoparticles is filled with conflicting results, with some reporting nearly instantaneous release, while others showing the release to be sustained for up to multiple days. To resolve these opposing findings, Cai et al. [76] examined several experimental artifacts that may arise during such in vitro experiments and showed that conflicting findings on release from CS/TPP particles can arise from: (1) incomplete particle separation from the release media upon centrifugation; (2) irreversible particle coagulation; and (3) failure to maintain sink conditions. They provided guidelines for obtaining more reliable release profiles for CS/TPP micro- and nanoparticles and other/related colloidal carriers. To minimize problems with an incomplete particle separation, high centrifugal forces, and long centrifugation times (which exceed those used typically) are needed. A glycerol bed can help to prevent particle coagulation; however, this method no longer appears to work for CS/TPP micro- and nanoparticles after the first 3–4 centrifugation/redispersion cycles (at least under the centrifugation conditions needed for their full separation with the setup used by the authors). For particles with fast release rates (such as the colloidal CS/TPP particles explored in their work), frequent sampling/solvent replacement must also be used to capture the release kinetics and maintain sink conditions. Due to the long centrifugation times required to fully sediment the particles, however, this ultracentrifugation-assisted solvent re-placement method cannot accurately determine the release profiles. As an alternative, sink conditions can likely be achieved by diluting the dispersions in large volumes of release media and collecting small dispersion portions (after removing the particles with a filter) to quantify the release. The authors demonstrated that it is essential to use release media with physiologically relevant ionic strength levels, as a release from CS/TPP particles can be highly salt-sensitive.

##### Polyelectrolyte Complex (PEC)

A suitable way to generate self-assembled CS NPs is through the formation of polyelectrolyte complexes with polyanions. Polyelectrolyte complexes (PECs) are formed when the solutions of two polyelectrolytes carrying complementary charges (i.e., a polycation and a polyanion or their corresponding salts) are mixed. In this case, PEC formation is mainly caused by the strong Coulomb interactions between the oppositely charged polyelectrolytes. The formation of complexes brings about at least a partial charge neutralization of polymers. In addition, inter-macromolecular interactions such as Van der Waals forces, hydrogen bonding, and hydrophobic interactions are involved in the formation of PEC structures as well [77].

**Figure 4:**
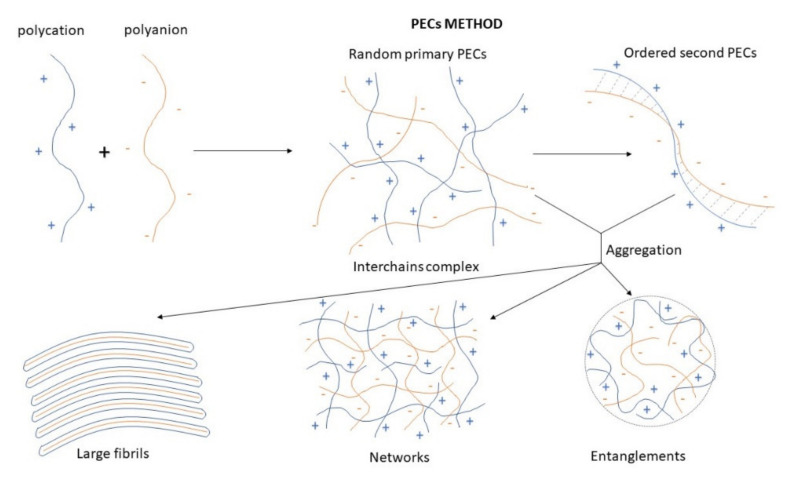
Scheme of the different steps of the polyelectrolyte complexes formation.

The PECs formation generally involves two or three steps. The scheme of the different steps of the polyelectrolyte complexes formation is shown in Figure 4. The first step is instantaneous and leads to the formation of a random primary complex with significant distortions of the configuration of polymer chains. Then, the secondary complex is formed by rearrangement of existing linkages within intracomplexes. It involves the formation of new bonds, e.g., electrostatic bonds, hydrogen bonds, hydrophobic interactions, etc. Finally, under certain conditions, primary and secondary complexes can aggregate (probably by hydrophobic interactions) and lead to various stable structures: entangled aggregates, fibrils, ordered networks, etc. Preparation of CS PECs is a safe green process in water, with no organic solvents nor chemical cross-linkers or surfactants used. It is a simple fabrication process where spontaneously mixing the oppositely charged polyelectrolyte solutions leads to interpolymer ionic condensation [78].

The stability of PECs depends on several factors including the degree of ionization within each polyelectrolyte with an opposite charge, the density of the charges presents in the structure of polyelectrolyte, concentration, and proportion of polyelectrolytes in the mixture, a sequence of additions in the mixture and the type and location of ionic groups. In polymer chains, the stability is also correlated with the MW of the polyelectrolytes, the flexibility of the polymer chain as well as the temperature, ionic resistance, and pH of the reaction medium. The structure of CS-based nanoPECs is highly impacted by the presence of salt. A high concentration of salt may disrupt the integrity of the nanoPECs and dissociate the polyelectrolytes. When the positively charged surface of CS interacts with anionic biopolymers such as sodium alginate, carboxymethylcellulose, chondroitin sulfate, dextran sulfate, poly(acrylic acid), pectin, carrageenans, heparin, and other polyions, various CS-based PECs can be formed [78].

Several conditions, such as pH (particularly important in weak polyelectrolytes), ionic strength, and the mixing rate, should be adjusted to the particular CS-polyanion pair system selected since these variables will also influence the size and charge of CS NPs. Different preparation methods will result in diverse kinds of CS NPs, which can be classified as nanoaggregates, nanocapsules, or nanospheres. The particular procedure selected can be largely determined by the water solubility of the active agent that will be encapsulated and the polyanion used [78].

By chitosan’s favorable properties, especially the mucoadhesive nature and absorption enhancement capability, as well as the safe and green manufacturing process of colloidal PECs, CS-based nanoPECs are particularly appropriate for the delivery of sensitive biological molecules such as vaccines and proteins like insulin via the mucosal route. Cationic CS-based PECs can also promote the internalization of the loaded cargos into cells and subsequently escape the lysosome via the proton sponge effect, therefore exhibit high therapeutic potential, e.g., in anti-tumor applications [77].

#### 3.2.3. Emulsion Technique

In general, the particles formed in the emulsion system have structures of nanocapsules, high drug loading efficiency, and good bioavailability [79]. NPs can be obtained using the nanoemulsion technique in various ways. Some of them are low power emulsification methods but emulsification processes usually require high mechanical energy to obtain small droplet sizes. The most common emulsification method used for the preparation of CS NPs is the water-in-oil emulsion, where an aqueous CS solution is emulsified in an oil phase. Surfactants are applied for stabilizing the formed particles [9,80]. 

Briefly, in the first step, a dispersion of CS in a glacial acetic acid solution is prepared as an aqueous phase. Then, a drop of a hydrophobic solvent, such as cyclohexane, cyclohexanol, soybean oil, or another solvent with similar properties is added to the CS solution, all mixing continuously. In the final step a surfactant, for example, Triton X-100 or Tween is being added to the resulting mixture under constant stirring until the mixture becomes translucent. This indicates the formation of emulsion-containing NPs [81]. 

To skip a step in the process, the surfactant can be pre-mixed in one of the solutions. Shahab et al. [82] prepared CS-coated polycaprolactone NPs by a single-step emulsification technique with a modification compared to the previously established method. The organic phase was prepared by dissolving polycaprolactone and dorzolamide in dichloromethane. Separately, the aqueous phase was prepared by solubilizing CS in 1% acetic acid solution (pH 5.0) with polyvinyl alcohol (PVA). The organic phase was added dropwise to the aqueous phase with constant stirring for 6 h to evaporate the organic solvent. The prepared NP dispersion was sonicated, and the created NPs were further lyophilized using mannitol as cryoprotectant. 

##### Emulsion Droplet Coalescence (Emulsion Crosslinking and Precipitation)

**Figure 5:**
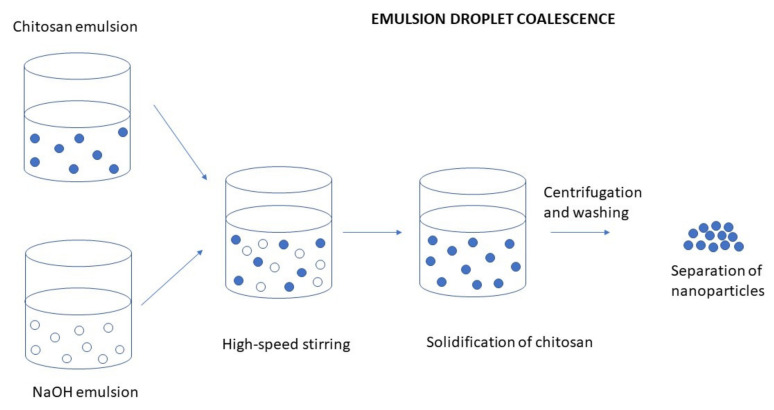
Preparation of CS NPs by emulsion droplet coalescence method.

There are various modifications in the preparation of an emulsion. For example, the addition of gelling agents such as chlorides, TPP, or polycations can be used. An emulsion droplet coalescence method is based on the principles of emulsion crosslinking and precipitation methods. Preparation of CS NPs by emulsion droplet coalescence method is schematically illustrated in Figure 5. In the first step of the procedure, two emulsions are prepared; one containing an aqueous solution of CS and liquid paraffin along with a drug and the second one containing an aqueous alkaline (e.g., NaOH) solution of CS in liquid paraffin oil. Both the emulsions are mixed under a high-speed stirring, resulting in random collisions of droplets of each emulsion. Coalescence of CS droplets with NaOH droplets takes place and thereby precipitation of CS in small solid particles [83].

To intensify the emulsion crosslinking process for the synthesis of CS NPs, Zhang et al. [84] developed a controlled hydrodynamic cavitation technique. Their work demonstrated the feasibility of hydrodynamic cavitation as an energy-efficient approach for intensifying the emulsion crosslinking synthesis of CS NPs compared with ultrasonic horn and conventional drop-by-drop process. The novel approach can greatly reduce the particle size and distribution of the synthesized CS NPs. Although the emulsification method facilitates particle size control, strong crosslinking agents are usually used in this process and complete removal of unreacted crosslinking agents can be an issue [85].

Riegger et al. [86] investigated the impact of glutaraldehyde concentration and molecular weight (MW) of six commercially available, highly deacetylated CS on the nanoparticle formation by emulsion crosslinking technique. Increasing MW of CS resulted in larger particle sizes ranging from 109.9 nm for the lowest MW up to 200.3 nm for the highest MW. An adsorption capacity of up to 351.8 mg g^−1^ diclofenac for low MW CS NPs was observed and all CS NPs showed superior adsorptions when compared to an untreated CS. Hence, the results suggested the use of the prepared CS NPs as promising adsorbers for diclofenac and carbamazepine.

##### Emulsification Solvent Diffusion

**Figure 6:**
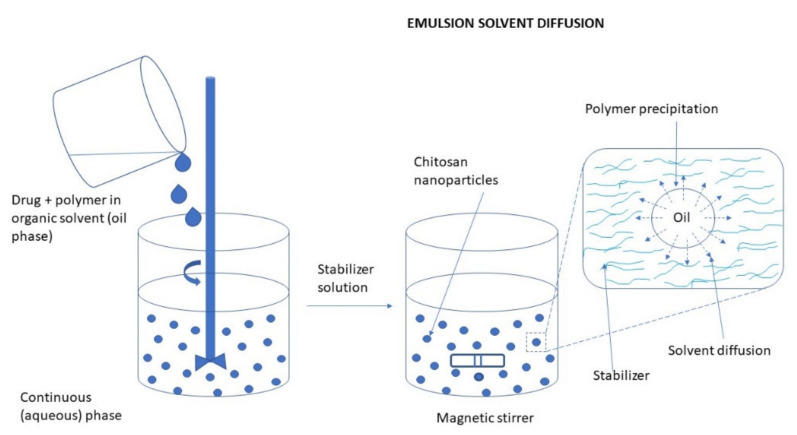
Preparation of CS NPs by emulsion solvent diffusion method.

The process of emulsion solvent diffusion is based on the partial miscibility of an organic solvent as an oil phase with water. Preparation of CS NPs by emulsion solvent diffusion method is schematically illustrated in Figure 6. A drug is dissolved in an organic solvent and is added upon injection to an aqueous phase containing CS with a stabilizing agent such as lecithin or poloxamer. As a result, the simple water-in-oil emulsion is formed upon high-pressure homogenization. The emulsion is then diluted with a large amount of water to overcome organic solvent miscibility in water. Finally, NPs are formed through CS precipitation due to reduced CS solubility as acetone diffuses into the aqueous phase. This leads to the formation of small particles. As the concentration of water-miscible solvent increases, a decrease in the size of particles can be achieved. The isolation of NPs is made using the centrifugation process. This method is suitable for hydrophobic as well as hydrophilic drugs. In the case of hydrophilic drugs, a multiple water/oil/water emulsion (e.g., double emulsion) needs to be formed with the drug dissolved in the internal aqueous phase. The major drawbacks of this method include harsh processing conditions (e.g., the use of organic solvents) and the high shear forces used during NP preparation [70,87,88].

Often, this method is used for the preparation of PLGA NPs with CS as a coating material. Liu et al. [79] prepared dual drug-loaded NPs by the double emulsification solvent evaporation method. Salmon calcitonin (sCT) was dissolved in water as an internal aqueous phase. Puerarin (PR) and PLGA were dissolved in acetone, and then Tween-20 was added to the mixture to prepare an organic phase. PVA solution and CS solution were mixed as an external aqueous phase. Subsequently, the internal aqueous phase was added to the organic phase and W/O emulsion was obtained by sonication. W/O/W emulsion was formed by adding the W/O emulsion into the external aqueous phase. Finally, vacuum evaporation was conducted to remove the organic solvent, and sCT-PR-CS/PLGA NPs were recovered. The NPs solution was placed in a vacuum dryer over 12 h after rotating evaporation to remove residual acetone.

##### Emulsification Solvent Evaporation

**Figure 7:**
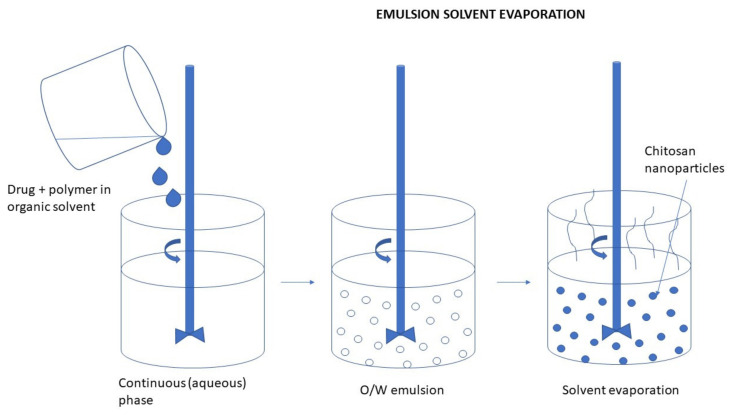
Preparation of CS NPs by the emulsion solvent evaporation method.

This is one of the most popular methods for the encapsulation of a drug within the water-insoluble polymer. It consists of preparing an emulsion, with a different external phase depending on the nature of the polymer and the drug used for the encapsulation and evaporating the solvent with the subsequent formation of the nanospheres [87]. The preparation of CS NPs by the emulsion solvent evaporation method is depicted in Figure 7.

Essa et al. [89] prepared CS NPs using a single-emulsion oil in water (O/W) solvent evaporation approach. CS was agitated in glacial acetic acid to produce the aqueous phase. The organic phase was prepared by dissolving PLGA in either acetone, chloroform, or dichloromethane and added dropwise under stirring to the aqueous phase. The emulsion was left to agitate under magnetic stirring for 8 h to allow complete evaporation of the organic solvent. Thereafter the formulation was centrifuged and washed with distilled water to remove any unentrapped drug molecule. The resultant pellets were redispersed in distilled water and then sonicated again to produce free-flowing NPs that were frozen at −80 °C and then lyophilized for 24 h.

#### 3.2.4. Reverse Micellar Method

**Figure 8:**
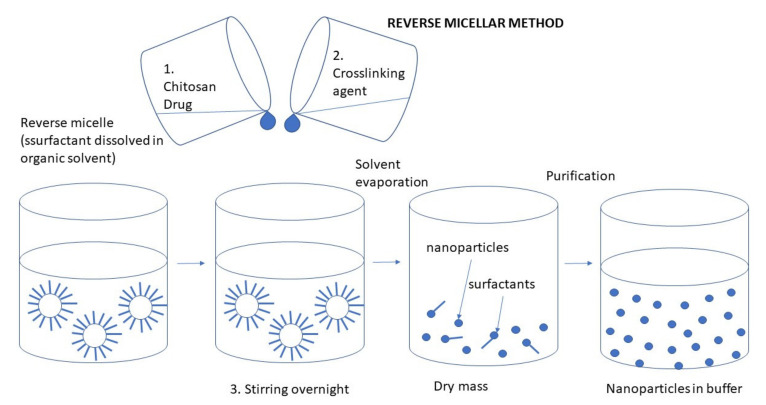
Preparation of CS NPs by the reverse micellar method.

This method is based on the formation of the NPs in an aqueous core of reverse micellar droplets, followed by cross-linking with glutaraldehyde. It is used to prepare ultrafine polymer NPs with a narrow size range. The preparation of CS NPs by the reverse micellar method is shown in Figure 8.

The formation of reverse micelles is carried out by adding a surfactant to an organic solvent. An aqueous solution of CS with drug and a glutaraldehyde solution is then added to that the organic micellar solution under constant stirring to avoid turbidity. Water is then added to maintain the mixture in an optically transparent microemulsion phase. The amount of water is increased to obtain NPs of larger size [51]. To attain complete cross-linkage for CS, it is advised to maintain the stirring overnight. The organic solvent is evaporated and the created NPs can be harvested by precipitation with a suitable salt. The solution is then centrifuged, and the aqueous supernatant is dialyzed for about 1 h and lyophilized to dry powder [9,88].

The advantages of this method are associated with the ability to produce a small particle size with a narrow size of the distribution. On the other hand, the disadvantages include the laborious and time-consuming process and the presence of organic solvent and surfactant [88].

Orellano et al. [90] studied the effect of the micellar interface on the synthesis of CS NPs. Both benzyl-n-hexadecyltrimethylammonium chloride (BHDC) and 1,4-bis-2-ethylhexylsulfosuccinate (AOT) reverse micelles were assessed since there were found remarkable differences between their interfacial water entrapped structures. The CS NPs obtained under different conditions were assessed in terms of their ability to solubilize curcumin, whose numerous therapeutic properties are somewhat countered by its poor solubility in water. The results showed that the crosslinking reaction took place in the micellar interface and was more effective in the AOT reverse micelles. This difference in effectiveness can be attributed to the different positions that CS acquired in each of the two reverse micelles tested. Finally, the NPs notably enhanced the water solubility of curcumin, and particle size was the main determining factor for encapsulation efficiency.

#### 3.2.5. Drying Methods

During drying processes water or solvents are separated through evaporation from liquids, solids, or semi-solids and the resulting vapor is collected by vacuum. Hot air, a microwave oven, spray drying, freezing, supercritical drying, and natural drying with air are common drying methods. The techniques of spray drying and supercritical drying are often used in the production of CS NPs because they are fast, simple, continuous, reproducible, and adapt well to the active material. They are scaled down in one phase without modification. These techniques are important and can achieve particles of various sizes and high stability [91].

##### Spray Drying

The spray drying technique is the most common drying method used to prepare NPs based on CS. It is based on the use of a flux of hot air to dry spray drops. This method requires the preparation of an aqueous CS solution in which the drug is dispersed. The addition of natural cross-linking agents improves the biocompatibility and performance of drugs. A TPP cross-linking agent is usually used to overcome the problem of poor solubility of non-cross-linked CS in aqueous media. The use of an adequate excipient reduces the risk of thermal degradation during the spray drying process. For example, polysorbate 20 can be used as a protective agent of proteins from denaturation due to high shear rates during the atomization step [70].

**Figure 9:**
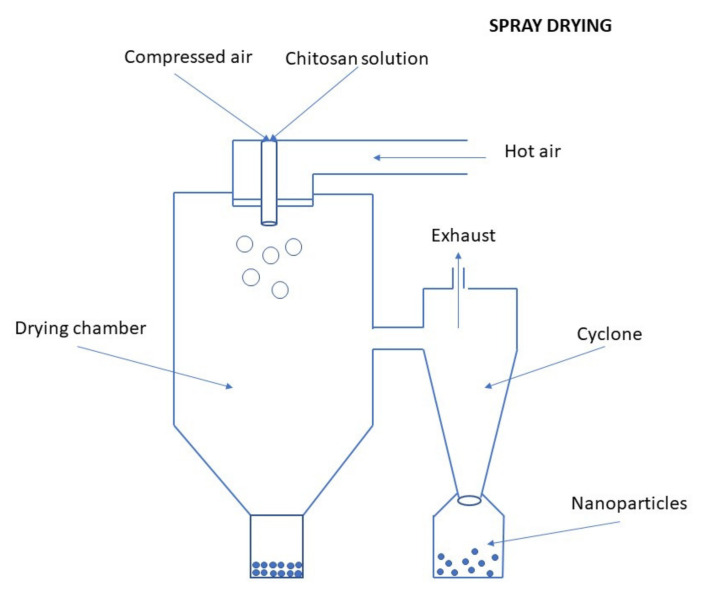
Preparation of CS NPs by the spray-drying method.

The preparation of CS NPs by spray drying method is schematically illustrated in Figure 9. The solution of polymer with crosslinker is sprayed into a drying chamber through a nozzle and it is atomized in a stream of hot air. This results in the formation of small droplets from which the solvent evaporates and form free-flowing particles. The resulting particles have a smooth surface, aspherical shape, and reduced size distribution, and they also exhibit good drug stability with high efficiency when the drug is turned on. Different factors such as the size of the nozzle, spray flow rate, inlet air temperature, atomization pressure, the extent of crosslinking, etc. influence the particle size [9,92].

The spray drying technique provides a convenient, one-step, and protein-friendly method for protein-loaded CS MPs or NPs [91].

Ozturk et al. [93] prepared CS NPs containing dexketoprofen by spray-drying method and tested them for anti-inflammatory activity.

##### Supercritical Fluid Drying

**Figure 10:**
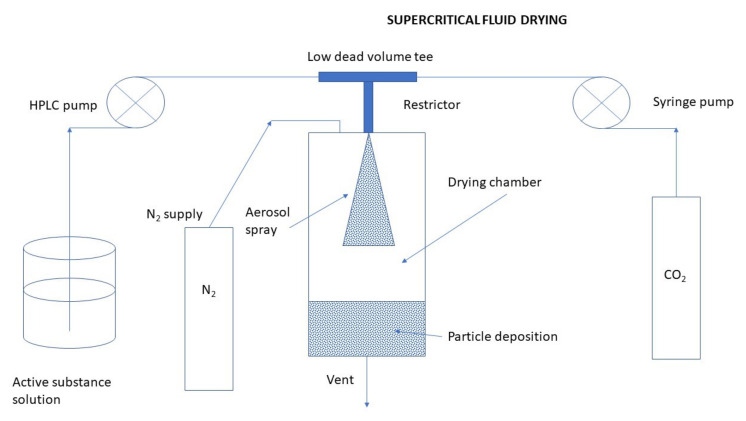
Preparation of CS NPs by the supercritical fluid drying method.

Usually, a supercritical fluid process with carbon dioxide (CO_2_) is used to prepare diverse pharmaceutical applications at lower pressure and temperature. An illustrative scheme of the preparation of CS NPs by the supercritical fluid drying method is depicted in Figure 10. The process is nontoxic, nonflammable, and ensures the minimal decomposition of drugs like proteins. It provides also the possibility to prepare MPs especially those oriented for inhalation [70].

Peng et al. [94] prepared DOX-loaded CS NPs using supercritical fluid assisted atomization introduced by a hydrodynamic cavitation mixer (SAA-HCM) from an aqueous solution. The influences of solution concentration, CO_2_/solution ratio, mixer pressure, CS/DOX ratio, and CS molecular weight on the particle morphologies and sizes were investigated in detail. FT-IR results showed that the structure of DOX was not changed after the SAA-HCM process. The in vitro drug release behavior conducted in the media with pH of 4.5, 6.5, and 7.4, respectively, was found to be strongly pH-responsive. The in vitro cytotoxicity profiles revealed the activity of DOX was well maintained after being loaded into the CS NPs. The SAA-HCM process was demonstrated to be a promising technique for the one-step production of polymer/drug composite NPs suitable for cancer drug delivery from aqueous solutions.

In another work by Peng et al. [95] prepared nano-in-microparticles composed of CS NPs and mannitol, using a modified supercritical CO_2_ assisted atomization (SAA-HCM) technique. Drug-free CS-based NPs were prepared using an ionic gelation method with TPP. CO_2_ from a cylinder was turned into liquid flowing through a cooling bath and sent to the mixer via a high-pressure pump. Meanwhile, the liquid nanosuspension, which was made up of the resuspended NPs in the D-mannitol solution at different weight ratios, was loaded into the mixer by a high-pressure pump. After mixing, a nozzle (200 μm) was used to atomize the mixture into the precipitator. The nano-in-microparticles were formed after evaporation of the solvent from the nanosuspension droplets by the drying of hot nitrogen. Finally, the particles were collected at the bottom of the precipitator using a cyclone separator for further analysis.

##### Electrospraying Technique

**Figure 11:**
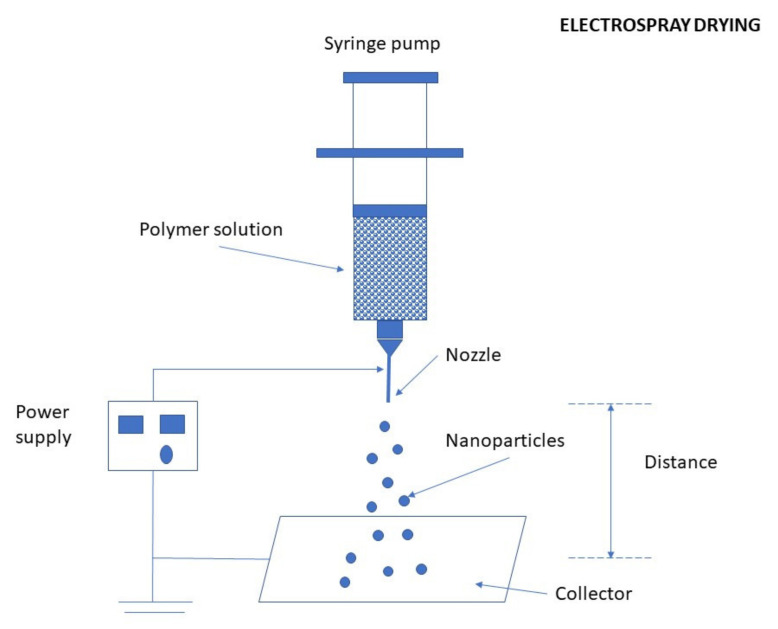
Preparation of CS NPs by the electrospraying method.

Electrospraying is an electrohydrodynamic process used in the formation of NPs. The preparation of CS NPs by the electrospraying method is schematically illustrated in Figure 11. A high voltage electric field is applied into a polymeric solution flowing out of the nozzle to break it down to very fine nano-sized droplets possessing the same charge which assists their dispersion and prevents possible coagulation [96]. By altering specific variables such as applied electric voltage, solution flow rate, the distance between needle tip and collector, and the type of collector, the particle size, and morphology can be tuned [97].

Electrospraying has several advantages and very few limitations. It is a simple and one-step technique of a low cost. The entire spraying procedure can be performed at ambient temperature and pressure conditions, making a great benefit for the fabrication of carriers for the delivery of sensitive (high MW) bio-molecules/actives or living cells. Additionally, the probable absence of an external medium or solvent, that may cause migration or dissolution of the hydrophilic carrier, may greatly benefit in achieving specific use. The absence of coalescence in the case of electrospray droplets, due to the electrostatic charge repulsion, will result in uniform spread across large surface areas. Hence, monodispersed NPs can be easily obtained by this technique. The main disadvantage, however, is associated with relatively low product yields [88,98].

CS NPs have been recently prepared using an electrospraying technique by Wang et al. [99]. They prepared sample solutions for electrospraying by dispersing CS in an aqueous solution of glacial acetic acid. Then, different quantities of tea polyphenols (TP) were slowly added to the CS solution to obtain different mass ratios. An aqueous solution of TPP was prepared and continuously pushed by a syringe pump at different flow rates, using a homemade electrospinning/electrospraying apparatus. The NPs were collected by centrifugation and lyophilized overnight to remove any solvent and water residues.

#### 3.2.6. Precipitation/Coacervation

**Figure 12:**
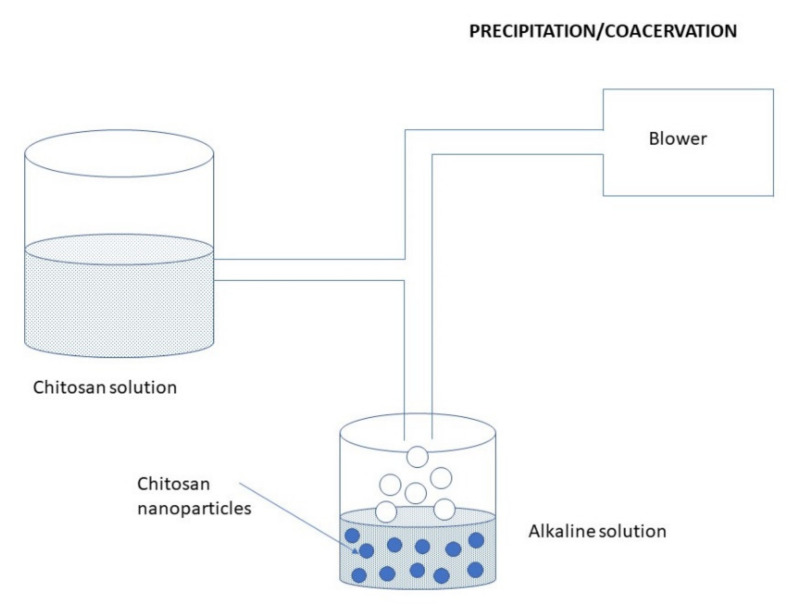
Preparation of CS NPs by the precipitation/coacervation method.

This method is based on the utilization of characteristic physicochemical properties of CS, i.e., its insolubility in alkaline pH medium and thereby forming a precipitate. Preparation of CS NPs by precipitation/coacervation method is schematically illustrated in Figure 12. In this method, a CS solution is sprayed into an alkali solution such as NaOH or ethylenediamine, using a compressed air nozzle forming coacervate droplets [52,70]. Afterward, the separation and purification of the particles are performed by either filtration or centrifugation, followed by successive washing with hot and cold water. Another (alternative) method uses a sodium sulfate solution which is added dropwise to an acidic solution of CS containing surfactant while undergoing stirring and continuous sonication for 30 min [100].

#### 3.2.7. Microfluidic Method

Conventional techniques including precipitation, spray drying, homogenization usually suffers from limitations, particularly due to the lack of control over the fabrication processes. Specifically, the fabricated CS-based materials have high batch-to-batch variations in physicochemical properties, such as the particle size, size distribution, and surface charge. Moreover, the structures of the fabricated CS-based materials cannot be elaborately tuned according to demand. Consequently, the accurate relationships between physicochemical properties and performance of the fabricated CS-based materials cannot be systematically investigated, which further hampers their applications. Besides these drawbacks, the conventional methods are highly complex and therefore difficult in scale-up production [101].

**Figure 13:**
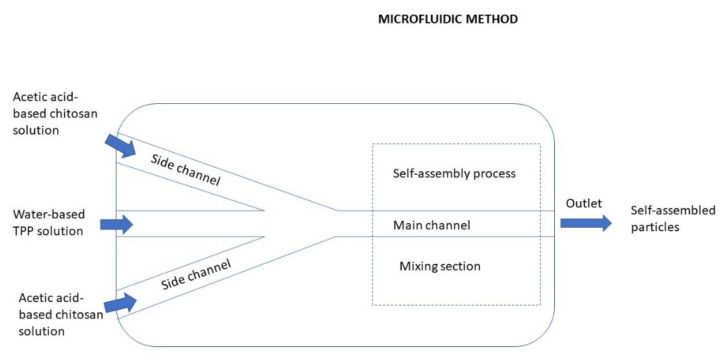
Preparation of CS NPs by the microfluidic method.

Recently, microfluidics has been demonstrated as one of the most promising platforms to fabricate CS-based NPs with monodisperse size distribution and accurately controlled morphology and microstructures. The preparation of CS NPs by the microfluidic method is depicted in Figure 13. Microfluidic technology has precise fluid control, various shapes of a microchannel, and a multi-channel programmed mixing process, which provides a new opportunity for the synthesis of various types of CS with particle morphology, uniform particle size distribution, and batch quality repetition control. In other words, it is possible to achieve a series of preparation and processing that are difficult to complete with conventional methods, including higher particle size uniformity and precise control of the structural assembly. Fluids manipulated by microfluidic technology in these narrow channels have many unique properties, one of the most important is laminar flow [102,103]. In general, flows of common liquids in typical microfluidic channels (<100 μm) are characterized by the laminar state. Specifically, the equivalent diameter of microfluidic tubes is usually micron-sized or even nano-sized, and laminar flow can be achieved. Due to the steady streamlines of the laminar flow, fluids in different microfluidic devices (divided into three basic types, namely T-type, Y-type, and coaxial flowing) can be stretched or fractured to form monodispersed droplets or a liquid jet. Furthermore, the use of multiple microfluidic tubes in series can also provide the possibility to prepare particle shells and fiber sheaths [104].

In the process of preparing CS NPs by a microfluidic technology, the characteristics of CS itself are particularly important. For example, the concentration of CS, molecular weight and deacetylation degree of CS, and type of derivatives of CS can affect the physical properties of the CS solution like density, viscosity, and surface tension [105]. Under the same flow rate and pressure, the thickness of the CS liquid layer and the size of the droplets in the microfluidic channel may also be affected [101].

Lari et al. [106] synthesized a novel crosslinked carboxymethyl chitosan NPs (CMC NPs) containing metformin hydrochloride (MET) using microfluidics and evaluated their performance for diabetes therapy. The microfluidic fabricated NPs exhibited slower and gradual release of the drug in comparison to a bulk method. The animal study on the rats indicated that the produced NPs were able to prevent weight loss, decrease blood glucose levels, and regenerate the pancreatic islets. The histopathological analysis showed that the MET-loaded CMC NPs had a better effect on the diabetes treatment compared with free MET. Therefore, the NPs synthesized by the microfluidic method seems to be a potent and effective therapeutic tool for diabetes therapy.

Faharani et al. [107] attempted to set up a new approach to preventing fluid impassability as a result of microchannel blockage by introducing an acidic TPP solution. Thanks to this approach, the life span of the microfluidic chip also was increased, and no microchannel blockage occurred during the production process. Berberine as a model anticancer drug was successfully encapsulated in the chitosan/TPP NPs in a reproducible manner with optimized physical properties. Compared to other methods for delivering berberine via CS NPs prepared by bulk methods, the microfluidic produced berberine-CS/TPP NPs exhibited an enhanced drug loading content, narrow PDI (polydispersity index), spherical morphology, and controlled drug release profile.

### 3.3. Chitosan Based-Nanocomposites, Types, Their Properties, and Utilization

Improvement of physicochemical properties of CS can also enhance the utility of CS NPs. CS NPs have different properties which can further be enhanced for more efficient drug delivery. For improving the intestinal solubility of CS, N-trimethyl CS chloride has been produced. To increase the mucoadhesiveness of CS, NPs with thiolated CS were formulated. pH sensitivity can be achieved by grafting carboxylated CS with poly (methyl methacrylate). For specific applications, physical modifications can be performed in CS by blending, i.e., physically mixing two or more polymers. For example, blending CS with polyvinyl alcohol improves the mechanical and barrier properties of CS [52]. Thus, CS-based nanocomposites, combining native or derivatized CS with other organic polymers (Section 3.3.1) or inorganic substances (Section 3.3.2), can significantly enhance drug delivery performance in various specific situations. One of the most important/advanced areas of their use is represented by stimuli-responsive (CS-based nanocomposite) materials for drug delivery (Section 3.3.3). Stimuli-responsive CS-based materials do not represent exclusively nanocomposites but also simpler NPs formulated from some CS derivatives suitable for this purpose. Therefore, also such specific CS NPs are presented here along with CS nanocomposites via recent application examples.

#### 3.3.1. Chitosan-Polymer Nanocomposites

A blend of natural or synthetic polymers represents an innovative class of materials and has paid much attention, especially for biomedical applications. Although natural polymers have good biocompatibility and biodegradability for such applications, they can have often low solubility, mechanical and thermal stability limitations. Thus, the blending of natural and synthetic polymers results in new materials exhibiting combinations of properties about both polymers, that could not be obtained by individual polymers. In drug delivery systems, the blending of polymers provides stable, controlled release increases their encapsulation and loading capacity. Furthermore, the incorporation of nanofillers to the blend matrix additionally controls the release profile of the drug by acting as a diffusion barrier [18,48]. In the literature, CS is often combined with synthetic polymers such as polyethylene glycol (PEG), polyvinylalcohol (PVA), and natural polymers such as alginate, dextran, curdlan, carrageenan, caseinate, and pectin.

##### Synthetic Polymers

**Figure 14:**
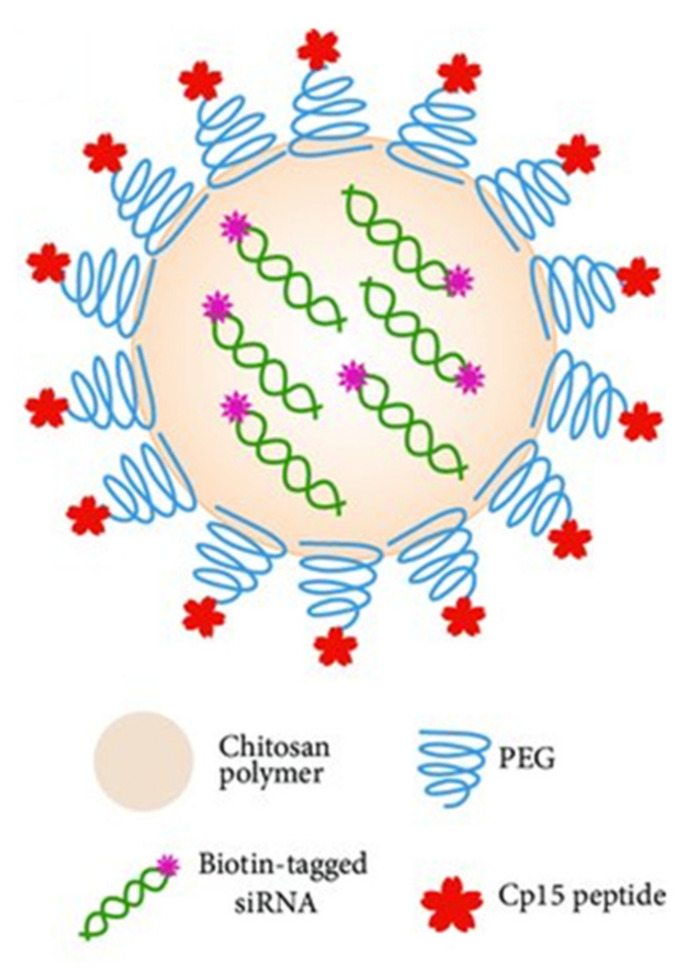
A nanoparticle formulation with a core formed by CS polymer, surface-functionalized with PEG, and a cell-targeting peptide (CP15). The formulation encases a biotin-tagged siRNA [109]. Reproduced with permission from Hindawi (Copyright©2013).

PEGylated CS derivatives enhance the solubility of CS. In addition, they have lower cytotoxicity, higher ductility, and high stability in body fluids, which enable them to be applied in highly efficient drug delivery, gene delivery, stimuli-responsive hydrogel formation, and nanofiber formation [108]. A nanoparticle formulation with a core formed by CS polymer, surface-functionalized with PEG, and a cell-targeting peptide (CP15) is illustrated in Figure 14.

CS/PVA blends have greater thermal stability, unique morphology, and reduced solubility in acidic solutions [110]. Menazea et al. [111] prepared PVA/CS and PVA/CS doped with selenium NPs at different laser ablation times and studied the structural, optical, and antibacterial properties of the new composite blend. The results showed that doping of selenium NPs to PVA/CS increased the antibacterial activity in comparison to the pure PVA/CS blend.

##### Natural Polymers

To increase the specificity of CS as a drug carrier, it has been used along with another biopolymer (possessing specific biological properties) as a drug delivery device [18].

A chitosan-alginate (CS/ALG) polyionic complex is formed during ionic gelation via the ionic interactions between the amine group of CS and the carboxylic group of ALG. Since these interactions reduce the porosity of the complex it protects the encapsulated drug and slows the release more effectively than either CS or ALG alone. The high solubility of CS in low pH is reduced by the poor solubility of the ALG network at low pH, while ALG is stabilized at high pH by CS which is less soluble at high pH [112].

The other polymers to combine with CS, reported recently in the literature, are dextran [113], curdlan, carrageenan, caseinate [114], and pectin [115]. Curdlan possesses the ability to form high-set and low-set thermo-reversible gels at two distinct temperatures. Pectin is mucoadhesive at alkaline pH of the colon, passes intact through the upper gastrointestinal tract, and is degraded by colonic microflora so it is often used as a carrier for colon drug delivery. However, if used alone, pectin swells at alkaline conditions which may lead to premature release of a drug payload. When used in conjunction with other polymers such as CS, more stable matrices are formed for this drug delivery.

**Figure 15:**
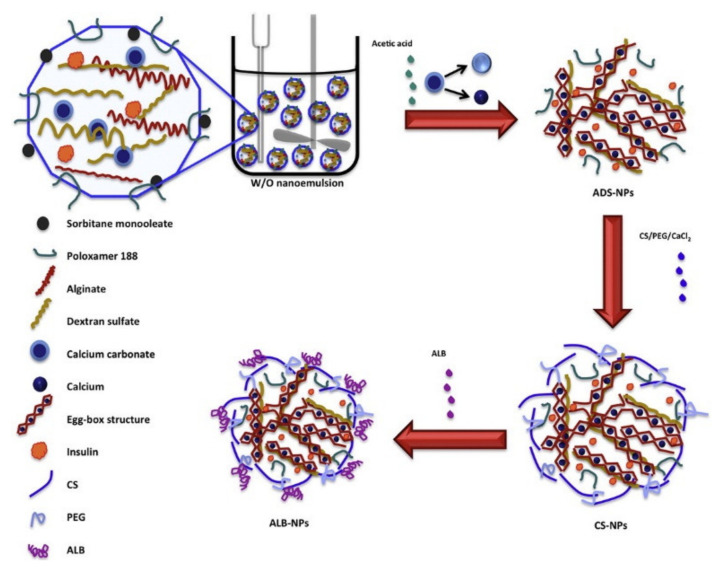
Schematic representation of the emulsification/internal gelation technique used to prepare the CS and albumin coated insulin-loaded alginate/dextran NPs [116]. Reproduced with permission from Elsevier (Copyright©2016).

The preparation of chitosan and albumin-coated insulin-loaded alginate/dextran NPs by Lopes et al. [116] is illustrated in Figure 15. The formation of the egg-box structure (calcium/alginate gel) was induced by the pH decrease in the water/oil (W/O) nanoemulsion, which enabled the controlled release of calcium. Insulin entrapment in the NPs matrix was reinforced by the presence of dextran sulfate. The dual coating was applied by polyelectrolyte complexation by dropwise addition of CS and albumin, sequentially.

#### 3.3.2. Chitosan-Inorganic Material Nanocomposite

Many nontoxic inorganic NPs are developed for drug delivery because of their excellent and well-controlled physical and chemical properties as the porosity of mesoporous silica, photosensitive fever, and luminescence of gold particles, magnetic responsiveness of iron oxide, the luminescence of carbon quantum dots. However, some disadvantages limit their applications in the biomedical field. Inorganic NPs may tend to aggregate under physiological conditions for their poor stability, they have potential side effects for non-targeting, and there are difficulties in their modification [11]. Therefore, various organic-inorganic hybrid nanocomposites have been emerging as a new and interesting tool for biomedical applications. Such NPs are mainly composed of an inorganic NP core and a multifunctional surface coating. CS is a good candidate for decorating the inorganic NPs because of its excellent properties aforementioned. The resulting hybrid materials exhibit remarkably improved properties as compared to their parent material [18].

##### Montmorillonite Clay (Mnt)

Mnt is a soft phyllosilicate group of minerals with a 2:1 layered structure consisted of silicate tetrahedral layers with alumina octahedral sheets sandwiched between them. The crystal lattice imperfection and isomorphous substitution create negative charge distribution with the plane counter-balanced by the adsorption of electropositive alkaline earth metal ions in the interlayer space. This is responsible for the activity and exchange reactions with organic compounds. Mnt has a large surface area which shows good cation exchange capacity, adsorption capacity, adhesive, and drug-carrying ability [18,117].

**Figure 16:**
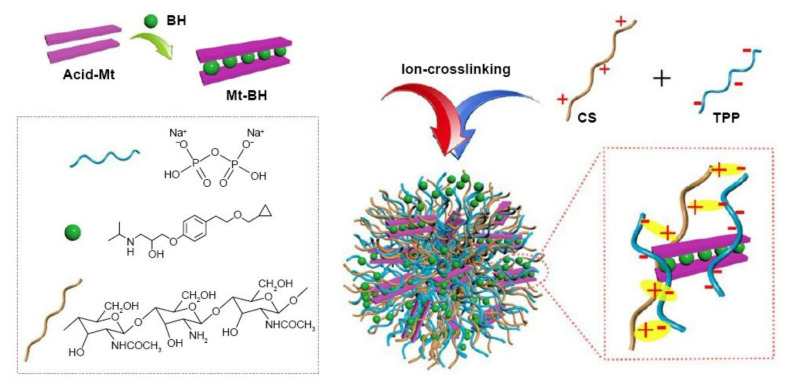
Schematic of the preparation process of betaxolol hydrochloride-Mnt/CS NPs using TPP as crosslinking agent [118]. Reproduced with permission from Dove Medical Press Limited (Copyright©2018).

A schematic of the preparation process of betaxolol hydrochloride-Mnt/CS NPs using TPP as a crosslinking agent for ocular delivery (treating of glaucoma) is shown in Figure 16 [118].

Preparation of CS/Mnt clay provides an alternative way of incorporating hydrophobic drugs into hydrophilic matrices. Luo et al. [119] developed a biocompatible CS/Mnt composite microsphere. It served as a carrier for loading and sustained release of the hydrophobic drug tanshinone IIA. The results of drug loading and in vitro release study of the tanshinone IIA loaded CS/Mnt composite microspheres showed that the incorporation of Mnt into CS matrix enhanced the drug encapsulation and retarded drug migration. The sample with a mass ratio of CS:Mnt (10:2) exhibited higher encapsulation efficiency (48.18% ± 2.54%) and slower continuous cumulative release of the drug in a phosphate buffer solution (pH 7.4). Cell viability studies by CCK-8 assay revealed that the microspheres provided no obvious cytotoxicity at dosages below 80 μg/mL, and the Mnt content had no significant effect on cell viability.

##### Mesoporous Silica Nanoparticles (MS NPs)

Among the nanocarriers, MS NPs are probably the most extensively studied. They are solid materials with well-ordered cylindrical pore structures. MS NPs have been utilized as delivery vehicles for controlled release due to their excellent characteristics including good biocompatibility, mesoporous structure, and chemical stability. Specifically, MS NPs offer appealing inherent properties including, controllable uniform particle size, high mechanical strength, and stability, as well as high drug loading capacities (they can load relatively larger amounts of bioactive molecules than other kinds of NPs). The fabrication of MS NPs is very simple and cost-effective. With appropriate modifications, these NPs can be taken up by nonphagocytic cells and can then release molecules encapsulated inside the MS NPs to the desired location. Many different types of functional chemical groups have been applied for the fabrication of MS NPs to achieve controlled drug release responding to environmental stimuli such as pH redox potential, enzyme, light, H_2_O_2_, and temperature [120,121,122].

**Figure 17:**
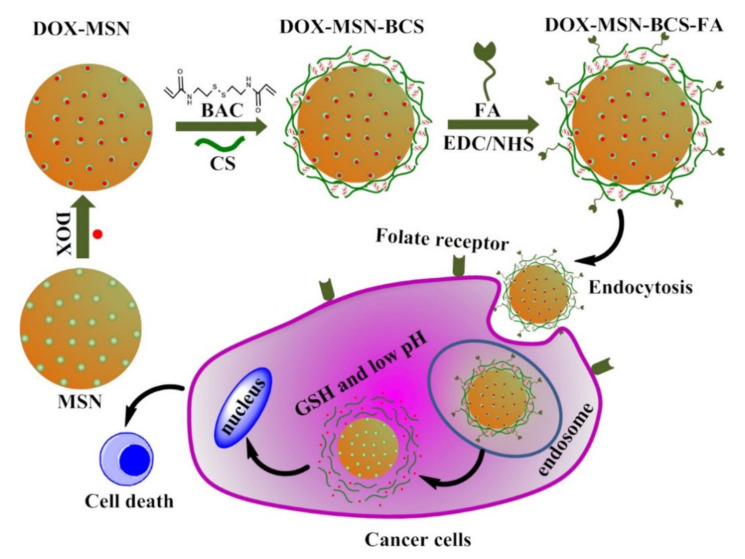
The synthesis procedure of DOX-MSN-BCS-FA and the extracellular and intracellular trafficking for DOX-MSN-BCS-FA to cancer cells [121]. Reproduced with permission from Elsevier (Copyright©2018).

However, one challenge in applying CS fabricated MS NPs is the premature leaking of the drug even under mild conditions, leading to reduced drug efficacy and nonspecific cytotoxicity. To overcome this issue Chen et al. [121] developed a multi-stimuli responsive surface on the MS NPs for an environmentally sensitive and site-specific drug delivery with reduced risk of premature drug leakage. A cross-linked CS was applied to form a thin film on the drug-loaded MS NPs resulting in a stimuli-sensitive regulating gate membrane. It was further conjugated with folate for a site-specific targeting toward cancer cells. The synthesis procedure of DOX-MSN-BCS-FA and the extracellular and intracellular trafficking for DOX-MSN-BCS-FA to cancer cells is illustrated in Figure 17. The CS film was very stable under neutral conditions and could effectively prevent drug leakage, but was sensitive to both pH and glutathione (GSH) stimulations to reach rapid drug release. Thus, the drug release could be triggered by changes in such factors that are common to cancer cells. However, the complete and accelerated release could only be achieved when triggered simultaneously by both acidic pH and GSH. Moreover, tests with HepG-2 cells confirmed that folate-receptor-mediated endocytosis successfully enhanced the cellular uptake of the NPs and antitumor activity toward cancer cells.

Liao et al. [123] attempted to enhance drug utilization and reduce its side effects by the strategy of “tumor-triggered targeting”. They fabricated a dual-pH-sensitive CS/MS NPs-based anticancer drug delivery system loaded with model drug doxorubicin hydrochloride (DOX). The MS NP was modified with the benzimidazole (Bz) group. Then chitosan-graft-β-cyclodextrin (CS-β-CD) was applied as the “gatekeeper” to cover the BzMS NPs through host-guest interaction between β-CD and Bz. After being coated with targeting peptide adamantane-glycine-arginine-glycine-aspartic acid-serine, methoxy poly(ethylene glycol) benzaldehyde was finally grafted on CS through the pH-sensitive benzoic imine bond. Due to the dynamic protection of PEG, the obtained carriers were “stealthy” at pH 7.4 but could reveal the shielded targeting peptide and the positive charge of CS in the weakly acidic environment achieved a “tumor-triggered targeting”. Inside cancer cells, the interaction between β-CD and Bz group could be destroyed due to the lower pH resulting in DOX release. Both in vitro and in vivo studies proved the DDS could targeting induce cancer cell apoptosis, inhibit tumor growth, and reduce the cytotoxicity of DOX against normal cells. It is expected that the developed CS/MS-based nanocomposite could be a potential choice for cancer therapy.

##### Magnetic Nanoparticles (Mag NPs)

**Figure 18:**
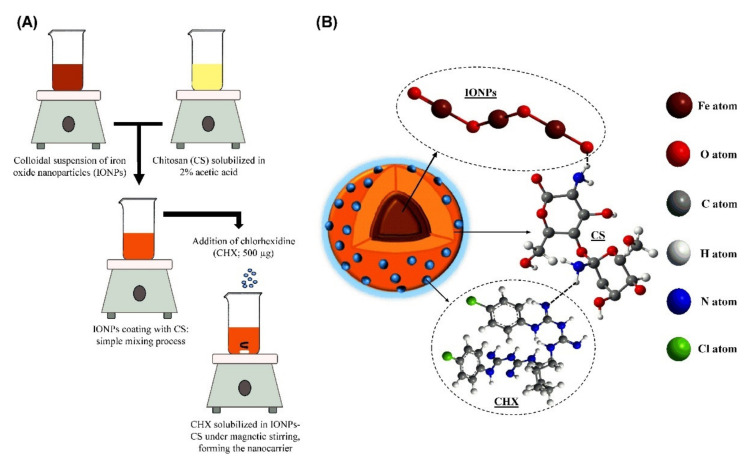
Schematic illustration of the steps involved in the preparation of the nanocarrier of chlorhexidine (**A**) and the plausible mechanisms of its formation (**B**). A colloidal suspension of iron oxide nanoparticles (IONPs) was mixed with chitosan (CS) previously solubilized in 2% acetic acid. After, chlorhexidine (CHX; 500 μg) was solubilized in CS-coated IONPs, and the suspension was stirred for 1 h to allow the nanocarrier formation. The amino group of CS interacts with both oxygen from IONPs and imine nitrogen group from CHX [126]. Reproduced with permission from Elsevier (Copyright©2020).

The use of magnetic nanocomposites in biomedical applications, particularly in cancer imaging and therapy, but also antimicrobial and others, is gaining immense interest. The illustrative scheme of the synthesis and structure ofchitosan-coated magnetic nanoparticles (CS Mag NPs) is shown in Figure 18. Mag NPs are promising materials for application in magnetic resonance imaging, targeted drug delivery, enzyme immobilization, and cancer therapies based on hyperthermia thanks to their biocompatibility, wide chemical affinity, and superparamagnetic properties [124]. Superparamagnetism refers to the ability of Mag NPs to gain magnetism in the presence of a magnetic field and lose magnetism when the magnetic field is eliminated. It means the magnetic nanocarriers must be responsive to the magnetic gradient. This behavior of Mag NPs is beneficial for targeted drug delivery when a chemotherapeutic drug can reach the targeting disease site selectively by an application of an external magnetic field [11,125]. Except for the magnetic field, the drug nanocarriers can respond to other external stimuli (such as pH, ultrasound, temperature, etc.). The change in their physiological properties leads to the release of a specific concentration of bioactive agents at the affected area or organ. Through this process, the effectiveness and specificity of the drug could enhance and leads to lower systemic toxicity. Another important feature of Mag NPs is that they can induce malignant tissues death by magnetic fluid hyperthermia. The particles produce heat in the vicinity of alternating electromagnetic fields. The theory behind magnetic hyperthermia is that cancerous cells are more susceptible to the external stress of heat than normal cells due to low oxygen and nutrients and normal acid concentrations [18,125].

Khmara et al. [127] synthesized magnetite nanoparticles (Mag NPs) by coprecipitation method followed by their coating with chitosan. The specific absorption rate (SAR) of both uncoated Mag NPs and modified CS/Mag NPs increased with the applied magnetic field up to ~7.9 kA·m^−1^. Moreover, the SAR values of CS/Mag NPs were higher than those of Mag NPs demonstrating the possibility of their utilization as nanoheaters for hyperthermic treatment. It was observed that the CS/Mag NPs can destroy α-lactalbumin amyloid fibrils in a concentration-dependent manner. Drug delivery systems prepared with these nanostructures can overcome biological barriers. However, one of the main challenges in the use of these nanosystems is their internalization by macrophages.

There is still a lack of knowledge of interactions between Mag NPs covered with the bioactive polymers and biological cells. To fulfill this gap, Piosik et al. [124] investigated interactions of newly synthetized magnetite (Fe_3_O_4_) nanoparticles functionalized with aminated chitosan (Mag/aminated CS) and a model biological membrane made of dipalmitoyl-phosphatidylcholine (DPPC) using a Langmuir technique. The DPPC films revealed the strong influence of the Mag/aminated CS NPs on the stability, phase state, and structure of the phospholipid membrane. The studies on the adsorption/incorporation process of the Mag/aminated CS NPs showed that they can adsorb/incorporate into the DPPC model membrane at the surface pressure corresponding to this present in the cellular membrane under the biological conditions (35 mN·m^−1^). The number of the adsorbed/incorporated Mag/aminated CS NPs can be regulated by the NP concentration in the neighborhood of the DPPC model membrane even at a high surface pressure of 35 mN·m^−1^.

Bandeira et al. [128] prepared and characterized CS NPs incorporating maghemite nanoparticles (Mag NPs) and investigated their intracellular tracking in RAW 264.7 macrophages in vitro. Mag NPs were encapsulated within CS NPs by ionotropic gelation. The images from transmission electron microscopy were used to investigate the intracellular penetration of conjugated NPs by macrophages using different times. Data suggests that the Mag NPs are suitable to act as a contrast agent to investigate the cellular internalization of the CS NPs.

##### Graphene Oxide (GO) Modified Nanocomposite

Graphene oxide (GO) is a type of two-dimensional monoatomic carbon allotrope—plate-like structure derived from graphite or graphene. GO contains many functional groups, a large surface area on both sides of the GO sheets, and a high loading capacity of aromatic molecules and nucleobases through π-π stacking and hydrogen bonding interactions. GO NPs are designed to increase the concentration of drugs in tumor sites (enhanced permeability and retention effect, EPR), longer circulation time in blood, high drug loading capacity, targeting effect, and controlled drug release. This makes the GO an efficient drug carrier and has been explored in drug delivery applications extensively. The oxidized form of graphene contains various functional groups such as hydroxyl, epoxy, and carboxyl groups, etc. The epoxy group can be easily modified with amino groups by reducing the bandgap and thereby increasing the adsorption of drug molecules. To enhance the stability of GO NPs in physiological environments, they have been functionalized with hydrophilic and biocompatible polymers (e.g., polysaccharides), which also reduce toxicity and increase solubility and biocompatibility of GO. So that the graphene-based polysaccharides should be water-dispersible, non-toxic, biodegradable, and biocompatible for biomedical applications [40,129].

**Figure 19:**
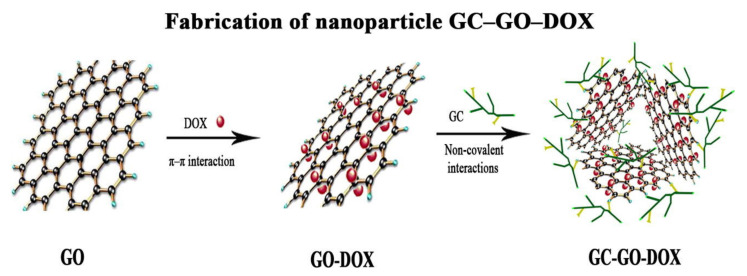
Schematic representation of the synthesis of galactosylated CS and the fabrication of GC–GO–DOX NPs [40]. Reproduced with permission from Elsevier (Copyright©2018).

Wang et al. [40] investigated a novel drug delivery system comprising nanoparticles based on galactosylated chitosan (GCS), graphene oxide (GO), and doxorubicin (DOX-GCS/GO NPs) for the treatment of cancer. Schematic representation of the synthesis of galactosylated chitosan (GS) and the fabrication of DOX loaded GC–GO NPs, as an example of CS/GO-based nanocomposites, is represented in Figure 19. The NPs remained stable under physiological conditions, and the drug was released in a low pH environment (i.e., a tumor environment) and was pH-responsive. Cell uptake experiments and a cell proliferation analysis demonstrated that the NPs had higher cytotoxicity for HepG2 and SMMC-7721 cells than DOX-CS/GO NPs. DOX-GCS/GO NPs exhibited a higher fluorescence intensity in tumor cells than DOX-CS/GO NPs. In vivo anti-tumor experiments demonstrated that the DOX-GCS/GO NPs inhibit tumors better than the DOX-CS/GO NPs.

Rebekah et al. [130] prepared Mag NPs decorated with graphene oxide chitosan composite (GO/CS/Mag NPs) as a nanocarrier for protein delivery to solve the problem of instability and less life span of proteins. They used bovine serum albumin (BSA) as a model protein and studied its stability and activity. SDS page analysis showed no remarkable change after exposing BSA-GO/Mag NPs and BSA-GO/CS/Mag NPs in trypsin after a time duration of 30 min and 3 h, respectively. The GO/CS/Mag NPs exhibited better drug loading and releasing profile when compared with GO/Mag NPs composite. This nanocarrier protected the protein from enzymatic cleavage more efficiently. Hence, the GO/CS/Mag NPs composite was a favorable nanocarrier with potentialities for clinical application.

There is currently a great demand, especially in cancer treatment, for transformative theranostic technologies combining imaging with drug delivery. Baktash et al. [131] designed and optimized a hybrid theranostic nano-system combining the imaging capabilities of Mag NPs with the great potential of CS-grafted graphene oxide (GO/CS) as a pH-sensitive smart nanocarrier, using different molecular weights and concentrations of CS. The loading and release behavior, biocompatibility, and magnetic properties of the GO/CS/Mag NPs were evaluated using a model drug doxorubicin (DOX). Increased pH resulted in a reduction in the rate of DOX release, suggesting the formation of hydrogen bonds and the physical prevention of collapsed CS chains. In addition, a decrease in CS molecular weight and an increase in concentration reduced the DOX loading by around 24% yet a decrease in molecular weight increased the released amount by more than 200%. This has been related to fewer hydrogen binding and more contribution of π-π stacking in DOX-CS interactions. Cytotoxicity assays with healthy L929 cell lines revealed high biocompatibility of the GO/CS/Mag NPs, suggesting CS prevents GO contact with the cell membrane. Results showed that the hybrid GO/CS/Mag NPs with high molecular weight CS at a 6.0 g/dL concentration showed optimal properties for theranostic applications.

##### Gold Nanoparticles (Au NPs)

More recently, gold nanoparticles (Au NPs) have earned widespread application in drug delivery studies due to their unique properties, including biocompatibility, low toxicity, ease of production, and the large surface-to-volume ratio [132].

**Figure 20:**
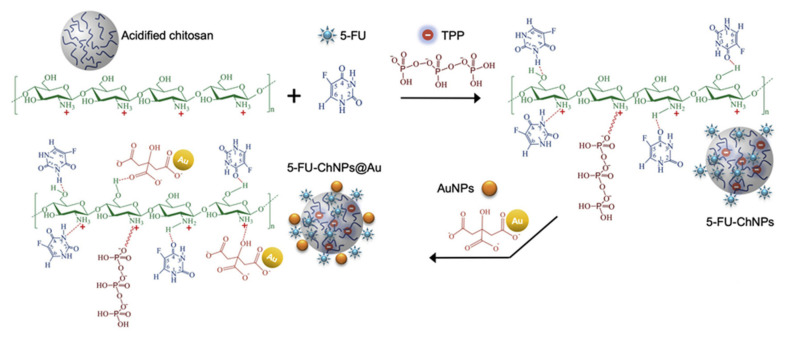
Fabrication steps of 5-FU-CS NPs, and 5-FU-CS NPs/Au NPs. Electrostatic interaction (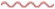) between positively amino groups of CS and negatively charged TPP groups is built via the ionic gelation method. The hydrogen bond interaction (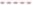) occurs between the hydrogen atom and a highly electronegative atom as O or N atom [133]. Reproduced with permission from Elsevier (Copyright©2021).

Gold nanospheres, as a kind of photo-absorbing agent, can generate heat from optical light energy for photothermal antitumor therapy. Salem et al. [133] prepared a ternary system composed of optimally prepared CS NPs efficiently encapsulating 5-fluorouracil (5-FU) and loaded by Au NPs. They employed it for a synergistic chemo-photothermal therapy. Fabrication steps of 5-FU-CS NPs, and 5-FU-CS NPs/Au NPs preparation are demonstrated in Figure 20. The composed 5-FU-CS/Au NPs exhibited excellent biocompatibility, stability, and killing efficiency of tumor cells. Moreover, its strong absorbance in the visible region promoted the fast release of the drug under laser exposure. The synergistic effect of 5-FU-CS/Au NPs was demonstrated in comparison to the system of 5-FU-CS NPs. Cytotoxicity experiments towards hepatocellular carcinoma cells (HepG2) revealed that the combined chemo-photothermal therapy of the 5-FU-CS/Au NPs featured a highly synergistic manner. These findings offer the 5-FU-CS/Au NPs as a highly potential chemo-photothermal therapeutic platform.

Au NPs are also promising in the inhibition of bacterial pathogens. They cause bacterial cell death through cellular penetration and mitochondrial membrane damage. Saravanakumar et al. [134] synthesized CS/Au NPs and tested them for their bioactivities. The CS/Au NPs exhibited higher antioxidant, antibacterial, and diabetics-related enzyme inhibitory activities than CS NPs. A 50% of the LN229 cell death occurred through the treatment with 218.75 μg·mL^−1^ or 500 μg·mL^−1^ of CS/Au NPs or CS NPs, respectively. The inhibitory concentration (IC50) of CS/Au NPs showed higher cytotoxicity in LN229 via cellular damage, mitochondrial reactive oxygen species (ROS) balancing, and arresting about 42.33% cells in the G1 phase. These results proved the multifunctional bioactive properties of the CS/Au NPs including antioxidant, antibacterial, antidiabetics, and anticancer activities and it deserves further molecular elucidation.

To investigate the synergistic effect of two drugs (DOX and aptamer against Forkhead box M1 (FOXM1 Apt)) co-delivered in CS/Au NPs on cancer cells, Khademi et al. [132] firstly prepared the vehicle composed of CS/Au NPs conjugate. Then nucleolin aptamer (AS1411) and FOXM1 Apt were loaded onto the CS/Au NPs and formed Aptamers (Apts)-CS/Au NPs. Subsequently, DOX was added to the Apts-CS/Au NPs to obtain the DOX-Apts-CS/Au NPs complex for synergistic treatment of tumor. The data of flow cytometry analysis and fluorescence imaging displayed that the complex was effectively internalized into target cells (A549 and 4T1 cells, nucleolin+) but not into CHO (Chinese hamster ovary) cells as non-target cells. The results of the MTT assay (a colorimetric assay for assessing cell metabolic activity) showed that the complex significantly increased cell mortality in the 4T1 and A549 cells compared to the CHO cells treated with the complex. The in vivo studies demonstrated that the DOX-Apts-CS/Au NPs complex exhibited a more pronounced tumor inhibitory effect and less distribution in other organs compared to free DOX.

Lopez-Pérez et al. [135] developed a new method for the covalent immobilization of biomolecules on the surface of bare Au NPs by using crosslinking agents in one step. CS has been used as a target molecule to probe the viability of the proposed methodology. Click chemistry, based on biocompatible reactions and coupling with 1-Ethyl-3-(3-dimethylamino propyl) carbodiimide hydrochloride and N-hydroxysuccinimide allowed to analyze the covalent interactions between the metal NPs and the biopolymer. The obtained results indicated that covalent interactions can be increased up to 25% concerning total system interactions, which are mostly electrostatic. The proposed strategy opens up a new pathway for biomedical applications because the control of the chemical linkage can be directly performed on the NP surface without using any molecular intermediate, which may improve the encapsulation efficiency of drug delivery therapies.

##### Zinc Oxide Nanoparticles (ZnO NPs)

**Figure 21:**
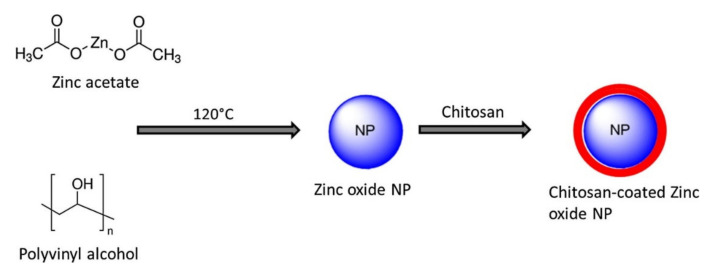
Schematic presentation of preparation chitosan-coated zinc oxide nanoparticles [136]. Reproduced with permission from Elsevier (Copyright©2018).

Inherent antimicrobial and selective anticancer activities of zinc oxide nanoparticles (ZnO NPs) together with their other biomedical potentials (including imaging and photo dynamic therapy (PDT)) and unique properties such as appropriate biocompatibility and easy synthesis make them promising applicants as antimicrobial, anticancer, antioxidant and antidiabetic agents, in tissue engineering, bioimaging [136]. Schematic presentation of preparation chitosan-coated ZnO nanoparticles is illustrated in Figure 21.

ZnO NPs have been revealed to have a wide range of antibacterial effects against both Gram-positive and Gram-negative bacteria. Several researchers have also studied the preferential anticancer activities of various ZnO nanostructures. These activities are mainly attributed to the ability of ZnO NPs to generate reactive oxygen species (ROS), zinc-mediated protein activity disequilibrium, and their capability to release zinc ions. A suggested mechanism behind the preferential cytotoxicity of ZnO NPs is the greater ROS generation in rapidly proliferating cells including cancer cells. ZnO NPs cause oxidative stress and subsequent cell damage within cancer cells due to their rapid dissolution into Zn2+ ions at slightly acidic pH, thereby ZnO NPs show pH-responsive cytotoxicity. The generation of more ROS by ZnO NPs, resulting in huge oxidative stress that can finally kill the cells [137].

George et al. [138] conjugated L-Cysteine (Cys) with CS through the amidation reaction followed by the incorporation of phyto-synthesized ZnO NPs and created a nanohybrid hydrogel carrier for the enhanced therapeutic delivery of naringenin (NRG). The Cys conjugation with CS stabilized the hydrogel and enabled a sustained release of NRG drug. The antimicrobial activity was studied against the Staphylococcus aureus and *Trichophyton rubrum* strains. Biocompatibility assay of the materials with L929 cells revealed significant cell viability. The developed nanohybrid hydrogel containing NRG exhibited a two-fold increase in cytotoxicity towards A431 human skin carcinoma cells compared to NRG delivery without the carrier.

Ghaffari et al. [139] prepared ZnO NPs functionalized by N-succinyl chitosan (NSC) as a pH-sensitive delivery to enhance the therapeutic potential of curcumin (CUR). The spherical-like CUR-conjugated system (CUR-NSC/ZnO NPs) with the average particle size of 40 nm provided significantly enhanced water dispersibility versus free CUR. The in vitro release study of CUR showed a pH-sensitive release profile, which enabled drug delivery to tumors and infection sites. MTT and Annexin-V FITC/PI assays revealed the superior anticancer activity of the CUR-NSC/ZnO NPs compared to free CUR against breast cancer cells (MDA-MB-231) by inducing the apoptotic response with no cytotoxic effects on HEK293 normal cells. Moreover, CUR conjugation to the system notably dropped the minimum inhibition concentration MIC (25 to 50-fold) and minimum bactericidal concentration MBC values (10 to 40-fold) against *S. aureus* and *E. coli*. The features qualify the formulation for anticancer and antimicrobial applications in the future.

##### Carbon Quantum Dots (CQDs)

Carbon dots are NPs of carbon and quantum dots are NPs of any kind. Carbon quantum dots (CQDs) or simply carbon dots (CDs) have gained considerable importance as a versatile material for multiple applications. These quasi-spherical particles, with an average diameter of below 10 nm, usually possess an sp2 conjugated core with various oxygen-containing functionalities such as carboxyl, hydroxyl, aldehyde groups, etc. CQDs may be termed as NPs with a diameter less than 10 nm. Owing to their hydrophilic nature, fair water solubility, low cytotoxicity, fair cell permeability, good biocompatibility, ease of functionalization, and production of fluorescence on UV exposure, CQDs have found several biomedical applications like bio-imaging, targeted drug delivery, wound dressings, cancer theranostics, screening the purine metabolic disorders in human fluid, etc. 

CQD as novel nanomaterials have been widely studied for photothermal therapy and NIR fluorescence imaging because they combine unique optical properties with good biocompatibility, small size, low cost, NIR absorption, and emission nature [11]. Fluorescent (luminescent) CQDs can be used to track biological processes inside cells. They are less toxic than similar alternatives and therefore are more suitable for use in live biological systems. The main reason for their application in cell imaging is that they have a size below 10 nm, which makes them permeable through the cell wall [140].

**Figure 22:**
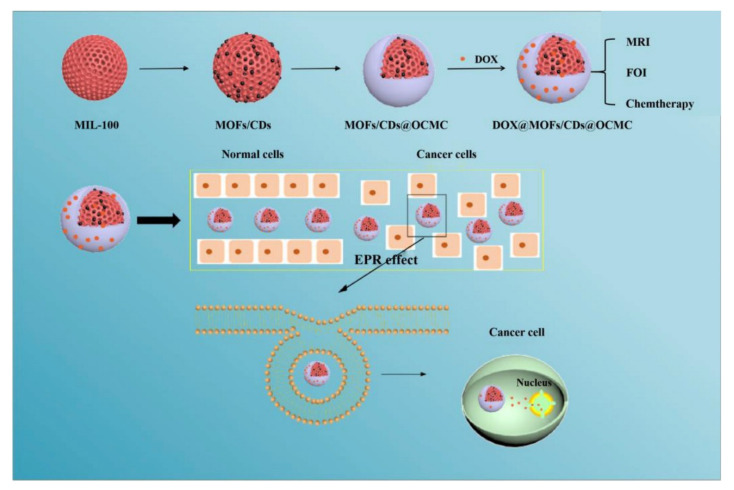
Schematic illustrated the preparation of the MOFs/CDs/OCMC nanocomposites and their application as drug carriers of DOX for the treatment of cancer [141]. Reproduced with permission from Elsevier (Copyright©2020).

To demonstrate their optical imaging properties, Lin et al. [141] encapsulated CQDs into novel core-shell Metal Organic Frameworks (MOFs). The composite was further coated with O-carboxymethyl chitosan (OCMC) to form OCMC/MOFs/CQDs to improve biocompatibility and endow pH-responsive property. Schematic illustration of the preparation of the MOFs/CDs/OCMC nanocomposites and their application as drug carriers of DOX for the treatment of cancer is shown in Figure 22. The system was validated for in vitro diagnosis and treatment of cancer. The composite exhibited excellent performance in fluorescence imaging (FOI) and magnetic resonance imaging (MRI), which proved the OCMC/MOFs/CQDs could be used for FOI/MRI dual-mode imaging. Cytotoxicity assays indicated the OCMC/MOFs/CQDs particles were highly biocompatible and suitable to be used to transport drugs in human bodies. The model drug doxorubicin (DOX) was released rapidly at pH 3.8, but the rate and extent of release were greatly attenuated at pH 7.4. Hence, the particles demonstrated an excellent pH-triggered drug release performance.

Sheng et al. [142] prepared graphene quantum dots (GQDs) by the pyrolysis of citric acid (CA). They used them for the loading of hydrophilic cytarabine (Cyt), an anti-cancer drug, and then wrapped them with CS gels for the encapsulation of the loaded Cyt. The fluorescent stability of GQDs was significantly enhanced in the presence of CS, which might be attributed to the inhibited agglomeration of GQDs by the CS gels. In addition, the burst release of Cyt from the developed carrier was also effectively relieved by the CS coating. Since the incorporation of Cyt into GQDs was achieved by amidation reaction, the delivery of Cyt from the carrier was pH-sensitive due to the hydrolysis of the amido-linkage between GQDs and Cyt in an acidic medium.

##### Carbon Nanotubes (CNTs) Composite

CNTs are allotropes of carbon, cylindrical nanostructure consisting of sp2 hybridized carbon atom in a hexagonal arrangement. They have excellent electrical property, regular pore structure, high specific area, and therefore are suitable for controlled release. In their structure the polymer offers biocompatibility and biodegradability, CNTs provide stability, cellular uptake, magnetic and electromagnetic behavior. The major concern of these nanocomposites is cytotoxicity [18].

Dong et al. [143] developed a sustained and controlled drug delivery system based on a polycaprolactone-polyethyleneglycol-polycaprolactone (PCL/PEG/PCL) thermosensitive hydrogel combined with chitosan-multi-walled carbon nanotubes (CS/MWCNTs) for near-infrared light-triggered drug delivery. CNTs that incorporate the hydrogel can enhance the sustained effect of drug delivery by a dual-stage release and allow drug delivery by controlling light irradiation. The controlled drug delivery of DOX was tracked in real-time by fluorescence imaging in vivo based on the fluorescence ability of the drug, using nude mice as models. The results suggest that the photothermal effect of the CNTs can disrupt the structure of the hydrogel with a gel–sol transition, triggering the release of the drug from the sustained drug delivery system by NIR irradiation while responding on-demand.

**Figure 23:**
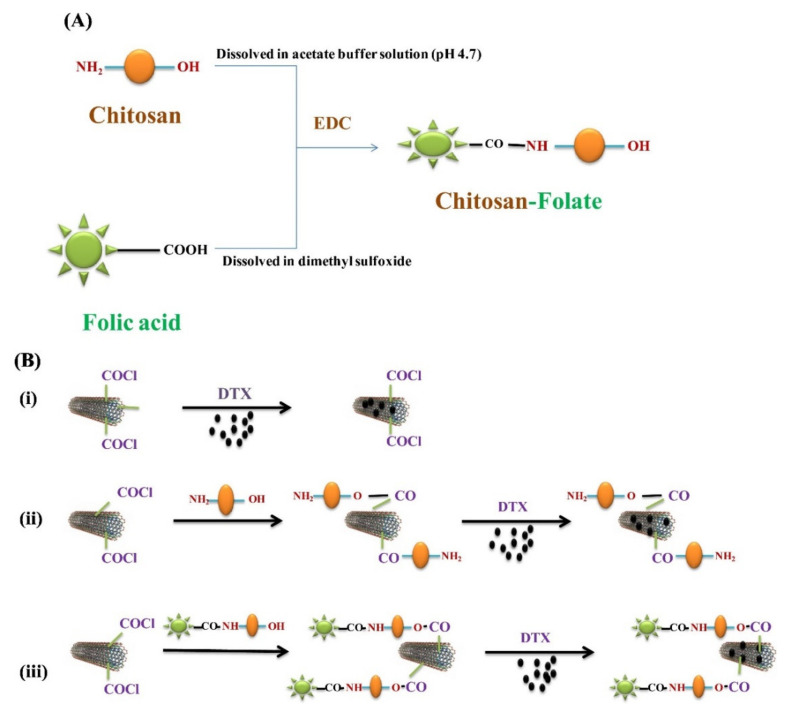
(**A**) Schematic diagram for synthesis of CS-folate (CS-FA) conjugate and (**B**) preparations of (**i**) DTX-AcMWCNT (non-conjugated) (**ii**), DTX-CS/AcMWCNT (CS conjugated), and (**iii**) DTX-CS-FA/AcMWCNT (CS-FA conjugated) [144]. Reproduced with permission from Elsevier (Copyright©2017).

Singh et al. [144] formulated chitosan-folate conjugated multi-walled carbon nanotubes (CS/FA/MWCNTs) for the lung cancer targeted delivery of docetaxel. A schematic diagram for the synthesis of CS-FA/MWCNTs is illustrated in Figure 23. The cellular internalization study has shown that the CS/FA/MWCNTs could be easily internalized into the lung cancer cells through a folate receptor-mediated endocytic pathway. The IC50 values indicated that the CS/FA/MWCNTs could be 89-fold more effective than Docel™ (marketed docetaxel formulation) in human lung cancer cells (A549 cells).

##### Other Inorganic Materials Combined with CS

Several other inorganic materials have been developed recently, including metal–organic framework (MOFs) and layered double hydroxides (LDHs) combined with CS for the construction of DDSs [11].

**Figure 24:**
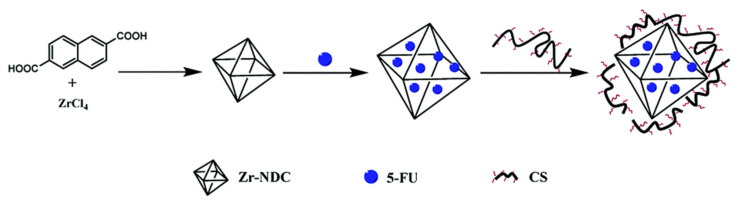
Synthesis of CS/MOF loaded with 5-FU [146]. Reproduced with permission from Elsevier (Copyright©2020).

MOFs are highly tunable hybrid materials; consist of metal ions linked together by organic bridging ligands. MOFs have been used as an efficient drug delivery carrier because of their biodegradability, low toxicity, and structural integrity upon loading and functionalizing process. MOFs can be used to improve antibiotic therapy and fight multi-drug resistance. Synthesis of CS/MOF loaded with 5-FU is demonstrated in Figure 24. Ghaffar et al. [145] synthesized CS-coated MOFs with enhanced contact with S. aureus cell surface. The novel system was studied for its efficacy on resistant *S. aureus*. The results revealed that Vancomycin bactericidal activity significantly increased upon loading into the CS-coated MOFs and caused increased inhibition of resistant S. aureus. Atomic force microscopy (AFM) analysis of *S. aureus* strains revealed complete distortion of morphology by treating with the CS modified drug-loaded MOFs. The findings of this study suggest the potential of CS-coated MOFs for reversing bacterial resistance against Vancomycin and provide new perspectives for improved antibiotic therapy of infections associated with multi-drug resistance.

**Figure 25:**
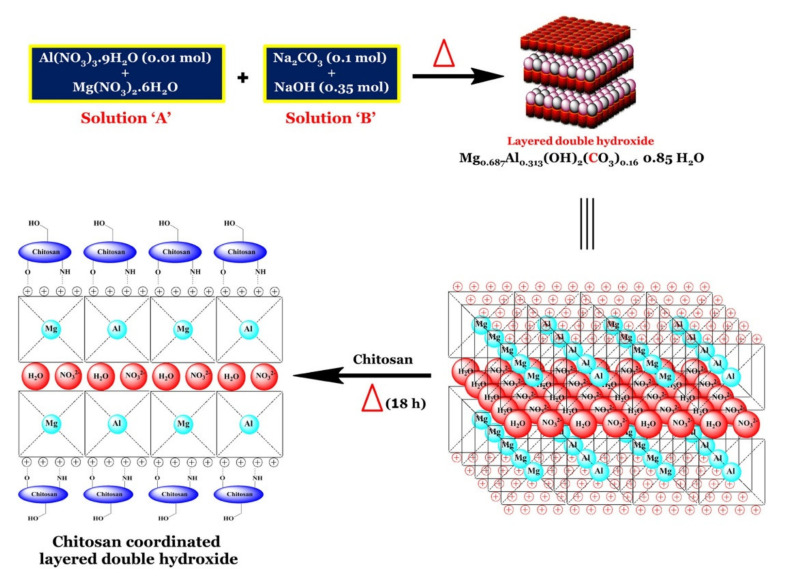
Preparation of CS/Mg-Al layered double hydroxide composite [149]. Reproduced with permission from Elsevier (Copyright©2017).

LDHs have always been useful inorganic materials in the pharmaceutical field because of their small particle size, large specific surface area, good biocompatibility, and large surface positive charge density. LDHs can form organic-inorganic hybrid nanocomposites with multifunction properties by hybridizing with functional organic polymers such as CS [147]. The basic scheme for the preparation of CS coordinated LDHs is shown in Figure 25. Wang et al. [148] designed a targeted hybrid nanocomposite based on LDHs and functional carboxymethyl chitosan (CMC) derivatives for the efficient delivery of dexamethasone disodium phosphate (DEXP). A special substrate of peptide transporter-1 (PepT-1) and glutathione (GSH) was modified on CMC. CMC/GSH/GS (glycylsarcosine)/LDHs and CMC/GSH/VV (valyl-valine)/LDHs hybrid nanocomposites were prepared and structurally confirmed. More details about the application of this nanocomposite in drug delivery are in Section 3.4.2.

#### 3.3.3. Stimuli Responsive Chitosan, Chitosan Nanoparticles, and Nanocomposites

Due to disparity in the environment (pH, enzyme concentration, etc.) around diseased or injured/pathologic areas compared to healthy ones, stimuli-responsive materials for drug delivery have gained considerable attention recently, as it has been reviewed by Sabourian et al. [150].

There are two types of stimuli: (i) internal that include glutathione (GSH), reactive oxygen species (ROS), enzymes, pH, and temperature, and (ii) external such as ultrasound, light, magnetic/electrical fields, and mechanical stimulus. Among a variety of biomaterials that can respond to these stimuli, modified CS as a biocompatible polymer with specific functional groups, provides an advantageous platform, as its solubility, hydrophilicity, biodegradability, and chemical behavior can be easily modified concerning conditions of the biological environment. The specific environmental stimulants at diseased sites can be used to initiate and/or control drug release. Modified CS can respond to these pH changes by swelling, shrinking, degrading, protonation, and deprotonation of functional groups, and thus release an encapsulated drug in a controlled manner. Pathological sites such as ischemia, infections, inflammation, or cancerous tumor have different pH profiles than the normal tissue [129]. In addition, functionalized CS can respond to the body temperature under normal and disease conditions (37 °C to 37.5–41 °C), due to changing hydrophilic and hydrophobic interactions between stimuli-responsive conjugated species and CS chains, below and above its lower critical solution temperature (LCST) [151].

*pH and temperature-responsive NPs*: Liu et al. [152] developed a dual (pH and temperature) responsive bio-nanomaterial to improve the efficiency of an anesthetic drug delivery system. The surface engineering of ZnFe_2_O_4_ NPs was performed by coating with chitosan using a simple precipitation method. Then, a multi-active anesthetic drug (lidocaine) was loaded into nano-ferrite to form a drug delivery vehicle. XRD analysis proved the face center cubic structure of zinc nanoferrite. The sustained delivery of lidocaine from the CS coated nanoferrite (CS/ZnFe_2_O_4_) was stimulated by pH and temperature-responsive characteristics of vesicles. The drug-loaded CS/ZnFe_2_O4 particles exhibited high biocompatibility and sustained drug release in the physiological pH environment (4.8, 5.5 vs. 7.4) and temperature-responsive (25 vs. 37 °C) of normal tissues.

*Redox/ROS responsive NPs:* High concentration of ROS in cancer cells represents another example of stimulus in the body, as well as at sites including neurological disorders, cardiovascular diseases, sensory impairments, inflammation, infectious and fibrotic diseases. Modification of CS by ROS-responsive entities has been shown to accelerate ROS deactivation, while there was no significant change in ROS content with naked CS. GSH is produced intracellularly and is required for cell life. However, in some diseases such as cancer, it can be increasingly generated in the cells. The enhanced concentration has been used as a stimulus for drug delivery by chemically linking molecules with disulfide bonds, and thus develops easily manipulated GSH-responsive structures. Very recently, Daund et al. [153] published a review on ROS responsive NPs for smart drug delivery. Jiao et al. [154] used hollow mesoporous silica nanoparticles (MS NPs) as the drug vehicle to develop the redox and pH dual stimuli-responsive delivery system, in which the chitosan was grafted on the surface of MS NPs via the cleavable disulfide bonds. The PEG was further grafted on the surface of CS to increase the stability and biocompatibility under physiological conditions. In vitro release results indicated that DOX was dramatically blocked within the mesopores of NPs in a pH 7.4 PBS without the addition of GSH. However, the release rate of DOX was markedly increased after the addition of 10 mM GSH or in a pH 5.0 release medium. Lin et al. [155] presented redox-responsive nanocarriers for drug (DOX) and gene (p53 gene) co-delivery based on CS derivatives modified MS NPs. A dendronized CS derivative as a “gatekeeper” to control the release of the drug was used to modify MS NPs via a disulfide linker and improve the gene transfection for potential cancer therapy. Stimulus-induced release of the DOX was studied in the presence of glutathione (GSH), which showed that the polymer shell was shed and accelerated the release of embedded drugs inside the tumor cells under a GSH-rich environment. 

*Enzyme-responsive NPs:* Enzyme-responsive systems have gained tremendous attention to develop smart biocarriers for drug delivery since, compared with other stimuli-responsive systems, they benefit from both high sensitivity and precise selectivity in the enzyme-overexpressed tissues or cells. Enzymes are responsible for converting the assembled amphiphilic biocarriers into non-assembled non-amphiphilic structures so that the drug can be released. Rastegari et al. [156] introduced the two glucose-based smart tumor-targeted drug delivery systems for enzyme-sensitive release strategy. Magnetic nanoparticles (Mag NPs) were grafted with carboxymethyl chitosan (CMC) or β-cyclodextrin (β-CD) as carriers. Prodigiosin was used as the model anti-tumor drug, targeting aggressive tumor cells. Measurement of in vitro release of prodigiosin from the loaded nanocarriers in the presence of the hydrolytic enzymes, alpha-amylase, and chitosanase showed that 58.1 and 44.6% of the drug were released after one hour of incubation. The results of cytotoxicity studies suggested that the CS/Mag nanocomposites have higher potency and are more suited to target the prodigiosin toxicity effect on cancerous cells than β-CD/Mag nanocomposites.

*External stimuli NPs:* External stimuli such as light [133], ultrasound [157], heat [39], and magnetic/electrical fields [152] affect the conformation or configuration of CS in the diseased tissue. These stimuli can be applied to improve cargo release into the diseased area to treat cancer, heart attack, and neurodegenerative diseases including Alzheimer’s and Parkinson’s. 

In a biological system, NIR (near infra-red) has a deep penetrating ability without harmful side effects. NIR responsive behavior of CuS is an ideal photothermal trigger to facilitate the release of the encapsulated drug. Mathew et al. [158] examined the ability of the drug to be released from the nanocomposite on the application of NIR. They synthesized nanospheres of covellite copper sulfide (CuS) with chitosan as a base. Dopamine was conjugated to the CuS/CS nano-drug carrier system. The dopamine-CuS/CS nanocomposite was introduced into a dialysis bag and the release of the drug through NIR triggering from the carrier was studied. Cytotoxicity/cell viability assay showed that the viability of the cells and their cell cycle were not affected indicating the carrier, as well as the nanocomposite, are non-toxic. Thus, this photo-controlled technique showed a potential to control and manage the targeted release of encapsulated drugs that are non-toxic especially in the context of neurodegenerative diseases.

As for a photo-controlled DDS used for cancer treatment, Bhatta et al. [159] have reported the physico-chemical, photophysical, and morphological properties of chlorin e6 (Ce6) decorated doxorubicin (DOX) encapsulated chitosan/TPP NPs prepared by an ionotropic gelation method. The NPs showed high encapsulation efficiency towards DOX as well as pH-controlled release. This had significant anti-proliferative activity against MCF-7 breast cancer cells after irradiation at (NIR) ranges. This could have potential applications in photo-controlled smart DOX delivery systems for cancer treatment.

### 3.4. Chitosan Nanoparticles in Drug Delivery

CS NPs have numerous applications in the pharmaceutical, biological, and medical discipline including their implementations in drug delivery [52]. The main advantages of CS NPs in controlled release system include (i) improved stability of the drug, (ii) reduced side effect of the drug, (iii) reduced doses, (iv) increased convenience, (v) increased efficacy of drug, (vi) improved bioavailability of the drug, (vii) reduced overall cost, and (viii) better patient compliance [9]. 

In the following subsections, very recent application examples of CS-NPs are discussed as the drug delivery systems and divided according to the route of delivery. From this point of view, the reviewed delivery systems are divided as follows: Section 3.4.1 Oral drug delivery (including Antidiabetic drugs, Anticancer drugs, Antihypertensive drugs, Antioxidants, Anti-inflammatory drugs, Oral vaccines, and Other drugs), Section 3.4.2 Ocular drug delivery, Section 3.4.3 Nasal drug delivery (including Topical nasal delivery, Systemic nasal delivery, and Nose to brain delivery), Section 3.4.4 Pulmonary drug delivery, Section 3.4.5 Buccal drug delivery, Section 3.4.6 Periodontal drug delivery, Section 3.4.7 Dermal and transdermal drug delivery, Section 3.4.8 Wound healing, and Section 3.4.9 Vaginal drug delivery. Section 3.4.10 Vaccine delivery and Section 3.4.11 Gene delivery are stated separately because of the specificity of delivered biomolecules or multiple routes of possible delivery (including i.v., oral, nasal, etc.). The application examples are listed in Table 3 according to the (i) type of CS NP, (ii) method of CS NP preparation, (iii) formulated drug, and (iv) in vitro and in vivo tests for biological activity and drug release.

#### 3.4.1. Oral Drug Delivery

Oral drug administration remained the preferred route of drug uptake due to the safety, good patient compliance, and non-invasive administration (preventing tissue damage). However, this method of administration is slow, and some drugs may be damaged by gastric fluids, or they can irritate the gastrointestinal tract, GIT [11]. Many biological therapeutics were rarely used for oral drug delivery due to low water solubility and poor oral bioavailability. An ideal carrier should form stable complexes with active substances in GIT, protecting the active substances from degradation and delivering it to target cells or tissues. Moreover, the optimization of the carrier had pursued nontoxicity, biocompatibility, and biodegradability [227].

NPs have various advantages such as small particle size, large surface area, and a potentially modifiable surface that prevents the enzymatic degradation of the drugs in the GIT; therefore, these are used as oral delivery systems for polynucleotides, proteins, and macromolecules. Moreover, they increase the GIT stability of acid-labile drugs. The character of NP is determined by the polymer from which it is composed. CS has an absorption-promoting effect due to its mucoadhesion properties and transient opening of the tight junctions of the mucosal cell membrane. In addition, the interaction between CS (positively charged) and mucin (negatively charged) leads to an increase in contact time between the drug and the absorptive surface, therefore CS increases the half time of clearance. The ability of CS to increase the membrane permeability depends upon its degree of deacetylation and molecular weight. As the degree of deacetylation increases, the charge density of CS also increases which in turn improves the drug transportation, which means that there will be increased epithelial permeability [52]. These CS properties are reflecting and advantageously utilized in CS oral drug delivery systems, as they are discussed for various categories of drugs in Sections Antidiabetic Drugs–Other Drugs.

##### Antidiabetic Drugs

Oral administration is a highly attractive approach for the delivery of protein drugs. However, there are three major problems in the absorption of protein and peptides using oral delivery. First, exposure to low pH in the gastrointestinal tract (pH 1–3 in the stomach) will lead to the deactivation of peptides and proteins due to oxidation, hydrolysis, or deamidation. Second, the intestinal epithelium is a major barrier because peptides and proteins, because of their high molecular weight and hydrophilicity, are difficult to diffuse across the lipid bilayer of cell membranes. And finally, various digestive enzymes in the GIT lead to the degradation of peptides and proteins. Therefore, it is very challenging to improve the bioavailability of peptide and protein drugs to achieve the level of diagnosis and treatment following oral administration [79,162]. To improve the quality of life of diabetic patients, oral delivery of insulin would be better than the subcutaneous injection, and the encapsulation of insulin for its oral delivery is a promising alternative [160].

A successful oral protein nanocarrier needs to exhibit the following characteristics: stability in the gastrointestinal (GI) tract, drug release at a specific site, overcoming high mucus turnover, mucoadhesive properties, and enhanced epithelial absorption [161,163].

Sudhakar et al. [160] reported the preparation of an oral insulin delivery system using thiolated chitosan NPs (TCNPs) loaded with insulin (Ins) and tested under in vitro and in vivo conditions. Increased and decreased levels of insulin and glucose, respectively, were observed in the blood when the Ins-TCNPs were orally administered in the diabetes-induced rats. Thus, the results suggested that the insulin stays for a significantly prolonged period to enhance biodistribution and bioavailability due to its interaction with the mucus of the intestine. This offers an improved oral insulin delivery system for diabetic patients.

The formation of NPs of insulin with CS and snail mucin represents a potentially safe and promising approach to protect insulin and enhance its peroral delivery. Mumuni et al. [160] prepared insulin-loaded NPs via the self-gelation method using CS and aqueous soluble snail mucin as natural polymers. The in vivo studies revealed a pronounced hypoglycaemic effect in diabetic rats after peroral administration of the insulin-loaded NPs compared to the effect caused by free oral insulin solution. However, the observed reduction of the blood glucose levels was lower than the effects observed in rats treated with subcutaneously administered insulin solution.

Tsai et al. [162] combined trimethyl chitosan, TMC (possessing excellent mucoadhesive and absorption-enhancing properties), with fucoidan, FD (possessing hypoglycemic effects and capacity to prevent diabetes-related complications), to develop a multifunctional nanoplatform for enhancing the transepithelial permeation of insulin through the intestinal epithelial cell barrier and inhibiting the α-glucosidase activity. The NPs were able to control the release rate of insulin at different pH, modulate the barrier function of the Caco-2 intestinal epithelial cell monolayers, and enhance paracellular transport of insulin across the monolayer.

Wong et al. [163] examined the potential of a complex coacervation technique for manufacturing insulin-loaded chitosan-Dz13Scr NPs. The complex coacervation technique used in the study could tighten the size uniformity and generate NPs with high encapsulation efficiency (88%). In vitro drug release model also confirmed that the NPs could withstand the acidic environment with only 13% of insulin released. The formulation could maintain its stability upon storage, promote the permeation of encapsulated insulin via paracellular absorption and endocytic pathway, and induced glucose uptake in the skeletal muscle cells. Therefore, this insulin-loaded chitosan-Dz13Scr NP was presented as a potential drug delivery system for the management of diabetes mellitus.

Abdel-Moneim et al. [164] were the first to develop and investigate a novel oral formula of polydatin-loaded chitosan nanoparticles (PD-CS NPs) to improve the therapeutic potential of PD against type 2 diabetes. The in vivo study revealed that PD-CSNPs exhibited significant antidiabetic efficacy in diabetic rats compared to free PD. To conclude, the current investigation proved the CS NPs could be promising nanocarriers for nontoxic and effective PD delivery against type 2 diabetes.

##### Anticancer Drugs

**Figure 26:**
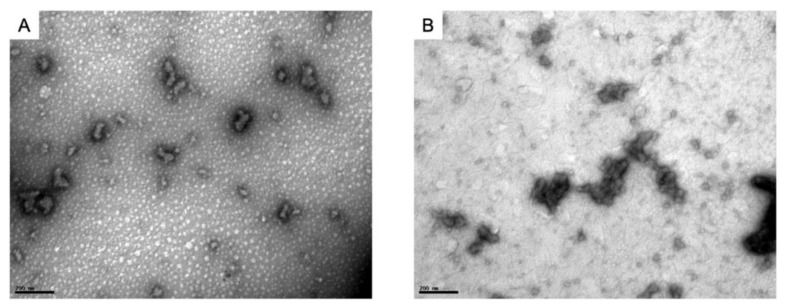
TEM images were obtained for the design nanoparticles. Both freshly prepared nanoparticles are in pH 3.0 (**A**)—5F/1C (5:1 ratio of the F and CS NPs) and (**B**)—MTX-loaded 5F/1C). Scale bar: 200 nm [165]. Reproduced with permission from Elsevier (Copyright©2020).

Methotrexate (MTX), known as an analog of folic acid, has been widely used as a chemotherapeutic agent. MTX’s mechanism of action is known to interfere with the formation of DNA, RNA, and proteins by blocking the action of dihydrofolate reductase leading to the inhibition of the metabolism of folic acid. MTX’s low oral bioavailability can be improved with its incorporation within delivery systems. Coutinho et al. [165] prepared and characterized fucoidan/ chitosan NPs loaded with MTX intended for lung cancer therapy. The TEM (transmission electron microscopy) images obtained for F-CS NPs and MTX-loaded F-CS NPs are presented in Figure 26. MTX-loaded NPs were 7-fold more effective in inhibiting lung cancer cells proliferation than the free drug, showing the potential of fucoidan-CS F-CS NPs to improve the cytotoxicity of free methotrexate on A549 lung cancer cells. These results also demonstrated that fucoidan/CS NPs may provide a suitable platform for poor-water soluble compounds’ oral delivery.

Curcumin (CUR) like other plant-based anticancer drugs is better tolerated than its chemotherapeutic cousins. However, oral administration of CUR results in very low systemic bioavailability. The aim of Sabra et al. [115] aimed to deliver CUR to the colon where it may exhibit its therapeutic effects locally. They developed a colon-targeted CUR-containing modified pectinate–chitosan nanoparticulate carrier system (M CUR-CS/P NPs) in simulated colonic conditions. Qualitative and quantitative analyses confirmed enhanced cellular uptake of CUR from M CUR-CS/P NPs compared to unmodified pectinate-CS NPs (U CUR-CS/P NPs). These findings point to the potential application of M CUR-CS/P NPs in the oral delivery of curcumin in the treatment of colon cancer.

To improve the oral administration of paclitaxel (PTX), Xavier et al. [166] synthesized a new poly(isobutyl cyanoacrylate) based nanocapsules by interfacial polymerization having a surface coated with CS. PTX was incorporated in the nanocapsules which cavity was filled out with copaiba oil and these nanocapsules were investigated for oral drug delivery. They showed interesting mucoadhesive properties with mucins and good association (9%) with the intestinal mucosa of the rat. Du et al. [167] prepared a novel chitosan-based multifunctional NPs using cationic polylysine (PL) polymer, L-cysteine (Cys), and poly(lactide) (PLA). PTX-Cys/PLA/CS NPs improved the distribution of PTX in tumor sites and presented better antitumor efficacy in Heps tumor-bearing mice and with less toxicity than other formulations. In conclusion, the CS-based NPs might be developed as a promising delivery vehicle for improving the oral bioavailability and therapeutic effect of hydrophobic antitumor drugs.

Tran et al. [168] developed interpolymer-complex structures composed of hydroxypropyl methylcellulose (HPMC), and chitosan knitted with D-α-tocopherol polyethylene glycol succinate (TPGS) to establish an oral nanoparticle delivery system for docetaxel (DTX) that could keep the drug dose from releasing into the gastrointestinal tract for at least 6 h. This study also illustrated the enhanced uptake of the NPs by the Caco-2 cells, implying enhanced absorption through the intestine. Therefore, these oral NPs can be considered for delivery systems of agents that are sensitive to the gastrointestinal tract so that they can be transported across the epithelial cells to the bloodstream to deliver the loading cargo at an optimal concentration.

##### Antihypertensive Drugs

A major challenge associated with the oral delivery of anti-hypertensive drugs is their poor water solubility and low oral bioavailability. Carvedilol (CAR), a potential beta-blocker is a hydrophobic drug exhibiting limited therapeutic effect through oral conventional drug delivery systems. Sharma et al. [169] developed NPs of CAR by using CS as a biodegradable polymer by ionic gelation technique using sodium tripolyphosphate as a crosslinking agent. The pharmacokinetic results revealed that the optimized CS NPs formulation has higher bioavailability than the marketed tablet formulation which indicates CAR-CS NPs as an effective strategy for the oral delivery of poorly water-soluble drugs.

##### Antioxidants

Pauluk et al. [170] developed zein NPs coated with CS as resveratrol (RVT) carriers. CS coating improved the protection of NPs against premature RVT release in simulated gastrointestinal fluids. CS also played an essential role in the adsorption of mucin on the surface of NPs, demonstrating its mucoadhesive properties. The results show the potential of CS-coated zein NPs as RVT carriers for oral administration.

##### Anti-inflammatory Drugs

Dexketoprofen trometamol (DT) belongs to the nonsteroidal anti-inflammatory drug (NSAID) group which is a rapidly acting analgesic ingredient. Because DT has a short half-life, high and frequent dosing is used in treatment. Ozturk et al. [93] produced DT-CS NPs by spray drying method for oral drug delivery. The results showed enhanced anti-inflammatory activity of DT-CS NPs with inhibition value comparable to the DT standard but at the one-fifth lower dosage. The DT-loaded CS-NPs seem to be a promising prolonged-release drug delivery system for oral administration with a low dose and high efficiency.

NPs may be also potential carriers for oral delivery of polysaccharides, for example, those from *Ophiopogon japonicus* (OJPs). OJPs are used to treat intestinal barrier defects, such as inflammatory bowel disease, but they are poorly absorbed after oral administration. This results in limited efficacy because of the low bioavailability (due to inflammatory bowel disease). Lin et al. [171] prepared OJPs/chitosan (CS)/whey protein (WP) co-assembled NPs. The results suggested that OJPs-CS/WP-NPs effectively protected the intestinal epithelial barrier integrity against the damage caused by lipopolysaccharide (LPS)-stimulated macrophage inflammation and attenuated the defects of intestinal epithelial TJ barrier and permeability. 

Ling et al. [172] tried to deliver therapeutic protein NPs encapsulated within gastro-protective microparticles (MPs) made from alginate and CS that subsequently release NPs of Salmonella effector enzyme in the small intestine and colon. Oral administration of Samonella effector enzyme (AvrA) NPs encapsulated in alginate/CS MPs delivered the protein to intestinal epithelia and reduced clinical and histological scores of inflammation in a murine DSS (dextran sodium sulfate)-induced colitis model.

##### Oral Vaccines

Mucosal vaccination is the most effective route to induce a local protective immune response against infections originating at mucosal surfaces (for vaccine delivery see also Section 3.4.10). The oral route offers potential for convenient administration of vaccines that achieve mucosal immune response following specific targeting of antigens to the gut-associated lymphoid tissue (GALT). The gastrointestinal tract, however, is an environment that presents several barriers to vaccine-mediated production of the mucosal immune response. The presence of these barriers necessitates the use of appropriate delivery systems to ensure antigen protection from degradation, enhanced uptake/absorption, and immune cell activation. CS has been explored as a component of particulate delivery systems with the potential to enable clinically effective oral administration of vaccines. This is because antigen encapsulation in particles is considered to be a key strategy in overcoming the hurdles of poor antigen immunogenicity and degradation in the gastrointestinal tract [174].

**Figure 27:**
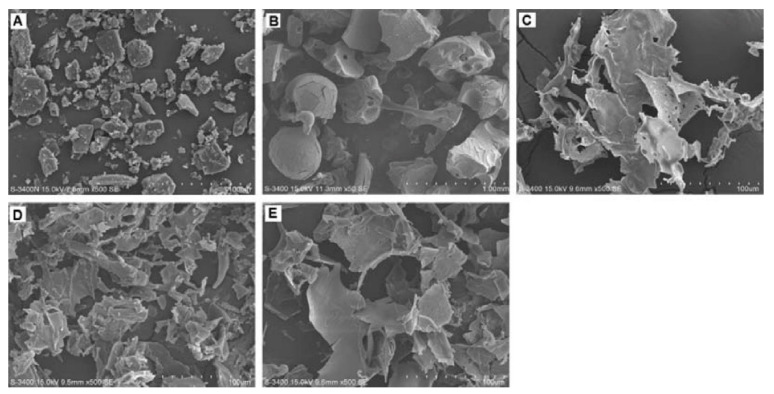
The SEM photographs of β-CD (**A**), CM-HP-β-CD (**B**), OVA (**C**), OVA-β-CD inclusion complex (**D**), and OVA-CM-HP-β-CD inclusion complex (**E**) [173]. Reproduced with permission from Elsevier (Copyright©2018).

Muye et al. [173] prepared a novel oral protein (OVA as a model antigen) delivery system for oral vaccination with enhanced intestinal penetration and improved antigen stability based on CS NPs and antigen-β-cyclodextrin and antigen-carboxymethyl hydroxypropyl-β-cyclodextrin (CM-HP-β-CD) inclusion complex (OVA-β-CD/CS and OVA/CM-HP-β-CD/CS). The SEM (scanning electron microscope) photographs of prepared NPs and inclusion complexes are illustrated in Figure 27. In vitro drug release studies, mimicking oral delivery conditions of the OVA-β-CD loaded CS NPs, showed a low initial release at pH 1.2 for 2 h less than 3.0% and a delayed-release which was below to 30% at pH 6.8 for subsequent 72 h. More importantly, after oral administration of the OVA-β-CD loaded CS NPs to Balb/c mice, OVA-specific sIgA levels in jejunum were 3.6-fold and 1.9-fold higher than that of an OVA solution and OVA loaded CS NPs, respectively. In vivo evaluation results showed that the OVA-β-CD loaded CS NPs could enhance their efficacy for inducing an intestinal mucosal immune response. In conclusion, these data suggested that CD/CS NPs could serve as a promising antigen-delivery system for oral vaccination.

The capacity of CS to facilitate drug delivery across mucosal surfaces is attributed to its mucoadhesive properties and its ability to open the epithelial tight junctions, as structures that keep adjacent epithelial cells near one another. CS can complex a model antigen into nano-size entities. Although these systems show interesting effects in vitro, namely notable augmentation of antigen delivery across an intestinal epithelial monolayer model, this does not reliably predict in vivo systemic immune response following oral delivery. Cole et al. [174] compared in vitro and in vivo antigen delivery effects of ultrapure CS chloride. They formulated CS NPs to incorporate OVA as a model antigen and characterized for size, charge, OVA complexation, and release. Nanocomplexes displayed favorable delivery properties, namely OVA release and no notable cytotoxicity. The OVA-CS complex markedly enhanced antigen delivery across Caco-2 monolayers. However, the system did not elicit notable in vivo immune responses (some mucosal response was apparent) following oral delivery. The study highlights that a clear effect on antigen permeability across epithelial monolayers in vitro may predict the in vivo mucosal but not systemic immune response following oral delivery.

Mucosal administration of the vaccine can produce a strong immune response. Antigens adhere to “M-cells”, present at the intestinal mucosa and the M-cells produce immunity after actively transporting luminal antigens to the underlying immune cells. Saraf et al. [175] prepared and characterized alginate-coated chitosan (ALG/CS NPs) loaded with HBsAg (hepatitis B antigen) as an antigen to produce immunity; they were additionally anchored with lipopolysaccharide (LPS) as an adjuvant. Results showed that the alginate coating effectively protected antigen at GIT in an acidic medium. The anchoring with LPS showed increased immunity as compared to other formulations (e.g., without LPS and alginate). Additionally, the NPs elicited significant sIgA at mucosal secretions and IgG antibodies in the systemic circulation. Thus, the prepared LPS anchored ALG-coated CS NPs may be a promising approach as a vaccine delivery system for oral mucosal immunization.

Another approach to improve oral vaccine efficiency is the use of layered double hydroxide (LDHs) NPs. They have shown excellent capability and good adjuvant function as a nanocarrier for protein antigen delivery to enhance the immune response. Furthermore, LDHs have good biocompatibility and low cytotoxicity. However, their oral vaccine delivery efficiency is limited due to acidic/enzyme degradation in the stomach and low bioavailability in the small intestine. To overcome these challenges, Yu et al. [176] developed an alginate–chitosan-coated LDH NP (ALG/CS/LDH NP) based nanocomposites as a carrier system for oral protein vaccine delivery (BSA and OVA as model antigens). The protein release profile of the ALG/CS/LDH NP nanocomposites indicated that the ALG/CS coating could partially protect protein release at the acidic condition (pH 1.2). The cellular uptake results have demonstrated the ALG/CS coating LDHs can significantly enhance the attachment and internalization of proteins in the intestine cells and macrophages. The enhancement of the ALG/CS coating LDHs is attributed to their strong interaction with the saccharide receptors and surface proteins. In conclusion, ALG/CS-LDHs have shown great potential for oral protein vaccine delivery.

##### Oher Drugs

Liu et al. [79] developed promising chitosan-modified dual drug-loaded NPs containing salmon calcitonin (sCT) and puerarin (PR), CS-sCT/PR-NPs. In vivo, the oral absolute bioavailability of sCT in CS-sCT/PR-NPs was 12.52 ± 1.83%, approximately 1.74-fold higher than that of the NPs not co-loaded with PR. In conclusion, the CS-based NPs and introduction of PR as a protease inhibitor improved the oral bioavailability of sCT and had the potential to be developed as an oral delivery system of peptide drugs.

Low molecular weight heparin, anticoagulant for the treatment of vascular disorders can only be administered by parenteral routes for clinical applications. Dong et al. [177] prepared chitosan-based polymer-lipid hybrid nanoparticles (PLNs) by a self-assembly method and evaluated their use as the carrier for oral absorption enhancement of enoxaparin. PLNs with the optimal composition significantly enhanced the oral bioavailability of enoxaparin with a 4.5-fold increase in AUC (area under the curve) in comparison with a solution of enoxaparin. In conclusion, glycerol monooleate (GMO)/CS-based PLNs can provide a new insight to develop an orally applicable delivery system for hydrophilic macromolecules. Their absorption can be enhanced with the proposed PLNs and the preparation of these PLNs was also found to be easy comparing to other similar methods.

CS NPs hold promising features also as an oral delivery vehicle for various lipophilic drugs. Astaxanthin (ASTX) has been reported as a potential therapeutic agent for hepatic fibrosis treatment. However, its therapeutic effect is limited due to low bioavailability and poor aqueous solubility. Hu et al. [114] fabricated biopolymer-based NPs using stearic acid-chitosan conjugate (SA-CS) and sodium caseinate (NaCas) via ionic gelation. Its nanostructure was cross-linked using oxidized dextran (Odex) via Schiff base reaction. Compared to the anti-fibrogenic effect of free ASTX in LX-2 cells, the encapsulated ASTX demonstrated dramatically enhanced cellular bioactivity, as evidenced by significantly lower TGFβ1-induced fibrogenic gene (ACTA2 and COL1A1) expression level, as well as α-SMA and COL1A1 protein levels.

Raloxifene, marketed as raloxifene hydrochloride (RLX), is the only ‘selective estrogen receptor modulator’, approved worldwide for the prevention and treatment of postmenopausal osteoporosis and fragility fractures. The oral bioavailability of RLX is poor (<2%) with high inter-patient variability. Murthy et al. [178] in their study developed and evaluated soy lecithin-chitosan hybrid NPs to improve the oral bioavailability of RLX. Pharmacokinetic studies in female Wistar rats showed significant improvement (ca. 4.2 fold) in the oral bioavailability of the drug when loaded into the NPs. Further, the modified everted gut sac study showed that these NPs are taken up by active endocytic processes in the intestine. The ex vivo mucoadhesion studies proved that the NPs bound to the mucus layer of the intestine, which in turn correlated with reduced excretion of the drug in feces.

#### 3.4.2. Ocular Drug Delivery

Ophthalmic diseases are generally treated using topical instillation—eye drops of active compounds. However, the method of treatment has been hindered by the inherent defense function of the eye [11]. The corneal epithelial cells restrict drug proliferation due to blocking the passage of the drug through the cornea. Drugs are degraded by metabolic enzymes in the ocular tissues. Drugs applied to the surface of the eyes are excreted through nasolacrimal ducts. Additionally, spontaneous reject of foreign substances by the oculus also impedes drug transport. Thus, ocular administration remains a clinical challenge [33]. To increase ocular absorption and improve the drug bioavailability, the research of new drug carriers has become the development trend. CS possesses favorable characteristics such as nontoxicity, biodegradability, and biocompatibility, which make it a suitable choice for ocular drug delivery systems. In addition, it is naturally mucoadhesive, which increases the ocular surface duration of drugs. CS also possesses in situ gelling properties due to which, when it is applied on the ocular surface in liquid form, it gets transformed into gel later. This leads to the improvement of the ocular residence time and therapeutic efficacy of the drug [52].

**Figure 28:**
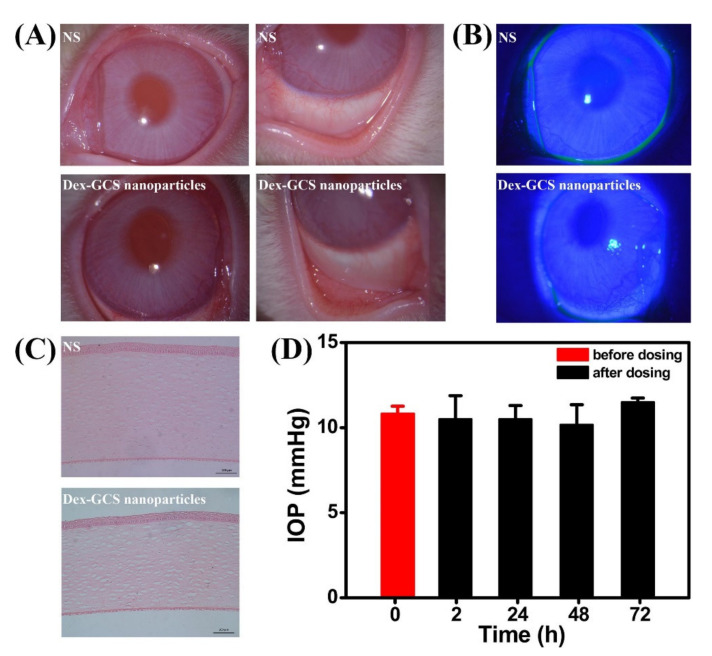
(**A**) Slit-lamp observations of the eyes treated with NS and DEX-GCS NPs at 2 h after administration; (**B**) fluorescein sodium staining of the cornea treated with NS and DEX-GCS NPs at 2 h after administration; (**C**) H&E section of the cornea treated with NS and DEX-GCS NPs at 24 h after administration; and (**D**) the changes in intraocular pressure (IOP) after treatment with Dex-GCS NPs [179]. Reproduced with permission from Elsevier (Copyright©2019).

Efficient ocular drug delivery to the posterior segment of the eye by topical administration is a great challenge to pharmacologists. Dexamethasone (DEX) is one of the most widely used steroidal agents for the treatment of various inflammatory disorders of the eye with high potency and effectiveness. Yu et al. [179] prepared a series of dexamethasone-glycol chitosan (DEX-GCS) conjugates. The eye irritation of DEX-GCS NPs was investigated in Japanese white rabbits using a modified Draize test. As shown in Figure 28A, the eyes treated by DEX-GCS NPs and NS at 2 h did not present any conjunctival congestion, corneal opacity, or iris-inflammatory exudation. Fluorescein staining indicated that there were no ulcers or defects on the corneal epithelial layer of the eyes treated with the DEX-GCS NPs and normal saline after 2 h (Figure 28B). Moreover, histological observation indicated that there were no obvious morphological or pathological changes to the cornea after treatment with DEX-GCS NPs and normal saline after 24 h (Figure 28C). Moreover, the intraocular pressure (IOP) did not change obviously for the eyes treated with DEX-GCS NPs during the 3-day study (Figure 28C). The proposed NPs showed good ocular tolerance and provided a relatively longer precorneal duration compared with that of the aqueous solution formulation, which suggested that the self-assembled DEX-GCS NP might be a promising candidate for ophthalmic drug delivery.

In another work, Wang et al. [148] developed a hybrid nanocomposite (layered double hydroxide (LDH)-glutathione-glycylsarcosine (GSH-GlySar)-carboxymethyl chitosan (CMC)) offering a simple and efficient strategy for topically administered dexamethasone disodium phosphate (DEXP) delivery to the posterior segment of the eye. The in vitro experiments on human conjunctival epithelial cells showed no cytotoxicity (LDH concentration ≤100.0 µg/mL) but enhanced permeability for hybrid nanocomposites. Results of the in vivo precorneal retention study showed an 8.35-fold, 2.87-fold, and 2.58-fold increase in AUC 0–6 h, C max and MRT for DEXP-CMC/GSH/GlySar/LDH (10:1) hybrid nanocomposite eye drops, respectively, compared to that of the commercial product. In the evaluation of tissue distribution of rabbit’s eyes, DEXP of the DEXP-CMC/GSH/GS/LDHs (10:1) nanocomposite retained in the target of the choroid-retina for 3 h with final concentration at 120.04 ng/g. Furthermore, the results of fluorescence imaging and tissue distribution suggested that the intraocular transport pathway for the hybrid nanocomposites is the conjunctival-scleral route. Consequently, the developed hybrid nanocomposites offer a simple and efficient strategy for topically administered drug delivery to the posterior segment of the eye.

Liposomes have shown significantly improved penetration potential and compatibility upon comparison with other systems. A coating of liposomes with CS can further improve their availability owing to the mucoadhesive properties which increase the affinity of composite membranes. Khalil et al. [180] prepared chitosan-coated liposomes (CS/LIP). Conventional liposomes encapsulating triamcinolone acetonide (TA) were compared with their CS-coated counterpart for the drug loading and release studies. CS/LIP showed a higher encapsulation efficiency (74%), and a highly positive surface charge (+41.1 Mv), increased retention time, and sustained release. The results showed successful penetration of the construct via the corneal mucosal barrier and its accumulation in the vitreous body. The analysis shows that this CS-based liposomal construct can be employed as a potential topical delivery system for treating posterior segment diseases.

Levofloxacin (LFX) is a synthetic third generation of fluoroquinolones with a broad spectrum of antibacterial activity. Ameeduzzafar et al. [181] tried to combine in-situ gel and NPs. They used CS NPs to encapsulate LFX for the treatment of ocular infection. The antimicrobial study revealed that the developed formulation possessed higher antibacterial activity against *P. aeruginosa* and *S. aureus*. The pharmacoscintigraphic study results revealed the reduced corneal clearance, nasolacrimal drainage as well as higher retention of LFX in comparison to an LFX solution. Thus, the LFX loaded CS NP in situ gel system was found to be efficient for ocular delivery of LFX.

For anti-inflammatory ocular treatment, Hanafy et al. [32] prepared a novel formulation of self-assembled NPs via complexation of CS and, the counter-ion, sodium deoxycholate (SD), loaded with the poorly-water-soluble prednisolone acetate (PA). Drug release of PA as received, in simulated tears fluid (pH 7.4), showed a twofold increase (reaching an average of 98.6% in 24 h) when incorporated into an optimized nanoparticle gel formulation (1:5 CS-SD). The anti-inflammatory effect of PA NPs loaded gel on female guinea pig eyes was significantly superior to that of the micronized drug-loaded gel (*p* < 0.05). The prepared ophthalmic NPs loaded gel for delivering PA had the potential at delivery of PA in treating inflammatory ocular diseases.

Baicalein (BAI) is one of the main ingredients of Scutellariae radix. Clinically, it is used to depress apoptosis of retinal ganglion cells which is related to visual decline. Additionally, BAI protects against retinal ischemia by anti-oxidation, anti-apoptosis, and anti-inflammatory. Therefore, BAI has great potential in preventing and treating various ocular diseases such as keratitis and glaucoma. However, molecular dynamics simulation data showed that BAI had a poor membrane permeability, which limited the ocular bioavailability. To improve its ocular bioavailability, Li et al. [33] prepared and characterized trimethyl chitosan-coated lipid nanoparticles of baicalein (TMC-BAI-LNPs). In vitro drug release revealed that TMC-BAI-LNPs exhibited a sustained-release effect. In vivo studies indicated that TMC-BAI-LNPs had no ocular irritation and the AUC of TMC-BAI-LNPs was 3.17-fold higher than that of the control (*p* < 0.01). The results indicated that TMC-BAI-LNPs might open up a new avenue for ocular administration. 

Glaucoma is the most common form of optic nerve degeneration that leads to progressive, irreversible blindness. This disease is characterized by increased intraocular pressure (IOP). The main object in the treatment of glaucoma is to reduce IOP to the normal range (16–18 mmHg). Dorzolamide (DRZ), a carbonic anhydrase inhibitor (CAI), is the most common drug used to treat glaucoma by reducing IOP via decreasing aqueous humor secretion (through the inhibition of carbonic anhydrase isoenzyme). Shahab et al. [82] developed dorzolamide-loaded chitosan-coated polycaprolactone nanoparticles (DRZ-CS-PCL-NPs) for improved ocular delivery. The corneal flux experiment showed many fold enhancement in permeation across goat cornea. DRZ-CS-PCL-NPs exhibited 3.7-fold higher mucoadhesive strength compared to the control. Furthermore, the histopathological assessment and HET-CAM study revealed that the DRZ-CS-PCL-NPs were non-irritant and safe for ocular administration. Therefore, it can be concluded the optimized DRZ-CS-PCL-NPs are safe and have the potential for successful ocular delivery and improved therapeutic efficacy.

Another drug latanoprost reduces IOP by increasing the uveoscleral outflow. Despite its potency, long-term daily application of it may cause undesirable side effects and may require more than one medication for IOP control. Recent studies have suggested that oxidative stress in the trabecular meshwork (TM) plays an important role in the pathogenesis of impaired trabecular outflow facilities. Cheng et al. [182] combined latanoprost with curcumin, a natural phenolic compound possessing anti-oxidant and anti-inflammation properties. They developed a thermosensitive hydrogel containing latanoprost and curcumin-loaded nanoparticles (CUR-NPs) and evaluated its possible therapeutic effects with cultured human TM cells under oxidative stress. The results demonstrated that 20 μM of CUR-NPs might be the optimal concentration to treat TM cells without causing cytotoxicity. 

Intravitreal injections of bevacizumab (BEV) have been used for the treatment of several eye disorders such as diabetic macular edema, central retinal vein occlusion, proliferative diabetic retinopathy, rubeosis iridis, pseudophakia cystoid macular edema, choroid neovascularization, secondary to pathologic myopia. The in vivo studies revealed the absence of ocular toxicity for BEV administered by intravenous injection. Due to its short half-life and the necessity of frequent intravitreal injection, a method for sustained delivery is required. Savin et al. [48] obtained, for the first time, polymeric nanocarriers based on the chitosan grafted-PEG-methacrylate derivative. They tried to prepare potentially non-toxic micro/nanoparticles (MNPs). The double crosslinking (ionic and covalent) procedure in the reverse emulsion provided the mechanical stability of the polymeric nanocarrier. The potential of MNPs as a drug delivery system was analyzed by loading a full-length monoclonal antibody BEV. The proposed MNPs were found to be suitable from the cytotoxicity and hemocompatibility point of view enabling their potential use as a delivery system for the treatment of a posterior segment of the eye conditions. 

#### 3.4.3. Nasal Drug Delivery

Nasal delivery is a non-invasive technique that allows the administration of drugs both locally and systemically avoiding the typical gastrointestinal degradation of oral administration and the effect of hepatic metabolism [52]. It is an alternative method of topical administration for anti-inflammatory, antibacterial, nasal congestion, or hemostasis therapy, but nasal administration can even pass the blood–brain barrier (BBB) that has been proven to conveniently direct drug delivery from the nose to the brain, NTB [11]. Nasally administered drugs can rapidly reach the brain through three different pathways (i.e., three ways of nasal absorption): the first is the olfactory nerves, which represent the main direct pathway for the NTB drug delivery; then the trigeminal nerves, which have nerve endings in the respiratory epithelia; finally, the respiratory epithelium through which the drugs reach the circulation and subsequent cross the BBB. However, the NTB delivery is characterized by several limitations such as the small volume of the nasal cavity, the mucociliary clearance, the enzymatic degradation, the low drug retention time, the potential nasomucosal toxicity, the technique of drug administration and deposition, and the necessity of a suitable delivery device. The rich vascularization of the nasal mucosa provides a series of unique attributes that can increase safety, patient compliance, the rate of absorption of the drug, and, consequently, the speed of onset of the therapeutic effect. Furthermore, the nasal mucosa is easily accessible compared to other membranes and it is a favorable entry route for both small and large molecules. The nasal bioavailability of small molecules is good and the drugs that are not absorbed orally can be conveyed to the nasal systemic circulation [186]. 

CS increases the permeability of the hydrophilic drugs, nucleic acids, proteins, and peptides across the nasal epithelium as they pose difficulty due to their low permeability. Certain characteristics such as weight, lipophilicity, and charge of the drug affect its nasal absorption. The drugs which are unable to cross the nasal membrane undergo mucociliary clearance. A mucoadhesive system can help to overcome this problem and CS possesses mucoadhesion properties along with low toxicity, biodegradability, and biocompatibility, owing to its application in nasal delivery [52].

##### Topical Nasal Delivery

According to the therapeutic effect, nasal delivery can be topical or systemic. The first one includes mostly treatment of allergic rhinitis. Allergic rhinitis is a chronic airway inflammatory disease. CS is widely used for the preparation of different nasal mucoadhesive formulations that can be retained for an extended time in the nasal cavity and, at the same time, it can enable the transport of drugs across the membrane. Cromolyn (CRO) inhibits the degranulation of sensitized mast cells, thereby blocking the release of inflammatory mediators. Abruzzo et al. [183] prepared new mucoadhesive nasal decongestant NPs obtained by direct crosslinking between CS and CRO. The obtained positively charged NPs, sized 180–400 nm, showed interesting properties in terms of yield, mucoadhesion, encapsulation efficiency, and drug loading. Release and permeation/penetration data indicated the ability of the NPs to retain a high amount of CRO inside the mucosa, which is rich in mast cells. These findings suggest developing decongestant NPs for the potential treatment of allergic rhinitis.

Cetirizine (CTZ) is a common H1-antihistamine in allergic disorders therapy but hydrophobicity, the rapid disintegration of conventional dosage forms, and irritation to the nasal mucosa limit its application. Sun et al. [184] synthesized DCHBC NPs (deoxycholate chitosan-hydroxybutyl CS NPs) with CTZ covalently grafted and free CTZ encapsulated as nasal adaptive nano-drug delivery systems. Incubating with lysozyme (30 μg/mL), DCHBC NPs swelled and exhibited a ca. 2-fold increase (*p* < 0.01) of sizes, with additional CTZ releasing (ca. 5%) attributing to the digestion of polysaccharide backbone covalently connected with CTZ. Stimuli-responsive DCHBC NPs might hold tremendous potential as nasal adaptive delivery vehicles in allergic airway inflammatory diseases therapy.

##### Systemic Nasal Delivery

The systemic nasal drug delivery is represented by nasal vaccines (for vaccine delivery see also Section 3.4.10). Intranasally administered vaccines remain in the nasal cavity for about 15 min only; they have to be transported over a very small distance and have to be protected from low pH and enzymatic degradation. Mucoadhesive NPs are recognized for the benefits they offer via nasal delivery, such as extended retention time of the vaccine on the mucosa. Various CS-based vaccines for nasal delivery were prepared, containing for example influenza, diphtheria, and pertussis antigens [52]. Dumkliang et al. [185] explored intranasal administration as a non-invasive route to overcome the limitations of conventional subcutaneous injection for Japanese encephalitis (JE) immunization. The purpose of this study was to evaluate the immunization effect of live attenuated Japanese encephalitis-chimeric virus vaccine (JECV)-loaded mucoadhesive NPs via the IN route to improve the mucosal immunization against JE. The used NPs were based on chitosan or chitosan maleimide (CM), a novel mucoadhesive polymer. The results revealed that such IN immunization stimulated seroprotection following PRNT50 (plaque reduction neutralization titer) evaluation. Comparing to the SC immunization in mice, this IN immunization provided a higher sIgA level, leading to an improved mucosal immune response. In addition, the CS-based NPs showed an adjuvant effect on the IN vaccine due to their mucoadhesive and antigen-uptake properties. The JECV-loaded mucoadhesive NPs represent a promising approach for IN vaccination as an alternative route for JE protection due to the stimulatory effects on both mucosal and systemic immune responses.

##### Nose to Brain Delivery

Hydrophilic molecules and high molecular weight compounds are not favorably absorbed by the brain due to the presence of BBB and blood-cerebrospinal fluid barrier (BCB) that cause a selective permeability to circulating molecules. In the last years, NTB (nose to the brain) delivery is an emerging strategy to overcome these barriers and to deliver the drug directly into the brain. Pharmaceutical nanotechnologies appear as an ideal formulation strategy for NTB delivery. Piazzini et al. [186] developed human serum albumin nanoparticles (HSA NPs) coated with chitosan as a nose-to-brain carrier to deliver the anti-Alzheimer drugs tacrine and R-flurbiprofen. The CS-coated NPs showed better mucoadhesion on ex vivo rabbit nasal mucosa and the higher penetrating potential of HSA/CS NPs concerning uncoated NPs. They have also the advantage of opening the tight junctions between hCMEC/D3 cells, by decreasing the levels of ZO-1 expression, allowing for the transport of molecules across the barrier.

Neurologic disorders such as Parkinson’s disease (PD) require direct transport of therapeutic substances to the brain to maximize drug concentration in their active site. PD is characterized by neurodegeneration and loss of dopaminergic neurons in the Central Nervous System (CNS). To date, the standard treatment for controlling PD motor symptoms is based on the dopamine (DOPA) replacement strategy which aims to compensate for the loss of dopaminergic neurons and re-establish satisfactory levels of the neurotransmitter. DOPA is unable to overcome the BBB because of its high hydrogen-bonding potential, complete ionization at physiological pH, and extensive metabolism by the oral route of administration. Therefore, most interest has been focused on the development of DOPA-loaded nanocarriers as an innovative tool for the PD treatment which should be able to cross the BBB and enable a sustained delivery of the neurotransmitters to the brain. Ropinirole hydrochloride (RH) is a non-ergoline agonist that acts on D2- and D3-receptors in the brain. Chatzitaki et al. [187] developed a PLGA and PLGA/CS NPs with mucoadhesive properties able to encapsulate RH and suitable to promote RH delivery across the nasal mucosa. RH-loaded PLGA/CS NPs showed a complete release of the drug in a simulated nasal electrolyte solution (SNES) throughout 24 h. In addition, they increased the permeation of RH through sheep nasal mucosa by 3.22-fold in comparison to PLGA NPs. None of the RH-loaded NPs induced the hemolysis in whole blood or the production of reactive oxygen species (ROS) in Raw 264.7 cells. In their turn, PLGA/CS NPs decreased cell viability of Raw 264.7 cells and peripheral blood mononuclear cells (PBMCs) in a concentration-dependent manner. These results revealed that PLGA/CS NPs could be a valuable carrier for the delivery of RH to the CNS, opening a new path for Parkinson’s disease therapy.

Cassano et al. [188] synthesized a novel carboxylated chitosan-dopamine (DOPA) and -tyrosine (Tyr) conjugates as systems for improving the brain delivery of the DOPA following nasal administration. For this purpose, ester or amide conjugates were synthesized by N,N-dicyclohexylcarbodiimide (DCC) mediated coupling reactions between the appropriate N-tertbutyloxycarbonyl (Boc) protected starting polymers N,O-carboxymethyl chitosan and 6-carboxy chitosan, and DOPA or O-tert-Butyl-L-tyrosine-tert-butyl ester hydrochloride. The results demonstrated that N,O-carboxymethyl CS-DOPA (N,O-CMC-DOPA) conjugate was the most mucoadhesive polymer in the series examined, and together with the 6-carboxy CS-DOPA conjugate, they were able to release the neurotransmitter in simulated nasal fluid (SNF). The MTT assay showed that the starting polymers, as well as all the prepared conjugates in olfactory ensheathing cells (OECs), resulted not toxic at any concentration tested. Likewise, the three synthesized conjugates were not cytotoxic as well. Cytofluorimetric analysis revealed that the N,O-carboxymethyl CS DOPA conjugate was internalized by OECs in a superior manner at 24 h compared with the starting polymer. Overall, the N,O-CMC-DOPA conjugate seems promising for improving the delivery of DOPA by nose-to-brain administration. 

Rotigotine, a non-ergoline dopamine agonist, is highly effective for the treatment of PD. However, despite its therapeutic potential, its’ clinical applications were hindered due to low aqueous solubility, first-pass metabolism, and low bioavailability. Bhattamisra et al. [191] developed rotigotine-loaded CS NPs (RNPs) for nose-to-brain delivery and evaluated its neuronal uptake, antioxidant and neuroprotective effects using cell-based studies. Behavioral and biochemical testing of RNPs in haloperidol-induced PD rats showed a reversal of catalepsy, akinesia, and restoration of swimming ability. A decrease in lactate dehydrogenase (LDH) and an increase in catalase activities were also observed in the brain tissues. The results from the animal model of PD showed that intranasally administered RNPs enhanced brain targeting efficiency and drug bioavailability. Thus, RNPs for nose-to-brain delivery have significant potential to be developed as a treatment approach for PD.

Huntington’s disease (HD) is an inherited autosomal-dominant neurodegenerative disease characterized by a progressive deterioration in cognition, mood, and motor control. The mutated HTT gene (mHTT) produces a misfolded protein that accumulates in neural cells causing gradual neuronal dysfunction and degeneration. Therapies to lower gene expression in brain disease currently require chronic administration into the cerebrospinal fluid (CSF) by intrathecal infusions or direct intracerebral injections. Though well-tolerated in the short term, this approach is not tenable for a lifetime of administration. Sava et al. [189] synthesized enriched CS-based NPs loaded with anti-HTT small interfering RNA (siRNA) for nose-to-brain delivery. The NPs were studied in a transgenic YAC128 mouse model of HD. A series of CS-based NP formulations encapsulating anti-HTT siRNA was designed to protect the payload from degradation “en route” to the target. Factors to improve the production of effective nanocarriers of anti-HTT siRNA were identified and tested in a YAC128 mouse model of Huntington’s disease. Some of the formulations were identified to be effective in lowering HTT mRNA expression by at least 50%. Intranasal administration of NPs carrying siRNA is a promising therapeutic alternative for safe and effective lowering of mutant HTT expression.

Migraine is a neurovascular disorder that involved dilatation of cerebral arteries and is commonly associated with a symptom such as nausea, sensitivity to light or sound, vomiting, and urinary frequency. Zolmitriptan (ZOL) is one of the most common types of triptans, serotonin 5-hydroxytryptamine receptor agonists, which inhibit the peripheral trigeminovascular system and can access central sites in the brain stem. ZOL occurs at low blood levels after therapeutic administration, it has low bioavailability (approximately 40%) when administered orally due to hepatic first-pass metabolism. Kherzi et al. [190] prepared a nasal spray from ZOL-loaded CS NPs and evaluated the drug for its pharmacokinetic properties. Amounts of the drug in the plasma from the test formulation, standard marketed drug (Zolmist), and standard drug solution at 10 min were found to be 41.37 ± 2.31, 34.76 ± 4.22, and 23.74 ± 2.42 ng/mL, respectively. These data indicated significantly (*p* < 0.05) a higher amount of the drug being delivered from the test formulation compared to both standard groups. The amount of ZOL present in brain tissue (Olfactory lobe) at 60 min was found to be 15 ± 0.08, 13 ± 0.14, and 8 ± 0.13 ng/g for the test formulation, marketed standard, and standard drug solution, respectively. This indicated a significantly (*p* < 0.05) higher amount of drug absorption in the brain tissue from the test formulation compared to both standard groups. The pharmacokinetic studies of a nasal spray containing ZOL-loaded CS NPs proved rapid onset of action in animals and this formulation is promising in the treatment of migraine.

#### 3.4.4. Pulmonary Drug Delivery

Drug delivery to the lungs has various advantages such as rapid and sustained drug delivery, high efficacy, no first-pass metabolism, and both local and systemic effects can be achieved. The factors which contribute to the enhanced drug delivery via lungs are the large surface area of lungs, thin absorption barrier, and high vascularity [52].

NPs can cross the cellular barrier independent of the energy supply. They can be designed to be taken up by macrophages for delivery of drugs directly to bacteria and thus treat diseases such as tuberculosis [194]. NPs can be used to deliver macromolecular drugs such as peptides and protein through the lung for the treatment of systemic or local diseases. They are effective for lung cancer treatment in association with improved drug accumulation inside the tumors due to facilitating their passive as well as active delivery [193,228]. NPs are also used to treat mucus hypersecretion and severe inflammatory lung diseases namely asthma [196], cystic fibrosis, and chronic obstructive pulmonary disease [195] due to their ability to provide sustained drug release, overcome airway hypersecretion, and target diseased cells or tissues through matrix decoration with a homing device.

However, NPs are exhaled from the lungs after pulmonary administration. The inhaled particles should have an aerodynamic diameter between 1 and 5 μm to enable the drugs to be deposited in the deep lungs. CS NPs are exhalation prone and agglomerative to pulmonary inhalation. Alhajj et al. [228] aimed to develop a new NPs lung delivery approach possible for use in lung cancer treatment. They physically blended CS NPs with fine lactose-PEG3000 microparticles, Lac/PEG3000 MPs (~5 μm) to reduce their agglomeration through surface adsorption phenomenon. This carrier exhibited a comparable inhalation performance with the commercial dry powder inhaler products (fine particle fraction between 20% and 30%). Cascade impactor analysis indicated that the aerosolization and inhalation performance of the CS NPs was promoted by their higher zeta potential, circularity, and larger size attributes. These properties led to reduced inter-nanoparticulate aggregation and favored the NPs interacting with the Lac/PEG3000 MPs that aided their delivery into deep and peripheral lungs.

**Figure 29:**
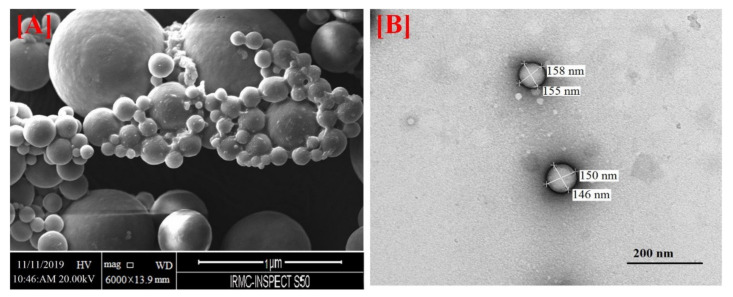
Scanning electron microscopy (SEM) (**A**) and transmission electron microscopy (TEM) (**B**) images of CS-coated-CAT-loaded PLGA NPs [193]. Reproduced with permission from Elsevier (Copyright©2020).

Ahmad et al. [193] studied possibilities to improve the lungs bioavailability of an anti-lungs cancer drug catechin hydrate (CAT) via the direct nose-to-lungs delivery. For this purpose, they prepared novel chitosan-coated PLGA NPs. SEM (A) and TEM (B) images of CS-coated-CAT-loaded PLGA NPs are illustrated in Figure 29. Higher entrapment efficiency was observed for the CAT-CS/PLGA NPs. The release pattern of the CAT-CS/PLGA NPs was found to favor the release of entrapped CAT within the cancer microenvironment. The CAT-CS/PLGA NPs exposed on H1299 cancer cells up to 24.0 h were found to exhibit higher cytotoxicity as compared to a CAT-solution. The CAT-CS/PLGA NPs showed higher apoptosis of cancer cells after their exposure as compared to the CAT-solution. The CAT-CS/PLGA NPs showed tremendous mucoadhesive nature as compared to the CAT-solution. The improved Cmax (668.24 ± 29.66 ng/mL) with AUC0–24 (11370.02 ± 191.05 min × ng/mL) was observed extremely significant (*p* < 0.001) via i.n. as compared to **oral** (Cmax 208.76 ± 17.01 ng/mL, AUC0–24 2223.77 ± 42.08 min × ng/mL) and i.v. (Cmax 469.31 ± 32.96 ng/mL, AUC0–24 6208.00 ± 89.67 min × ng/mL) in the Wistar rat’s lungs. The CS/PLGA NPs system was successfully designed and safely delivered CAT to the lungs without causing any risk. The CAT-CS/PLGA NPs were showed to have a significant role in the enhancement of lungs bioavailability and, thus, represent a promising approach to treat lung cancers.

Tuberculosis is one of the main causes of death worldwide, being the leading cause of a single infectious agent. Rifampicin (RIF) is a drug used in tuberculosis treatment being the first choice in association with other drugs for long periods, resulting in low adherence to the oral conventional treatment. Furthermore, RIF shows a low aqueous solubility, and thus, low bioavailability. Solid lipid nanoparticles (SLNs) can improve the efficacy of antituberculosis drugs and minimize the adverse effects of entrapped drugs. Their association with CS improves the mucoadhesive properties of the NPs. Vieira et al. [194] developed and characterized SLNs loaded with RIF aiming to enhance mucoadhesion of the SLNs and, consequently, internalization by the alveolar macrophages (AMs). Prepared SLNs coated with CS by the association method (CS SLNs) showed an effective mucoadhesive profile, verified by the turbidimetry and surface loading method, corroborated with the cellular assays. The presence of CS in the CS SLNs promotes not only enhanced mucoadhesive properties of the SLNs but also their higher permeability in the human A549 cell line, suggesting that the safe RIF-CS SLNs can be used as a promising drug delivery system for improving tuberculosis treatment.

Asthma can be well managed with glucocorticoids and long-acting β-agonists. However, higher doses have proved to be both clinically ineffective and potentially detrimental, where poorly treated inflammation causes repeated exacerbations and sudden deaths in severe asthmatic conditions. An alternative anti-inflammatory intervention, capacitated to control airway remodeling and hypersensitivity without the risk of adverse effects, is crucial to achieving effective disease control and prevention of repeated exacerbations. Most of the pulmonary therapeutics suffer from limitations like poor bioavailability and short half-life. The development of new formulations tailored with suitable surface chemistry and actuation technique for effective drug deposition, interaction, and transportation across the mucus layer is crucial to achieving a high therapeutic index. Vibrating mesh nebulizers are recognized as the most efficient actuation technique over conventional inhalers for drug deposition. Dhayanandamoorthy et al. [196] explored hyaluronic acid (HA) decorated, ferulic acid (FA) loaded chitosan NPs (FA-HA/CS NPs) aerosolized using vibrating mesh nebulizer as a strategic combination of drug, nanocarrier, and delivery device for effective asthma control. In vivo inhalation toxicity assessment confirmed safety, while FA-HA/CS NPs prophylaxis mitigated the inflammation, airway hypersensitivity, and remodeling in OVA-induced mice models of asthma. Thus, the results accentuated the role of pro-pulmonary surface chemistry conferred by HA functionalization that improved (i) thermal stability (as indicated by thermogravimetric analysis) and (ii) therapeutic efficacy of FA, by facilitating better interaction and transportation across mucus barrier (which otherwise suffers from poor bioavailability and rapid metabolism).

Etophylline (ETO), a bronchodilator used to treat airway diseases like asthma, chronic bronchitis, and chronic obstructive pulmonary disease (COPD), works by relaxing the smooth muscles of the lungs and dilating the bronchioles. Currently, tablet and injection dosage forms are commercially available. However, poor bioavailability with oral dose (owing to ETO incomplete absorption and high first-pass effect) and patient’s discomfort with painful injectable administration calls for the alternative formulation approach. Pardeshi et al. [195] fabricated the ETO encapsulated mannose-anchored N,N,N-trimethyl chitosan nanoparticles (Mn-TMC NPs). The prominent characteristics like biocompatibility, controlled release, targeted delivery, high penetrability, enhanced physical stability, and scalability marked the Mn-TMC NPs as a viable alternative to various nanoplatform technologies for effective drug delivery. Mannosylation of TMC NPs led to the evolution of a new drug delivery vehicle with gratifying characteristics, and potential benefits at efficient drug therapy. It is widely accepted that following pulmonary administration, i.e., the introduction of mannose to the surface of drug nanocarriers, provides selective macrophage targeting via receptor-mediated endocytosis. The in vivo pharmacokinetic studies in a Wistar rat model revealed a significant improvement in the therapeutic efficacy of ETO, illustrating mannosylation of CS NPs as a promising approach for efficient therapy of airway diseases following pulmonary administration

Tobacco smoking and nicotine addiction are common public health problems all over the world causing to death of more than 6 million people per year. The harmful effects of tobacco smoking are primarily due to the other by-products of tobacco combustion such as tar constituents and carbon monoxide rather than nicotine. It is of interest to determine if there is an opportunity to develop a dry powder form of inhalable nicotine formulation and assess its applicability in reducing the health problems associated with smoking. The pulmonary route of nicotine delivery would be expected to mimic the effects of tobacco smoking and would significantly reduce cravings and withdrawal symptoms. Wang et al. [192] built a nose-only inhalation device for pulmonary administration of nicotine to mice and determined the optimal operational parameters. They used the locomotor activity test to compare the effects of the inhaled nicotine hydrogen tartrate-loaded chitosan nanoparticles (NHT-CS NPs) with an inhaled and subcutaneously injected NHT in C57BL/6 mice. A minimum of 0.88 mg inhaled of NHT-CS NPs or 0.59 mg inhaled of NHT was required to alter locomotor activity similarly to injection of 0.50 mg/kg nicotine, suggesting the reformulation process did not alter the activity of NHT-CS NPs. No differences between untreated and NHT-CS NPs treated lung tissue upon histological examination were observed. The results indicated the inhaled NHT-CS NPs represent a viable preclinical option for developing novel inhalation formulations as a potential anti-smoking therapeutic.

#### 3.4.5. Buccal Drug Delivery

**Figure 30:**
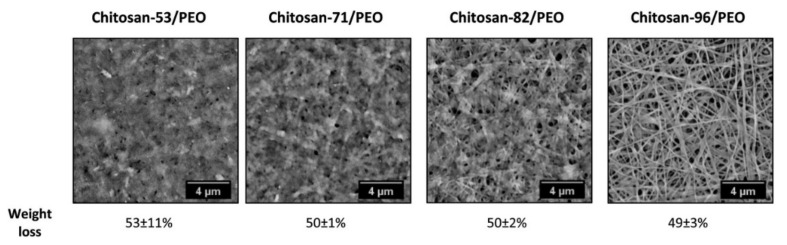
Aqueous stability of CS/PEO nanofibers electrospun with CS of various DDA. Representative SEM images (dry) and weight loss of CS/PEO nanofibers electrospun with CS of various DDA (53%, 71%, 82%, or 96%) after exposure to water for 3 h. 10,000× magnification [230]. Reproduced with permission from Elsevier (Copyright©2020).

Buccal drug delivery has been used as an alternative for the delivery of drugs that undergo the first-pass metabolism or are susceptible to pH and enzymatic degradation (occurring in the gastrointestinal tract). This kind of delivery increases the drug bioavailability and reduces the number of drugs required. In addition, through this route, the drugs of high molecular weight can be delivered which cannot be administered by the oral route. Ideally, a buccal drug shows controlled drug release and a prolonged time of residence in the oral cavity [52]. However, the drug concentration absorbed in the buccal mucosa is often low to obtain an acceptable therapeutic effect, mainly due to the saliva turnover, tongue and masticatory movements, phonation, enzymatic degradation, and lack of epithelium permeation. Therefore, the encapsulation of drugs into NPs is an important strategy to avoid such problems and improve their buccal delivery. Different materials from lipids to natural or synthetic polymers and others have been used to protect and deliver drugs in a sustained, controlled, or targeted manner, and enhance their uptake through the buccal mucosa improving their bioavailability and therapeutic outcome [229]. Mucoadhesive CS-based electrospun nanofibers have been reported to be promising candidates for overcoming challenges associated with sublingual drug delivery. For example, Stie et al. [198] focused on the elucidation of the mucoadhesive properties of chitosan/polyethylene oxide (PEO) nanofibers. They studied how the degree of deacetylation (DDA, 53–96%) of CS influenced their morphological and mucoadhesive properties. The representative SEM images (dry) and weight loss of CS/PEO nanofibers electrospun with CS of various DDA (53%, 71%, 82%, or 96%) after exposure to water for 3 h are shown in Figure 30. The mechanism of mucoadhesion was explained by the intermolecular interactions of CS with mucin from bovine submaxillary glands using quartz-crystal microbalance with dissipation monitoring and by adhesion of the nanofibers to ex vivo porcine sublingual mucosa. An increase in CS DDA improved the morphological stability of the nanofibers in water but did not contribute to altered mucoadhesive properties.

Because of the very low oral bioavailability of insulin, transmucosal delivery routes have gained much attraction. Limiting factors (swallowing of the delivery device, mucus turnover, and dilution of drug with saliva) can be overcome using polymers with high mucoadhesiveness such as CS and CS-derived nanoparticulate buccal systems [197]. Such mucoadhesive drug delivery systems facilitate the release of insulin for a prolonged period at the site of application, and thereby maintain a high drug concentration gradient across the tissue, improving the total drug permeation through the mucosa. Encapsulation of insulin in innovative drug delivery systems such as NPs may also further protect the drug from degradation and washout by saliva, as well as control the drug release kinetics. 

Improved mucoadhesive properties of thiolated polymers, such as improved tensile strength, high cohesive properties, rapid swelling, and water uptake behavior, have made them an attractive new generation of bioadhesive polymers. It has been proven that thiolation of CS results in the promotion of mucoadhesion and permeation enhancing effect of CS. Rahbarian et al. [197] synthesized and characterized insulin NPs composed of N-triethyl chitosan thiolated with L-Cysteine (CysTEC) as a new generation of thiolated CS derivatives. The preparation of the NPs via a PEC method was optimized using the Box-Behnken experimental design methodology leading to smaller particle size and polydispersity index (PdI) as well as higher zeta potential and entrapment efficiency. The in vitro and ex vivo study results demonstrated that insulin NPs composed of CysTEC had excellent permeation from buccal mucosa. However, the authors suggested additional in vivo experiments are needed to further confirm these findings.

Tumors located in the oral mucosa are challenging to treat via buccal drug delivery since surgery can lead to aesthetic, speech, and salivation problems, radiotherapy alone is often ineffective, and systemic chemotherapy brings meaningful side effects to the patient [230]. Local administration of an anticancer agent to the oral cavity could enable the precise delivery of cytotoxic drugs to the tumor site. In this sense, polymeric NPs with mucoadhesive properties could be an adequate choice to entrap and deliver a chemotherapeutic agent [231]. As a matrix to prepare these NPs, CS stands out as a biocompatible and biodegradable amino polysaccharide that can provide mucoadhesiveness to drug delivery systems. 

Matos et al. [230] developed mucoadhesive CS NPs entrapping the chemotherapeutic oxaliplatin (OXPt) and evaluated ex vivo its penetration in porcine mucosa under both passive and iontophoretic topical treatments. These NPs provided a “burst effect” on the drug release followed by a longer-term controlled release. When applied to the oral mucosa, the CS NPs increased 3-fold drug penetration, and this rate was maintained even when the mucosa was “washed” with a buffer to mimic salivation. Iontophoresis doubled the amount of OXPt transported to the mucosa. These amounts exceeded the dose required to cause cell death of an oral tumor cell line. Besides, CS NPs increased the rate of cells that entered apoptosis. This study points to the feasibility of topical therapy with CS NPs, potentialized by the application of iontophoresis, to treat oral tumors.

Pornpitchanarong et al. [231] developed catechol (Cat)-modified chitosan/hyaluronic acid (HA) nanoparticles (Cat/CS/HA NPs) as a new carrier to deliver doxorubicin (DOX) to oral cancer cells. The Cat moiety of the NPs allowed the excellent adhesion of the carrier to the oral mucosa and sustained local delivery of DOX into the oral cavity. The modified NPs demonstrated superior mucoadhesive capability on ex vivo porcine oral mucosal tissues compared with the unmodified NPs. The DOX-loaded Cat/CS/HA NPs (DOX-Cat/CS/HA NPs) inhibited the growth of the HN22 oral squamous cell carcinoma cell line with a low IC50. Moreover, the DOX-Cat/CS/HA NPs were taken up, accumulated, and induced apoptosis in cells more extensively compared with free DOX. 

#### 3.4.6. Periodontal Drug Delivery

Periodontal diseases are chronic infectious diseases and are major oral health problems [232]. They are caused by a dental plaque biofilm in the oral cavity that can lead to the progressive destruction of periodontal tissue, including the gingiva, periodontal ligament (PDL), cementum, and alveolar bone. Different approaches for treating the periodontal tissues damaged by periodontitis have been pursued including the so-called guided tissue regeneration approach (GTR). The GTR treatment involves the application of a barrier membrane between the gingival epithelium and connective tissues, which prevents migration of the epithelium and gingival connective tissue cells into the defect sites and provides a secluded space for the formation of PDL, cementum, and bone. The administration of antimicrobial agents prevents and treats bacterial periodontal infections. Controlled local delivery of antimicrobials into the defect sites presents many advantages compared to oral administration, including site-specific delivery, a decrease in dose requirement, and side effects [199]. CS has attracted considerable attention for this area owing to its special properties including antimicrobial efficacy, biodegradability, biocompatibility, and non-toxicity. It also has the propensity to act as a hydrating agent and display tissue healing and osteoinductive effect [232].

The new approach is to develop CS-based GTR membranes loaded with antibiotics for local drug release. Dos Santos et al. [200] prepared core-sheath nanofibers via coaxial electrospinning by using CS with well-defined structural characteristics as the shell layer and poly(vinyl alcohol) (PVA) containing tetracycline hydrochloride (TH) as the core layer. Defect-free and geometrically uniform nanofibers with diameters predominantly in the range of 100–300 nm were prepared. The mechanical properties as well as the stability of nonwovens in an aqueous medium were greatly improved by genipin-crosslinking, which enabled a sustained release of TH over 14 days. Results also revealed that the release profile of TH in the presence of lysozyme was affected by the composition of the shell layer, as the TH release rate increased with decreasing the degree of deacetylation (DD). Further in vitro antimicrobial activity experiments demonstrated that the cross-linked nonwovens containing TH had strong activity against bacterial strains associated with periodontal disease. Additionally, the nonwovens did not exhibit any significant cytotoxicity toward fibroblast (HDFn) cells, hence, showing their potential for applications as a novel drug delivery platform for periodontitis treatment.

Another approach is to use metallic NPs possessing antimicrobial effectiveness in conjugation with polymeric NPs leading to periodontal diseases treatment of low toxicity. Araujo et al. [126] evaluated the antimicrobial and antibiofilm effects of colloidal nanocarrier iron oxide nanoparticles (IO NPs) coated with chitosan for chlorhexidine (CHX) on *Candida glabrata* and *Enterococcus faecalis*. They also tested the cytotoxic effect of this formulation on murine fibroblasts. CHX has improved the antibiofilm effect and reduced the cytotoxicity (at low concentrations) when conjugated to CS-coated IO NPs. This new colloidal formulation has the potential as an alternative antimicrobial agent to pure CHX for the control of biofilm-related oral diseases, such as oral candidiasis and endodontic infections.

#### 3.4.7. Dermal and Transdermal Drug Delivery

##### Transdermal Drug Delivery

A transdermal drug delivery system is being developed to vanquish the downsides associated with conventional administration routes as it avoids first-pass hepatic metabolism, prevents the intervention of intestinal and gastric fluids, improves patient compliance, enables easy termination of therapy, gives a possibility of self-administration, etc. The main challenge in designing transdermal dosage forms is to overcome the low permeability of the skin. Several strategies have been developed to overcome the barrier properties and to enhance the transportation of drug molecules across the skin. In the last decades, numerous transdermal patches fabricated from polysaccharides have been reported. There has been a growing interest in using CS in transdermal formulations thanks to their biocompatibility, non-toxicity, film-forming ability, non-skin irritancy, etc. [233]. NPs have been advocated as one of the potential delivery systems which can resolve the skin permeation limitation of drugs to a great extent. Polysaccharide NPs are met with rising popularity as a transdermal drug delivery system. The CS NPs are biodegradable, biocompatible, mucoadhesive, and possess mucosal permeation enhancement properties. They can interact with the skin mucosa and fluidize the epidermal lipid and protein domains to promote transdermal drug diffusion. They also can have a synergistic therapeutic contribution for local disorders such as skin malignant melanoma and infection or systemic complications such as diabetes and hyperlipidemia [205].

Microwave radiation (characterized by frequencies range between 300 MHz and 300 GHz) is envisaged to mediate transdermal drug delivery through exerting the spacing of lipid architecture of stratum corneum into structureless domains in a non-thermal fashion. Nawaz et al. [205] investigated transdermal drug delivery mechanisms of CS NPs with free or conjugated 5-fluorouracil with the synergistic action of microwave in skin modification. Their transdermal drug delivery profiles across untreated and microwave-treated skins were examined. Both constituent materials of NPs and drug encapsulation were required to succeed in transdermal drug delivery. The drug–NPs transport was facilitated through constituent NPs and microwave fluidizing protein/lipid domains of epidermis and dermis and dermal trans-to-gauche lipid conformational changes. The microwave-induced marked changes to the skin ceramide content homogeneity. The CS NPs largely affected the palmitic acid and keratin domains.

**Figure 31:**
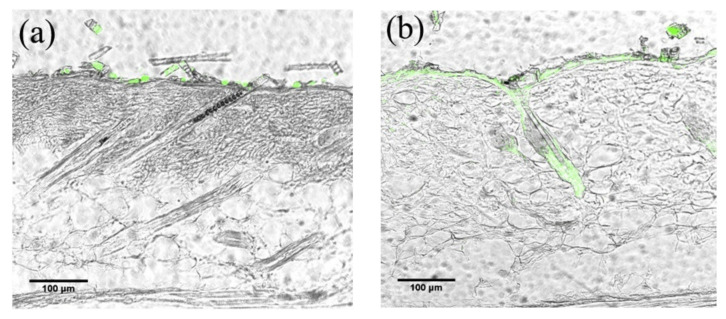
Fluorescence microscopy images of cross-sections of mouse skin without iontophoresis (**a**) and with iontophoresis (**b**) using fluorescein isothiocyanate-HEL-loaded nanoparticles [203]. Reproduced with permission from Elsevier (Copyright©2020).

The skin is also one of the most promising routes of administration for allergen immunotherapy due to its high prevalence of activated antigen-presenting cells (APCs) with immunostimulatory and migratory capacities. In vaccination, dermal cells residing in and around hair follicles are thought to be more accessible to topically applied vaccines than dermal cells in the interfollicular epidermis. Thus, it is important to deliver antigenic proteins to hair follicles to induce immune responses. Takeuchi et al. [203] investigated transdermal administration of poly(DL-lactide-co-glycolide) (PLGA) NPs coated with chitosan hydroxypropyltrimonium chloride using iontophoresis for efficient drug delivery to hair follicles as a possibility of using it in allergen immunotherapy. Hen egg-white lysozyme (HEL) was used as a model antigen. The results of an uptake test into dendritic cells showed that the usage of the NP carrier increased the mean fluorescence intensity by 5.6 times. An ex vivo skin accumulation study showed that when iontophoresis was applied to the NPs, the HEL concentration in mouse skin was 9.6 times higher than that without iontophoresis and 2.1 times higher than that of the HEL solution. Fluorescence microscopy images of cross-sections of mouse skin (illustrated in Figure 31) confirmed that HEL was efficiently delivered to hair follicles using iontophoresis and NP carriers. In vivo percutaneous immunoreactivity study revealed that ten weeks after the initiation of the test using iontophoresis and the NP carrier, HEL-specific IgG1, and IgG2a titers were higher than those obtained by subcutaneous injection of the HEL solution. This indicated the effectiveness of the combined use of iontophoresis and nanoparticle carriers for transdermal delivery of antigens to hair follicles.

Applications of nanofiber-based fabrications are growing in a variety of fields. Nanofiber-based carriers for localized delivery of chemotherapeutic agents have some additional advantages, like stability boosting of loaded chemotherapeutic agents with the maintenance of its cytotoxic property, controlled and targeted tumor delivery, low systemic side effects, and toxicity, single-dose therapy, and reducing administered dose in the formulation. The unique properties of CS-based nanofibers provide a great potential to formulate an effective topical drug delivery system. Morad et al. [204] formulated a nanofiber-based topical drug delivery system for colchicine. Colchicine systemic toxicity is an obstacle against the administration of systemic chemotherapy for melanoma management. Significant colchicine deposition in the skin with remarkable cytotoxicity against melanoma cell line makes it a desirable formulation to be administered as a topical or local reservoir system for neoadjuvant chemotherapy before other interventions and adjuvant therapy of tumors after surgery and, also, for other skin diseases with dose adjustment. Besides, the observed first-order release kinetic behavior through the skin could suggest the developed CS-based biocomposite nanofibers as a transdermal colchicine delivery system which improves its efficiency in systemic indications.

##### Dermal Delivery

Topical medication can avoid systemic side effects linked with conventional oral and injection administration. It can also reach the disease site through the mucous membrane and skin penetration more quickly and directly [11].

NPs are considered advantageous for the treatment of acne since they provide a solution to the low dermal bioavailability of medications and allow for their controlled release. Among the promising cosmeceuticals/nutraceuticals recently used for acne treatment, is nicotinamide. This drug exhibits anti-inflammatory properties and is reported to decrease sebum production. Abd-Allah et al. [201] prepared CS NPs and loaded the nutraceutical nicotinamide. The NPs were optimized, characterized, and clinically tested on patients suffering from acne vulgaris. The topical merits of the CS NPs were proven by their strong skin adhesion ex vivo and high nicotinamide deposition in the different skin layers (stratum corneum, epidermis, and dermis) amounting to a total of 68%. When clinically tested on patients, the nicotinamide CS NPs displayed a 73% reduction in the inflammatory acne lesions compared to untreated areas, hence proving that this delivery system can be a clinically sounding option for the treatment of skin diseases.

Targeted hair follicle topical treatment is a promising alternative for androgenic alopecia using dutasteride (DUT). It clinically appears to provide better efficacy compared to finasteride but presents sexual dysfunctions related to adverse reactions when orally administered. DUT is an extremely lipophilic substance with poor aqueous solubility. It makes this molecule troublesome to be incorporated in conventional topical drug delivery systems intended for the scalp application, in which a viscous or oily residual could give an undesirable hair aspect and be rejected by the patients. Nanoscale drug delivery systems would be ideal in this case, as they could incorporate the drug in their interior but still be administered in an aqueous formulation. Furthermore, nanosystems present a natural tendency for accommodation into the follicular cavities. Ushirobira et al. [202] compared two strategies to further promote DUT follicular-targeted delivery: the chemical modulation of nanosystem surface properties by coating with the natural polymer CS, and the application of a massage. For this, poly-(ε-caprolactone)-lipid-core nanocapsules (NCs) containing DUT were developed and had their permeation profile compared to CS-coated nanocapsules (CS NCs). Both coated and non-coated NCs targeted the hair follicles compared to a drug solution. Enhanced hair follicles targeting was observed after the massage procedure, with 5- and 2-fold increases relative to the non-coated NCs and coated CS NCs, respectively. Therefore, DUT in NCs can target the follicular casts, and a simple physical stimulation can additionally enhance the drug amount accumulated.

#### 3.4.8. Wound Healing

In recent years, a growing deal of attention has been attracted to develop and promoting wound healing agents. Injured skin can be infected and colonization by a diverse population of microbes, which can facilitate their access to the underlying tissues. Infection is considered a significant factor that prolongs the wound healing process. Wound dressing should protect the wound from microorganisms, provide a moist environment to hinder the wound dryness, decline the wound surface necrosis, be oxygen permeable without dehydrating the wound, plus should be comfortable, and avoid mechanical trauma. Moreover, low toxicity, biodegradability, and biocompatibility are some remarkable criteria for a material employed for the fabrication of wound dressing. It is proved that N-acetyl glucosamine as a monomer unit of CS stimulates hemostasis, encourages cell proliferation, and subsequently accelerates wound healing. As a biocompatibility aspect, CS does not lead to adverse reactions in contact with human cells. Furthermore, by binding to red blood cells CS causes rapid blood clotting. CS is also utilized to improve the stability of the drugs by producing CS NPs and resulting in drug accumulation increment [206]. 

Fahimirad et al. [206] fabricated the electrospun poly(ε-caprolactone) (PCL)/Chitosan (CS)/curcumin (CUR) nanofiber (CUR-PCL/CS NFs) with CUR loaded CS nano-encapsulated particles (CUR-CS NPs). The electrospraying of CUR-CS NPs on the surface of CUR-PCL/CS NFs resulted in enhanced antibacterial, antioxidant, cell proliferation efficiencies, and higher swelling and water vapor transition rates. In vivo examination and histological analysis showed CUR-PCL/CS NFs electrosprayed with CUR-PCL/CS NFs led to a significant improvement of the complete well-organized wound healing process in MRSA (methicillin-resistant *Staphylococcus aureus*) infected wounds. These results suggest that the application of CUR-PCL/CS NFs electrosprayed with CUR-CS NPs as a wound dressing significantly facilitates wound healing with notable antibacterial, antioxidant, and cell proliferation properties.

Antibiotic-loaded nanofibrous-delivery systems offer an advanced approach to overcome several limitations associated with antibiotic therapy; the benefits include high surface-area-to-volume ratios, high porosity, high loading capacity, high encapsulation efficiency, minimum systemic toxicity, and the possibility of controlling the release from immediate to controlled release. Antibiotic-loaded NFs can be applied topically for skin and wound healing, post-operation implants for the prevention of abdominal adhesion, and prophylaxis and treatment of infections in orthopedic surgery. Amiri et al. [67] developed a local antibiotic delivery system using polyethylene oxide/chitosan nanofibers (PEO/CS NFs) prepared via electrospinning for delivery of teicoplanin. The PEO/CS NFs were able to release teicoplanin for up to 12 days. An antibacterial test and time-kill study on *Staphylococcus aureus* also demonstrated that loading teicoplanin in the PEO/CS NFs not only kept the antibacterial activity of antibiotic but also enhanced it up to 1.5- to 2-fold. The teicoplanin-loaded NFs did not show any cytotoxicity to human fibroblast. Moreover, in vivo study on a rat full-thickness wound model confirmed the safety and efficacy of applying the teicoplanin-loaded PEO/CS NFs; a significant improvement in wound closure was observed especially with the NFs containing 4% teicoplanin. The sustained release profile, enhanced drug activity, cytocompatibility, and significant wound healing activity affirm the potential applications of the teicoplanin-loaded PEO/CS NFs in wound healing and local antibiotic delivery.

Wounds are often recalcitrant to traditional wound dressings and a bioactive and biodegradable wound dressing using hydrogel membranes can be a promising approach for wound healing applications. Shafique et al. [207] designed hydrogel membranes based on hyaluronic acid, pullulan, and polyvinyl alcohol and loaded with chitosan-based cefepime nanoparticles (CEF-CS NPs) for potential use in cutaneous wound healing. The results indicated the novel crosslinking and thermal stability of the fabricated hydrogel membrane. The in vitro analysis demonstrated that the developed membrane had water vapors transmission rate (WVTR) between 2000 and 2500 g/m^2^/day and oxygen permeability between 7 and 14 mg/L, which laid in the range of an ideal dressing. The swelling capacity and surface porosity to liberate encapsulated drug (CEF) in a sustained manner and 88% of drug release was observed. The CEF-loaded hydrogel membrane demonstrated a higher zone of inhibition against *Staphylococcus aureus*, *Pseudomonas aeruginosa*, and *Escherichia coli* and the excisional rat model exhibited an expeditious recovery rate. The developed hydrogel membrane loaded with CEF-CS NPs is a promising approach for topical application and has a great potential for an accelerated wound healing process.

**Figure 32:**
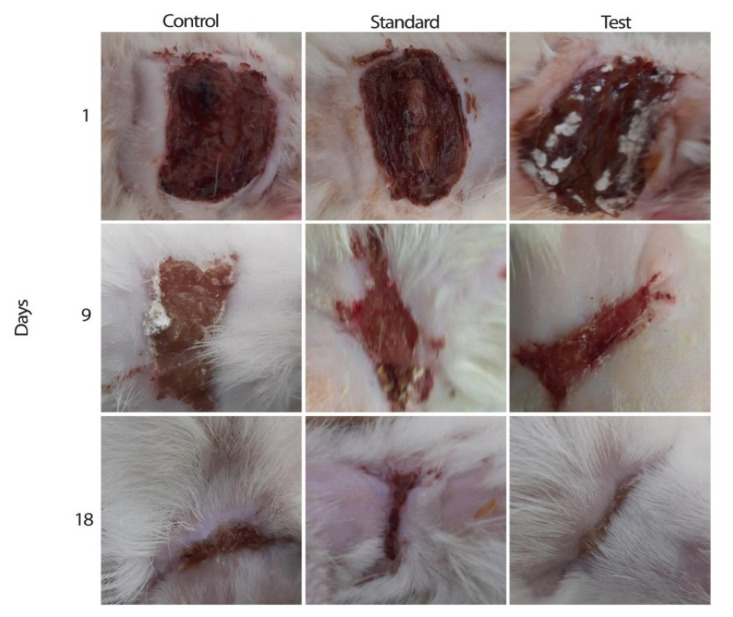
Photographs of wound areas of the diabetic group of rats on 1, 9, and 18th days after treatment with Control, Standard, and PM-CS NPs H-1 (test treated) [208]. Reproduced with permission from Elsevier (Copyright©2020).

The application of modern nanomedicines to enhance wound healing is growing due to their simplicity for topical organization and fast flexibility with molecules that can boost and reinforce the process of healing even in patients with diabetes. Manne et al. [208] prepared an effective *Pterocarpus marsupium* heartwood extract-chitosan nanoparticles (PMH-CS NPs) loaded carbopol hydrogel and evaluated its drug release efficiency, in vitro antimicrobial activity, and wound healing action in streptozotocin administered diabetic rat models. In vivo rats treated with such hydrogel displayed significantly much quicker healing of wounds in both diabetic and non-diabetic rats. Photographs of wound areas of the diabetic group of rats on 1, 9, and 18th day after treatment with Control, Standard, and PM-CS NPs H-1 (test treated) are shown in Figure 32. The histological examination revealed re-epithelization and growth of granular tissue and an improvement in collagen deposition. These results indicated the effectiveness of optimized CS nanocomposites as a potential treatment for curing diabetic wounds.

#### 3.4.9. Vaginal Drug Delivery

Vaginal delivery is very attractive for both local and systemic administration of drugs. For the latest purpose, it shows several advantages concerning conventional oral or parenteral ways, such as the avoidance of the stomach acidic pH, the hepatic first-pass effect, or the needle-based formulations uncomfortable for the patients. The vaginal mucosa is characterized by high robustness, ease of accessibility, and rich blood supply. The effectiveness of typical vaginal formulations (creams, foams, gels, tablets, films, rings, and suppositories) can be limited by their low active residence time due to the washing-effect of the vaginal physiological fluids, small absorption area, barrier properties of the mucosa, and inadequate spreading of the formulation on vaginal surfaces. Pharmaceutical nanocarriers provide several advantages such as a high surface area and great carrier capacity, improved stability of the therapeutic agents against chemical/enzymatic degradation, enhanced bioavailability, longer drug effect in the target tissue, and drug targeting upon inclusion of specific ligands. The development of NP-based vaginal drug delivery formulations has largely been focused on biological vaccine or microbicide delivery for prevention or treatment of sexually transmitted diseases such as human immunodeficiency virus (HIV), herpes simplex virus (HSV), or human papillomavirus (HPV). The vaginal route allows a localized delivery of peptide-based vaccines/microbicides close to both the site of infection and infectible cells. An opportune vaginal drug delivery system should provide mucosal interactions that facilitate bioadhesion with mucosa increasing drug residence time at the mucosal surface, and penetration enhancement properties to allow penetration into vaginal tissue cells. In the last years, many authors have studied the mucoadhesive and penetration enhancement properties of CS in this area [11,52]. 

Marciello et al. [210] proposed a straightforward and efficient strategy for the vaginal application and release of peptide-loaded mucoadhesive CS NPs. The CS NPs, responsible for carrying the peptide drug and allowing adhesion to the vaginal mucosal epithelium, were encapsulated in suitable hydrophilic freeze-dried cylinders. The hydrophilic freeze-dried cylinders facilitated the application and quick release of the CS NPs to the vaginal zone. Upon contact with the aqueous vaginal medium, the excipients constituting these sponge-like systems were quickly dissolved enabling the release of their content. In vitro release studies showed the ability of the sponge-like systems and the CS NPs to deliver the mucoadhesive NPs and peptides, respectively. CLSM (confocal laser scanning microscopy) micrographs proved the CS NPs ability to promote the peptide penetration inside the vaginal mucosa.

To prime adaptive immune responses from the female reproductive tract (FRT), particulate antigens must be transported to draining lymph nodes (dLNs) since no local organized lymphoid structures are being equivalent to those found in the respiratory or gastrointestinal tracts. Therefore, it is a challenge to find how to safely and effectively navigate successive barriers to transport such as crossing the epithelium and gaining access to migratory cells and lymphatic drainage that provide entry into dLNs. Park et al. [234] demonstrated that an intravaginal pretreatment with CS significantly facilitated the translocation of NPs across the multilayered vaginal epithelium to target dLNs. In addition, the CS pretreatment was found to enhance the NP associations with immunogenic antigen-presenting cells in the vaginal submucosa. These observations indicate that CS may have great potential as an adjuvant for both local and systemic protective immunity against viral infections in the FRT.

Vaginal candidiasis is a common genital tract infection caused by dimorphic fungi of the genus Candida. Sustained delivery systems prepared using biodegradable polymers, such as CS, are interesting because they may promote a slow release of the drug by controlled rates of their degradation. CS itself can reduce the microbial population. Hence, a nanoparticulate system based on CS can represent an alternative and promising therapy for the treatment of vulvovaginal candidiasis. Amaral et al. [209] fabricated polymeric NPs based on CS incorporating the common antifungal miconazole nitrate and tested them in vivo using murine vulvovaginal candidiasis (VVC). The treatment using the CS NPs with miconazole nitrate provided the same therapeutic efficacy as miconazole nitrate in a commercial cream formulation but using the antifungal content about seven-fold lower. In another work, Arumugam et al. [211] studied the killing effects of seaweed-derived metabolite Callophycin A (Cal) loaded in both chitosan (Cal-CS) and marine sponge-derived spicules (Cal-Spi) NPs. Vaginal candidiasis-induced animal model experiments confirmed that the candidicidal activity of Cal-CS NPs resulted in a significant reduction in the fungal burden of vaginal lavage. The histo-morphological alterations also evidenced that the protective role of Cal-CS NPs in the VVC model. The Cal-CS NPs could be used as an alternative strategy for the development of novel marine natural product-based topical applications.

#### 3.4.10. Vaccine Delivery

Vaccine refers to biological products applied to prevent or control the occurrence and spread of infectious diseases. As for nature, the vaccines may be microbes or their toxins, enzymes, human or animal serum, and cells. The emergence of vaccines has made a significant contribution to the prevention and control of diseases. However, many problems are affecting the quality of the vaccines during their preparation, storage, and administration The major hurdle associated with oral mucosal immunization is enzymatic degradation of antigen at the stomach and low uptake of antigen sampling cells through the intestine. Hence, the vaccine administered through the oral route (less through the nasal route) requires the use of NPs. Nanomaterial-encapsulated vaccines have been proved effective in the delivery of antigens to immune cells. Such encapsulation can help in promoting immune responses and represents a promising vaccine transport vehicle. In oral vaccine delivery, the main target is Peyer’s patches. Due to the nanoparticle system, the vaccine is protected from enzymatic degradation while going to the mucosal tissue and taken up by the M-cells. CS is a particularly attractive choice for vaccine delivery because of its low immunogenicity, low toxicity, biocompatibility, and biodegradability. It has been widely used for mucosal but also systemic vaccine delivery and even in the preparation of DNA mucosal vaccines [11,52]. For novel, recently published oral and nasal CS NP-based vaccine delivery systems see Section Oral Vaccines and Section Systemic Nasal Delivery, respectively.

In systemic vaccine delivery, CS acts as an adjuvant. Activation of macrophages occurs after the uptake of CS [52]. CS exhibited good tolerability and excellent immune stimulation. The numerous recent hepatitis A outbreaks emphasize the need for vaccination; despite the effectiveness of the current vaccination, further development is needed to overcome its high cost plus some immune response limitations. AbdelaAllah et al. [212] evaluated the use of CS and ALG-coated CS NPs as an adjuvant/carrier for the hepatitis A (HA) vaccine against the traditional adjuvant alum (AL). Immune responses towards the HA-AL, HA-CS, and HA-ALG/CS NPs were assessed in mice. The HA-ALG/CS NPs significantly improved the immunogenicity by increasing the seroconversion rate, the hepatitis A antibodies level, and the splenocytes proliferation. Thus, the HA-ALG/CS NPs adjuvant was superior to the other classes in IFN-γ (interferon-gamma) and IL-10 (interleukin 10) development. The solution formula of the HA vaccine with CS showed comparable humoral and cellular immune responses to AL-adjuvanted suspension with a balanced Th1/Th2 immune pathway. The study demonstrated the potential of the ALG-coated CS NPs as an effective carrier for an HA vaccine. 

#### 3.4.11. Gene Delivery

Genes encoded specific proteins are essential for various physiological processes of the body and their mutations often result in disease. Gene therapy is a promising strategy to treat genetic diseases via the introduction of a foreign gene into a target cell which is then transcribed, and the genetic information is finally translated into the corresponding protein. The aim is to correct a damaged gene. For this process to be completed, the gene delivery system has to overcome several hurdles. The factors affecting transfection include targeting the delivery system to the target cell, uptake, and degradation in the endolysosomes, transport through the cell membrane, and intracellular transfer of plasmid DNA to the nucleus. Thus, DNA and RNA molecules can be destroyed by harsh acids and enzymes that are produced in the body. DNA and RNA are anionic polymers that have a good affinity with cationic polymers such as CS. CS can protect the DNA or RNA against nuclease degradation by forming a polyelectrolyte complex with negatively charged DNA or RNA. This protection can improve the transfection efficiency [11,52].

There are two main approaches for gene delivery systems, i.e., viral and nonviral ones. Considering efficiency and safety issues, polymeric vehicles would seem more convenient than other gene delivery approaches. Because of its positive charge and the small size of CS NPs (below 100 nm), endocytosis can be achieved. CS NP is therefore a promising excipient for non-viral gene delivery. In addition, chitosan–DNA-based drug complexes protect at least to some extent against degradation by DNAses in this way improving the bioavailability of DNA-based drugs delivered into the body [8]. Rahmani et al. [216] investigated in vitro DNA transfection efficiency of three CS derivatives, namely trimethyl chitosan thiolated with cysteine (CysTMC), methylated 4-N,N-dimethyl aminobenzyl N,O carboxymethyl chitosan (MABCMC), and trimethyl aminobenzyl chitosan thiolated with cysteine (CysMABC). The results showed that all the polymers could condense DNA plasmid strongly from N/P 2 and nanocomplexes had eligible sizes and zeta potentials. Moreover, the nanocomplexes had negligible cytotoxicity and CysMABC was the most effective vehicle for gene delivery in HEK-293T cells. In the two other cell lines, SKOV-3 and MCF-7, CysTMC exhibited the highest transfection efficiency.

Among several types of non-viral vectors, cell-penetrating peptides (CPPs), short peptides with 30 amino acids, are promising. CPPs show high biocompatibility and offer the potential for large-scale production. However, CPPs exhibit low transfection efficiency. Hybrid conjugation of CPPs with inorganic nanomaterials improved their efficiency and may open new venues for multifunctional treatment. Abdelhamid et al. [214] synthesized hierarchical mesoporous carbon (MPC) nanomaterials derived from the carbonized chitosan (CTS) encapsulated zeolitic imidazolate frameworks-8 (ZIF-8) and applied them for gene delivery. The MPC materials were applied as a non-viral vector for gene delivery using two oligonucleotides (ONs) called luciferase-expressing plasmid (pGL3), and splice correction oligonucleotides (SCO). The materials were biocompatible and showed insignificant toxicity. The MPC improved the transfection efficiency of cell-penetrating peptides (CPPs) (PepFect 14 (PF-14), and PF-221) by 10-fold compared to commercial vector Lipofectamine™2000 due to the synergistic effect of their action. This may be due to the positive charge of CPPs that ensure higher interactions with the negative charge of the cells.

Small interfering RNA (siRNA) is a double-stranded RNA of 20 to 25 nucleotides in length and has many important functions in biology. It primarily plays a unique role in the RNA interference events which regulates gene expression in a specific manner. siRNA-based therapies have great potential in the modulation of a large number of target mRNA molecule silencing, which ultimately decreased levels of the targeted protein. Nevertheless, there are challenges that applications of this gene silencing technology need to overcome, including rapid degradation of siRNA under physiological conditions and the difficulty of passing the cytoplasmic membrane of negatively charged siRNA. The key challenge in realizing the therapeutic potency of siRNA is the development and design of a novel degradable vector with safe and sufficient gene delivery efficacy. To improve the transfection efficiency, new safe vehicles for cancer gene delivery based on NPs have been developed. Recently some different modifications of CS have been used to produce these NPs, for example, a polyelectrolyte complex containing trimethyl chitosan (TMC) as the positive and, dextran sulfate, and alginate as the negative part [218], guanidinylated O-carboxymethyl chitosan (GOCMCS) along with poly-β-amino ester (PBAE) for siRNA delivery [220].

Yan et al. [219] fabricated the NPs of carboxymethyl chitosan (CMC) and labeled fluorescein isothiocyanate (FITC)-chitosan hydrochloride (FITCCS) as carriers for ultrasound-triggered drug delivery to treat colon cancer. The results showed the FITCCS/CMC NPs could effectively encapsulate anti-β-catenin siRNA through ionic gelation self-assembly to improve the stability of siRNA. The FITCCS/CMC NP-based pH-sensitive delivery system provided a controlled release of siRNA through responding to external stimulus (ultrasound) under favorable pH conditions. Following the transfection of anti-β-catenin siRNA for 48 h, the β-catenin protein expression of the colon cancer cells was reduced to about 40.10%, indicating the effective reduction of the protein that promotes colon cancer proliferation. 

CS as a promising polysaccharide for gene/siRNA delivery requires some additional treatments to modify CS NPs. Mobarakeh et al. [217] modified CS NPs to introduce anti-HIV siRNA into two mammalian cell lines, macrophage RAW264.7, and HEK293. The CS NPs were prepared by using different concentrations of CS, polyethyleneimine (PEI), and carboxymethyl dextran (CMD) in various formulations. The results indicated that the combination of CS with both CMD and PEI significantly improved both cell viability and siRNA delivery. In the studied cell types, the NPs noticeably increased siRNA delivery efficiency with no significant cytotoxicity or apoptosis-inducing effects compared to the control cells. In addition, the NPs significantly reduced the RNA and protein expression of HIV-1 tat in both stable cells.

**Figure 33:**
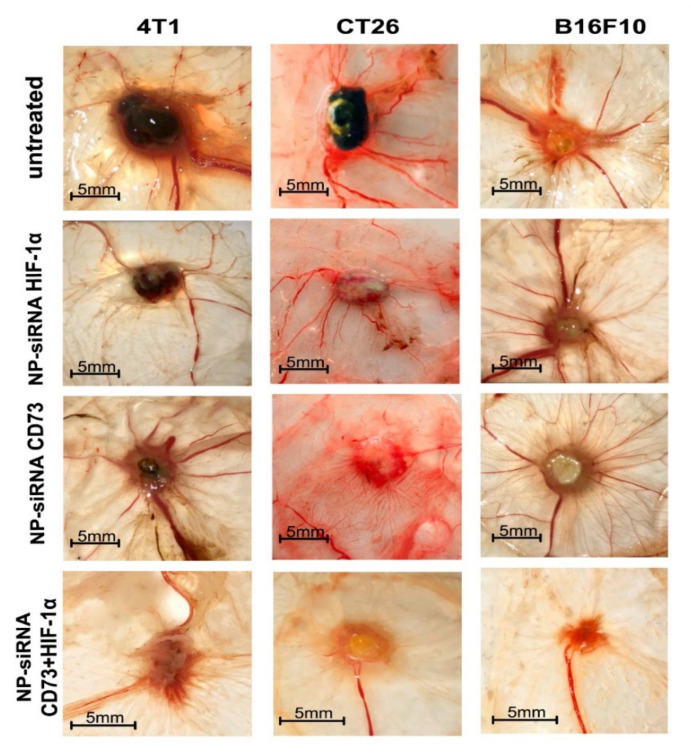
CAM assay of HIF-1α/CD73 siRNA loaded TAT-TMC/TC/SPION nanocarriers suppressed in vivo tumor development and angiogenesis [221]. Reproduced with permission from Elsevier (Copyright©2020).

Induction of Hypoxia Inducible Factor (HIF) as a direct consequence of oxygen deficiency in tumor tissues is a potent stimulus of CD73 (ecto-5′nucleotidase) expression. Hypoxic environment and CD73 overexpression are associated with altered metabolism, elevated cancer cell proliferation, and tumor vascularization. Hajizadeh et al. [221] developed a delivery system for silencing CD73 and HIF-1α gene using siRNA-loaded superparamagnetic iron oxide (SPION) nanocarriers for cancer treatment. The SPIONs were encapsulated with thiolated chitosan (TC) and trimethyl chitosan (TMC) for improving their stabilization and functionalization. The produced NPs were efficiently accumulated in the tumor site, indicating their stability and targeting ability in reaching the tumor region. TAT-conjugated TMC/TC/SPIONs containing siRNAs significantly reduced the HIF-1α and CD73 expression levels in cancer cells. Moreover, siRNA-loaded NPs effectively reduced tumor growth and angiogenesis as is illustrated by CAM (cell angiogenesis) assay in Figure 33. Hence, the co-silencing of CD73 and HIF-1α can be assumed as a novel anti-cancer treatment strategy with a high tumor suppression potential.

Some researchers focused on the development of delivery systems that combine silencing of inhibitor of apoptosis (IAP) genes BV6 (their increased expression is associated with cancer progression and chemoresistance) and interleukin (IL)-6, as a new promising anti-tumor treatment strategy. For this purpose, Salimifar et al. [222] prepared hyaluronate/PEGylated chitosan lactate HA/PCL) NPs to simultaneously deliver IL6-specific siRNA and BV6 to 4T1 (breast cancer) and CT26 (colon cancer) cells. They investigated the anti-tumor properties of this combined therapy both in vitro and in vivo. Such therapy synergistically increased apoptosis and decreased cell migration, proliferation, colony formation, and angiogenesis in both 4T1 and CT26 cell lines and, by that, suppressed cancer progression in tumor-bearing mice that was associated with enhanced survival time.

Another strategy is to use BV6 along with inhibition of signal transducer and activator of transcription 3 (STAT3), which is an important factor in the survival of tumor cells, and NIK as a mediator of BV6 unpredicted side effects. This combination has the potential to induce effective apoptosis in tumor cells. Nikhoo et al. [223] used carboxymethyl dextran-conjugated trimethyl chitosan (CMD/TMC) NPs loaded with NIK/STAT3-specific siRNA and BV6 to synergistically induce apoptosis in the breast, colorectal, and melanoma cancer cell lines. Their results showed that in addition to enhanced pro-apoptotic effects, this combined therapy reduced proliferation, cell migration, colony formation, and angiogenesis, along with the expression of factors including IL-10 and HIF in the tumor cells. Masjedi et al. [224] generated the active-targeted hyaluronate (HA) recoated N, N, N-trimethyl chitosan (TMC) NPs to deliver IL-6- and STAT3-specific siRNAs to the CD44-expressing cancer cells. The results showed that the synthesized NPs had high transfection efficiency, low toxicity, and controlled siRNA release. The siRNA-loaded NPs significantly inhibited the IL-6/STAT3 expression, which was associated with blockade of proliferation, colony formation, migration, and angiogenesis in the cancer cells. 

A co-delivery of chemotherapeutic drugs and siRNA has gained increasing attention owing to the enhanced antitumor efficacy over the single administration. Studies in this field showed that simultaneous delivery of chemotherapeutic drugs and siRNA via a single targeted vector was more effective in treating some cancers than a sequential administration of two separate vectors with one drug in each. Yan et al. [225] developed a CS-based pH-responsive prodrug vector for the co-delivery of doxorubicin (DOX) and Bcl-2 siRNA. The accumulation of fabricated NPs in hepatoma cells was enhanced by glycyrrhetinic acid receptor-mediated endocytosis. This nanoplatform can efficiently integrate gene- and chemotherapies with a dramatically enhanced tumor inhibitory rate (88.0%) in vivo.

Gene therapy treatment strategies for Parkinson’s disease (PD) have recently come into prominence. Here, gene therapy has potential advantages to increase pre-cursor cells to synthesize dopamine and could repair or prevent degeneration of dopaminergic neurons. Gene therapy could correct a specific genetic defect by increasing, decreasing, or silencing the expression of target genes, or induce the endogenous production of a therapeutic protein. Xue et al. [226] proposed drug-loaded chitosan nanoparticles (pDNA-NGF-ActPP/CS NPs as novel candidates for the design of anti-PD drugs. They investigated the effects of chitosan polyethylene glycol-poly lactic acid (PP/CS) NPs conjugated with nerve growth factor (NGF), acteoside (Act), and plasmid DNA (pDNA) (pDNA-NGF-ActPP/CS NPs) for PD therapy using in vitro and in vivo models. Using PD cell models, they demonstrated that pDNA-NGF-ActPP/CS NPs had good neuroprotective effects. More significantly, experiments using a mouse PD model showed that pDNA-NGF-ActPP/CS NPs could ameliorate the behavioral disorders of sick mice. Immunohistochemical and Western blot (WB) analyses revealed that pDNA-NGF-ActPP/CS NPs could significantly reverse dopaminergic (DA) neuron loss in the substantia nigra and striatum of sick mice. This study opens up a novel avenue to develop anti-PD drugs.

## 4. Concluding Remarks

The recent review and research papers indicate CS NPs play a vital role in biomedical applications such as drug/vaccine/gene delivery, bioimaging, wound healing, tissue engineering, etc. They highlighted an outstanding position of CS as a polysaccharide able to form NPs favorable for various drug delivery purposes because of its many beneficial properties, such as mucoadhesion, controlled drug release, transfection, permeation enhancement, in situ gelation, efflux pump inhibitory properties, and stimuli-responsive properties. Many works demonstrated, via in vitro and in vivo experiments, CS NPs designed for controlled drug delivery may improve the stability of the drug and increase the efficacy of therapeutic agents. The other advantages of CS NPs DDS, presented in recent works, involved reduced therapeutical doses leading to reduced possible side effects, better bioavailability, and finally better patient compliance.

Nevertheless, the current research is still oriented towards an additional improvement of the chitosan properties. There are efforts to enhance its low solubility in physiological pH, stimuli-responsive properties, and specificity towards complex biological systems by chemical modifications of pure CS or by blending CS with other polymers or inorganic materials. In this way, new modified CS-based nanoparticles and nanocomposites possessing more or less enhanced properties were developed. Such innovative CS particulate systems provided, to a more/less extent, non-toxic, biocompatible, stable, target-specific, and biodegradable delivery devices. In addition, the systems with a proper label (e.g., metal-based nanocomposites) enabled target-specific diagnostics (due to easy dual introduction of an imaging agent together with a therapeutic agent) along with a target-specific therapy (due to a stimulus-responsive matrix). Recent newly developed native CS NPs, modified CS NPs, or CS nanocomposites were applied as potential drug carriers for many drugs and various routes of administrations. They were mainly studied for anticancer agents, proteins, vaccines, and genetic material. For example, in oral DD, new CS NPs enhanced the absorption of the drugs through the opening of tight junctions of the mucosal membrane. In ocular DD, in situ gelling properties and mucoadhesive character of CS enabled prolonging drug release. In nasal delivery, CS NPs increased the permeability of the drugs. In vaccine delivery, CS NPs enabled to formulate oral vaccines providing enhanced absorption of these hydrophilic biomolecules.

Despite apparent current progress, safety and targeting specificity are remaining among the main challenges in the future development of CS-based nanoparticulate DDSs. Therefore, systematic studies on biodistribution, in vitro and in vivo toxicity, and selectivity will further continue with newly developed CS derivatives and their NPs and nanocomposites for various administration routes.

## Figures and Tables

**Table 1:** Groups of modified chitosan and structures of particular derivatives.

Chitosan Derivative Groups/Derivatives	Formula
**Hydrophobic derivatives**	
Alkylated chitosan	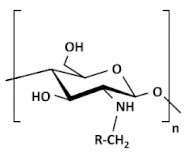
Acylated chitosan	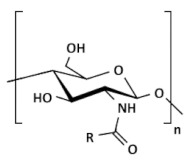 (A) N-acylated chitosan
	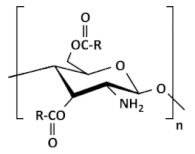 (B) O-acylated chitosan
N-phtaloylated chitosan	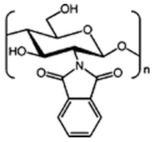
Benzoylated chitosan	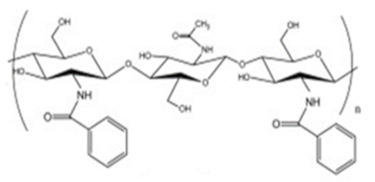
Methacrylated chitosan	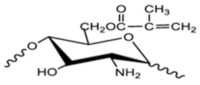
**Amphiphilic derivatives**	
Cholic and deoxycholic acid-modified chitosan	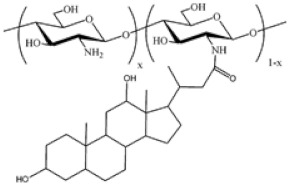 (A) Deoxycholic acid 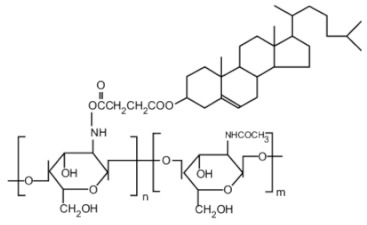 (B) Cholic acid
**Ionic derivatives**	
Quarternary ammonium chitosan derivatives	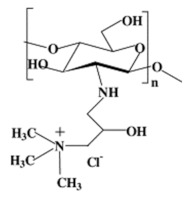
Sulfated chitosan derivatives	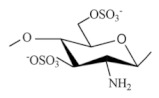
Succinylated chitosan	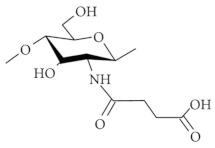
Sulfonated chitosan	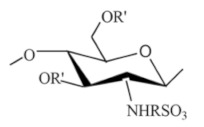
Phosphorylated chitosan	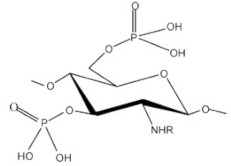
Carboxyalkylated chitosan (carboxymethylchitosan)	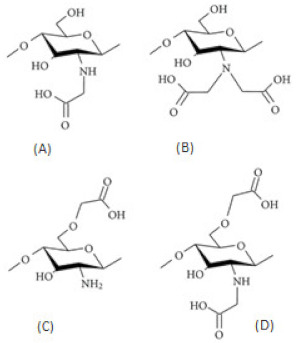 (A) N-CMC, (B) N,N-CMC, (C) O-CMC, and (D) N,O-CMC (showing the modification at the D-glucosamine unit)
**Chitosan copolymers**	
PEGylated chitosan	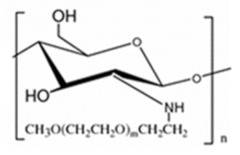
PEG-methacrylated chitosan	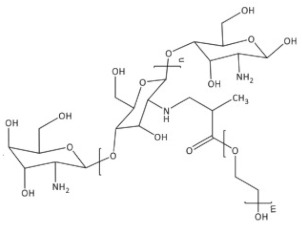
**Derivatives with specific substituents**	
Sugar bound chitosan derivatives	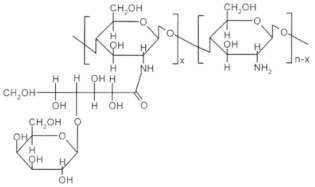 Galactosylated chitosan
	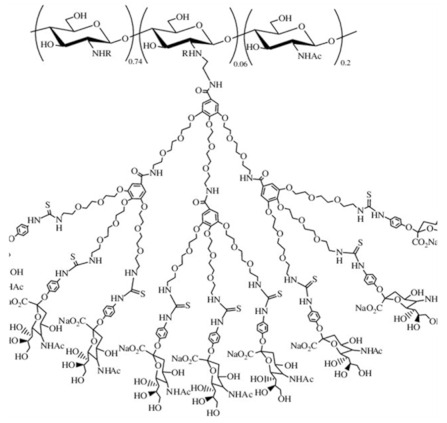 Sialo dendrimer hybrid chitosan
Chitosan derivatives with cyclic structure	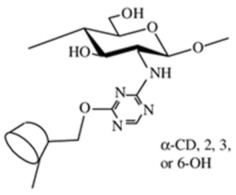 Crown ether-linked chitosan
	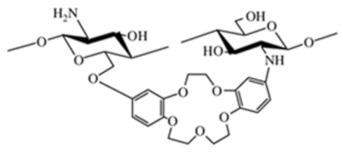 Cyclodextrin-linked chitosan
Chitosan derivatives with thiol groups	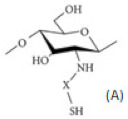 (A) Thiolated chitosan with –SH group
	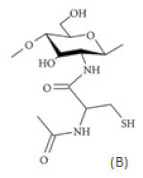 (B) Thiolated chitosan with cysteine: chitosan-N-acetyl-cysteine
Glycol chitosan	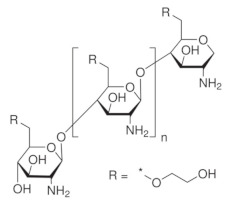
Thiosemicarbazone linked chitosan derivatives	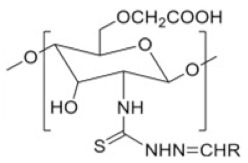
**Crosslinked chitosan derivatives**	
Chitosan-glutaraldehyde crosslinked polymer	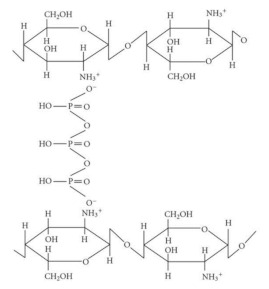
Chitosan-TPP crosslinked polymer	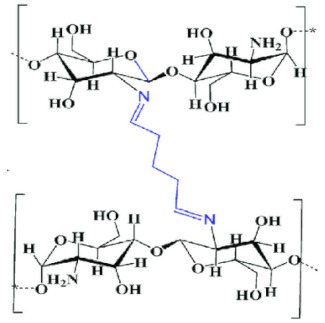
Chitosan-EDTA crosslinked polymer	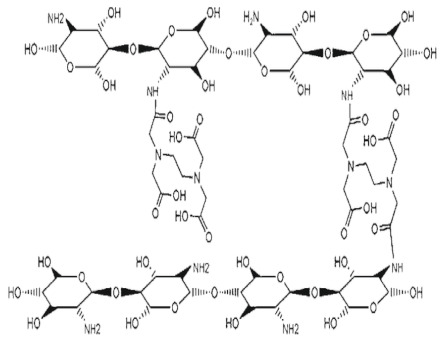

**Table 2:** Advantages and limitations of CS NPs [52].

Advantages of CS NPs	Disadvantages of CS NPs
Less toxicity	Less mechanical resistance
Enhanced biocompatibility	Difficult to control pore size
Mucoadhesive character	Possible contraction
Stability	Difficult electrospinning for pure chitosan
Site-specific drug targeting	Preparation by cross-linking can affect intrinsic properties of chitosan
The increased therapeutic index of the drug	Low solubility in neutral and alkaline pH
Frequent, expensive, and unpleasant dosing is prevented	Method of preparation depends on the drug to be delivered

**Table 3:** Current application examples of chitosan nanoparticles and nanocomposites in drug delivery.

Type of CS NP	Method of CS NP Preparation	Formulated Drug	In Vitro and In Vivo Tests for Biological Activity and Drug Release	Citation
**Oral drug delivery of antidiabetic drugs**				
TC NPs	Schiff-base linking with PETMP [pentaerythritol tetrakis (3-mercaptopropionate)]	Insulin	In vitro sustained drug release, in vitro cell viability, in vivo biodistribution, and pharmacokinetics	[160]
Snail mucin/CS NPs	Self-assembly	Insulin	In vitro drug release, in vivo hypoglycemic activity in diabetic rats, and toxicity	[161]
FD/TMC NPs	PEC method	Insulin	In vitro pH-dependent drug release, cytotoxicity, α-glucosidase inhibition assay	[162]
CS/Dz13Scr NPs	Complex coacervation	Insulin	In vitro drug release, insulin kinetics, cytotoxicity, mucus permeation, endocytic absorption study	[163]
CS NPs	Ionic gelation	Polydatin	In vitro drug release, cytotoxicity, in vivo antidiabetic activity in type 2 diabetic rats	[164]
**Oral delivery of anticancer drugs**				
FD/CS NPs	PEC method	Methotrexate (MTX) for lung cancer therapy	In vitro mucoadhesive study, in vitro antiproliferative assay and cellular uptake, apoptosis assay	[165]
M CS/P NPs	Ionic gelation	Curcumin (CUR)	Cytotoxicity, cellular uptake	[115]
chitosan-copaiba oil-poly (isobutyl cyanoacrylate) core-shell nanocapsules	Interfacial polymerization	For colon cancer therapy	In vitro mucoadhesion effect	[166]
Cys/PLA/CS NPs	Self-assembly	Paclitaxel (PTX)	In vitro drug release, cytotoxicity, and cellular uptake, in vivo pharmacokinetic study, biodistribution study, antitumor efficacy	[167]
TPGS/HPMC/CS NPs	Solvent evaporation method	Paclitaxel (PXT)	In vitro dissolution and swelling, Cytotoxicity, cellular uptake, transport study	[168]
**Oral delivery of antihypertensive drugs**				
CS NPs	Ionic gelation (TPP)	Carvedilol	In vitro drug release, in vivo pharmacokinetics on rats	[169]
**Oral delivery of antioxidants**				
CS/Zein NPs	Liquid-liquid dispersion	Resveratrol (RVT)	In vitro drug release, antioxidant activity, in vitro mucoadhesion study	[170]
**Oral delivery of anti-inflammatory drugs**				
CS/WP-NPs	Self-assembly	polysaccharides from *Ophiopogon japonicus* (OJPs) IBD treatment	In vitro drug release, Biocompatibility, cytotoxicity, antioxidant activity, gene expression, ex vivo mucoadhesion study	[171]
CS NPs	Spray-drying method	Dexketoprofen trometamol (DT)	In vitro prolonged drug release, release kinetics, in vivo anti-inflammatory activity, HET-CAM assay	[93]
AvrA NPs-ALG/CS MPs	Flow focusing microfluidic method	Salmonella effector enzyme (AvrA)	In vitro drug release, in vivo reduction of inflammation in murine dextran sulfate sodium (DSS) colitis model	[172]
**Oral vaccines**				
β-CD/CS NPs	Precipitation/coacervation method	Ovalbumin (OVA)	In vitro drug release, in vivo immune response in Balb/c mice	[173]
CS chloride NPs	Ionic gelation (TPP)	Ovalbumin (OVA)	In vitro cell toxicity, permeability study, transepithelial electrical resistance studies, in vivo studies	[174]
ALG/CS NPs anchored with lipopolysaccharide (LPS) as an adjuvant	Ionic gelation (TPP)	HBsAg antigen	In vitro drug release and mucoadhesion study, stability, cytotoxicity, in vivo immunization studies	[175]
ALG/CS coating LDHs	Co-precipitation-hydrothermal method	BSA	In vitro drug release, cellular uptake, stability in biological fluids	[176]
**Oral delivery of other drugs**				
Cs PLNs	Self-assembly	Enoxaparin	Mucoadhesive properties, stability, in vivo anticoagulant activity in rats	[177]
CS NPS	Double emulsification solvent evaporation method	Salmon calcitonin (sCT) and puerarin (PR)	In vitro drug release, stability, cellular uptake, in vivo pharmacokinetic study	[79]
SA/CS and NaCAS/CS NPs	Ionic gelation (oxidized dextran)	Astaxanthin (ASTX) (hepatic fibrosis treatment)	ABTS radical scavenging assay, cytotoxicity, anti-fibrogenic activity	[114]
Soy lecithin/CS hybrid NPs	Self-assembly	Raloxifene hydrochloride (RLX)	In vitro drug dissolution and release, MTT assay, intestinal drug uptake, in vivo pharmacokinetic studies, biodistribution, ex-vivo mucoadhesion studies	[178]
**Ocular drug delivery**				
GCS NPs	Self-assembly	Dexamethasone (DEX)	In vitro drug release, mucoadhesive, cytotoxicity, and anti-inflammatory efficacy, in vivo study: eye irritation test and distribution test	[179]
CMC/GSH/GlySar/LDHs	Coprecipitation–hydrothermal method	Dexamethasone disodium phosphate (DEXP) DD to the posterior segment of the eye	In vitro toxicity study on human conjunctival epithelial cells, cellular uptake, the in vivo precorneal retention study, the tissue distribution evaluation of rabbit’s eyes	[148]
CS/LIP	Thin-film hydration method	Triamcinolone acetonide (TA) Treatment of posterior eye segment diseases	In vitro drug release, in vivo drug release	[180]
TCM/LNPs	Emulsion solvent evaporation method	Baicalein (BAI)	In vitro sustained drug release, in vivo ocular irritation study, pre-corneal retention evaluation, pharmacokinetic study	[33]
CS NPs	Ionic gelation (TPP)	Levofloxacin (LFX) Therapy of ocular infections	The antimicrobial study, in vitro ocular tolerance, in vivo pharmacoscintigraphic study	[181]
CS/poly(ethylene glycol) methacrylate MNPs	Double crosslinking (ionic and covalent) in reverse emulsion	Bevacizumab Treatment of posterior segment of the eye	In vitro drug release kinetics, hemocompatibility, in vivo study of antiangiogenic effect (eye)	[48]
CS/PCL NPs	Single-step emulsification method	Dorzolamide (DRZ) Glaucoma treatment	In vitro drug release, in vivo corneal flux experiment, corneal hydration study, ex vivo bioadhesion study, ocular tolerance study, Hen egg test-chorioallantoic membrane (HET-CAM) test	[82]
CS/gelatin gel with CUR-NPs	-	Latanoprost (LP) and curcumin (CUR) Glaucoma treatment	In vitro drug release, in vitro biocompatibility, in vivo incompatibility in rabbits	[182]
**Nasal drug delivery (topical)**				
CS NPs	Ionic gelation	Cromolyn Therapy of allergic rhinitis	In vitro drug release, permeation, and penetration, mucoadhesion assay	[183]
DCHBC NPs	Dialysis method	Cetirizine (CTZ) Therapy of allergic rhinitis	In vitro stimuli-responsive drug release, cytotoxicity, hemolysis test, protein adsorption	[184]
CS or CS maleimide NPs	Ionic gelation (TPP)	Japanese encephalitis-chimeric virus vaccineNasal vaccine	Mucoadhesive properties, antigen uptake study, in vivo study of immunization of mice	[185]
**Nose to brain delivery**				
CS/HSA NPs	Desolvation method	Tacrine and R-flurbiprofen	mucoadhesion properties, in vitro drug release, permeation, uptake, ex vivo diffusion experiments on rabbit nasal mucosa	[186]
PLGA NPs and PLGA/CS NPs	Nanoprecipitation	Ropinirole hydrochloride Antiparkinson therapy	In vitro drug release, mucoadhesion, hemolysis assay, stability study, studies on peripheral blood mononuclear cells and RAW 264.7 macrophage cell line—cytotoxicity, cellular uptake ex vivo permeability studies	[187]
N,O-CMC NPs	Emulsion solvent evaporation method	Dopamine (DOPA) orTyrosine (Tyr)	In vitro drug release, mucoadhesive properties, cytotoxicity, cellular uptake	[188]
CS NPs	-	Therapy of Huntington disease	Gene silencing studies	[189]
CS NPs	Ionic gelation (TPP)	Zolmitriptan (ZOL) Therapy of migraine	In vivo stability, in vivo pharmacokinetic study on Wistar rats	[190]
CS NPs	Ionic gelation (TPP)	Rotigotine (R) Treatment of Parkinson’s disease	In vitro cellular uptake, cytotoxicity assay, neuroprotective activity, antioxidant activity, in vivo pharmacodynamic and pharmacokinetic study	[191]
**Pulmonary (inhalation) drug delivery**				
CS NPs	Emulsion method	Nicotine hydrogen tartrate (NHT) Treatment of nicotine addiction	In vitro evaluation of nose-only inhalation device, assessment of bioactivity of NHT-CS NPs via locomotor test by injection, histopathological analysis of lung tissues	[192]
CS/PLGA NPs followed by coating with chitosan	Solvent evaporation (double-emulsion) method	Catechin hydrate (CTH)	In vitro drug release, ex-vivo permeation study on the nasal mucosa, cytotoxicity, in vivo comparative pulmokinetic study	[193]
CS/SLNs	Hot ultrasonication	Rifampicin (RIF) Tuberculosis treatment	In vitro drug release, mucoadhesive properties, in vitro cell viability and permeability studies, stability studies	[194]
Mn-TMC NPs	Ionic gelation (TPP)	Etofylline (ETO) Asthma treatment	Sustained drug release, biodegradation studies, stability, safety, and aerodynamic behavior	[195]
HA/CS NPs	Self-assembly	Ferulic acid (FA) Asthma treatment	In vivo inhalation toxicity assessment	[196]
**Buccal delivery**				
CS NPs	Ionic gelation (TPP)	Oxiplatin Anticancer therapy	ex vivo its penetration in porcine mucosa under both passive and iontophoretic topical treatments	[102]
Cat/CS/HA NPs	Ionic gelation	Doxorubicin (DOX) Oral cancer treatment	Ex vivo mucoadhesive study, in vitro drug release, cytotoxicity, cellular uptake, cancer cells death	[103]
TTEC NPs	PEC method	Insulin	In vitro drug release, ex vivo permeation study on rabbit mucosa, MTT assay	[197]
CS/PEO NFs	Electrospinning	Sublingval delivery	Ex vivo adhesion on porcine mucosa, swelling, compatibility	[198]
**Periodontal delivery**				
CS NPs	Ionic gelation	Minocycline, tetracycline Periodontal disease	Human gingival fibroblasts behavior, Cell viability and culture metabolic activity, cellular uptake, inflammatory gene expression	[199]
Core-sheath NFs: shell layer: CS core: PVA containing drug	Coaxial electrospinning and ionic gelation (genipin)	Tetracycline hydrochloride (TH) Periodontitis treatment	In vitro sustained drug release, in vitro antimicrobial activity, cytotoxicity	[200]
CS/IO NPs	-	Chlorhexidine (CHX) Antimicrobial and antibiofilm effect against oral disease	Determination of MIC, cytotoxicity by MTT assay	[126]
**Dermal drug delivery**				
CS NPs	Ionic gelation (TPP)	Nicotinamide	Clinical test, skin bioadhesion, deposition of drug in different skin layers	[201]
Poly-(ε-caprolactone)-lipid core NCs nad CS/poly-(ε-caprolactone)-lipid-core NCs	Interfacial deposition technique	Dutasteride Hair follicle targeting after massage procedure	In vitro drug release, stability, in vitro skin permeation	[202]
**Transdermal drug delivery**				
CS Hydroxypropyltrimonium chloride/PLGA NPs	Antisolvent diffusion method	Hen egg-white lysozyme (HEL) allergen immunotherapy to hair follicles using iontophoresis	*In vitro* cellular uptake, ex vivo skin accumulation study, in vivo transcutaneous immunization experiment	[203]
CS NFs	Electrospinning	Colchicine Anti-skin cancer therapy	Ex vivo skin permeation, deposition analysis, release kinetic and anti-melanoma efficiency against A-375 cell line	[204]
CS NPs	Nanospray-drying technique	5-fluorouracil (FU)	Synergistic microwave delivery of anti-cancer	[205]
**Wound healing**				
CS/PEO NFs	Electrospinning	Teicoplanin Local antibiotic wound healing	In vitro drug release, antibacterial test, cytotoxicity, in vivo study on rat full-thickness wound model	[67]
PCL/CS NFs	Electrospinning	Curcumin (CUR) Wound dressing	antibacterial, antioxidant properties, cell viability, and in vivo wound healing efficiency and histological assay	[206]
hydrogel membranes based on HA/PU/PVA loaded with cefepime-CS NPs	Ionic gelation (TPP)	Cefepime	In vitro drug release, bacterial inhibition	[207]
CS NPs loaded hydrogel	Ionic gelation (TPP)	Pterocarpus marsupium heartwood extract (PM) Therapy of diabetic wounds	In vitro drug release efficiency, in-vitro anti-microbial activity, in vivo wound healing action in streptozotocin administered diabetic rat models	[208]
**Vaginal drug delivery**				
CS NPs	Ionic gelation (TPP)	Miconazole nitrate Therapy of vulvovaginal candidiasis	In vivo evaluation on vulvovaginal murine model	[209]
CS NPs encapsulated in hydrophilic freeze-dried cylinders	Ionic gelation (TPP)	Insulin Peptide-based vaccines or delivery of microbicides	In vitro drug release, ex vivo insulin penetration across porcine vaginal mucosa	[210]
CS and spicules NPs	Ionic gelation (TPP)	Calophycin A (Cal A)—seaweed-derived metabolite Therapy of vaginal candidiasis	In vitro anti-candidal activity, in vivo on mice	[211]
**Vaccine delivery**				
CS and ALG coated CS NPs	Precipitation/coacervation method	Hepatitis A vaccine (HAV)	Assay of HAV-specific antibodies and their isotypes, lymphoproliferation assay, the effect of HAV formulation on the splenocytes proliferation in vaccinated mice	[212]
CS NPs	Ionic gelation (TPP)	Aah II toxin isolated from Androctonus australis hector (scorpion) venom	In vitro toxin-release study, in vivo immunization trial	[213]
**Gene delivery**				
MPC derived from carbonized CTS echitosan capsulated ZIF-8	Carbonization	Luciferase-expressing plasmid (pGL3), and splice correction oligonucleotides (SCO)	Cell biocompatibility, transfection efficiency, mechanism of uptake	[214]
LMW mannosylated CS NPs	Ionic gelation	CpG oligodeoxynucleotides	Cytotoxicity, cellular uptake, immunostimulatory effect-cytokine release in RAW264.7 cells, efficient vector for intracellular CpG ODN delivery	[215]
TMC Cys, MABCMC, and CysMABC NPs	Ionic gelation	Plasmid DNA pEGFP-N1	In vitro DNA transfection efficiency, cytotoxicity	[216]
CS, PEI, and CMD NPs	Self-assembly	Anti-HIV siRNA HIV therapy	In vitro cytotoxicity assay and siRNA delivery in two mammalian cell lines, macrophage RAW264.7, and HEK293	[217]
TMC/DS or ALG NPs	PEC method	hSET1 antisense—silencing oligonucleotide Cancer therapy	In vitro cell viability, cellular uptake, in vivo study on mice	[218]
CMC NPs labelled with FITC NPs (FITCCS/CMC)	Self-assembly	Anti-β-catenin siRNA Ultrasound-triggered targeted therapy of colon cancer	In vitro drug release, cytotoxic assay, cellular uptake, therapeutic evaluation	[219]
Guanidinylated O-CMC NPs (GOCMCS)	Self-assembly	SiRNA delivery	In vitro cell transfection studies with A549 cells, cellular uptake	[220]
SPION NPs encapsulated with TAT peptide/TC and TMC	Electrostatic interaction	siRNA Targeted anti-cancer therapy	Cytotoxicity, cellular internalization, in vivo pharmacokinetic and biodistribution, colony formation assay, wound healing assay, Chick chorioallantoic membrane (CAM) assay	[221]
HA/PCL NPs	Ionic gelation (TPP)	IL6-specific siRNA and BV6 treatment of breast and colon cancer	In vitro drug release, cellular uptake, MTT assay, apoptosis assay, Chick chorioallantoic membrane assay, wound healing assay, a clonogenic assay of tumor cells in vitro, transwell migration assay, in vivo antitumor efficacy on mice	[222]
CMD/TMC NPs	Nanoprecipitation	Codelivery of NIK/STAT3-specific siRNA and BV6 Cancer therapy	Stability of NPs, in vitro drug release, cellular uptake, transfection of cells, MTT assay, Chick chorioallantoic membrane (CAM) assay, wound healing assay, colony formation assay	[223]
HA/TMC NPs	PEC method	IL-6- and STAT3-specific siRNAs Cancer therapy	In vitro drug release, stability in serum, MTT cytotoxicity assay, cellular uptake, transfection efficiency, Colony formation assay Wound healing assay	[224]
CS NPs	-	Doxorubicin (DOX) and Bcl-2 siRNA co-delivery of therapeutics and si-RNA Cancer therapy	In vitro drug release, in vivo tumor suppression test	[225]
Polyethyleneglycol-poly lactic acid CS (PP CS NPs)	-	Nerve growth factor (NGF), acteoside (ACT), and plasmid DNA (pDNA) Treatment of Parkinson’s disease	Plasmid DNA (pDNA), nerve growth factor (NGF), acteoside (Act)	[226]

## Data Availability

Not relevant.

## References

[1] Kumar A., Vimal A., Kumar A. (2016). Why chitosan? From properties to perspective of mucosal drug delivery. Int. J. Biol. Macromol..

[2] Shanmuganathan R., Edison N.T.J.I., Lewis Oscar F., Kumar P., Shanmugan S., Pugazhendhi A. (2019). Chitosan nanopolymers: An overview of drug delivery against cancer. Int. J. Biol. Macromol..

[3] Taher F.A., Ibrahim S.A., Abd El-Aziz A., El-Nour M.F.A., El-Sheikh M.A., El-Husseiny N., Mohamed M.M. (2019). Anti-Proliferative effect of chitosan nanoparticles (extracted from crayfish *Procambarus clarkii*, Crustacea: Cambaridae) against MDA-MB-231 and SK-BR-3 human breast cancer cell lines. Int. J. Biol. Macromol..

[4] El Rabey H.A., Almutairi F.M., Alalawy A.I., Al-Duais M.A., Sakran M.I., Zidan N.S., Tayel A.A. (2019). Augmented control of drug-resistant *Candida* spp. via fluconazole loading into fungal chitosan nanoparticles. Int. J. Biol. Macromol..

[5] Lim C., Lee D.W., Israelachvili J.N., Jho Y.S., Hwang D.S. (2015). Contact time- and pH-dependent adhesion and cohesion of low molecular weight chitosan coated surfaces. Carbohydr. Polym..

[6] Lim C., Hwang D.S., Lee D.W. (2021). Intermolecular interactions of chitosan: Degree of acetylation and molecular weight. Carbohydr. Polym..

[7] Elgadir A.A., Muddin M.S., Ferdosh S., Adam A., Chowdhury A.J.K., Sarker M.Z.I. (2015). Impact of chitosan composites and chitosan nanoparticle composites on various drug delivery systems: A review. J. Food Drug Anal..

[8] Bernkop-Schnurch A., Dunnhaupt S. (2012). Chitosan-based drug delivery systems. Eur. J. Pharm. Biopharm..

[9] Saikia C., Gogoi P., Maji T.K. (2015). Chitosan: A promising biopolymer in drug delivery applications. J. Mol. Genet. Med.

[10] Huang G., Liu Y., Chen L. (2017). Chitosan and its derivatives as vehicles for drug delivery. Drug Deliv..

[11] Li J., Cai C., Li J., Li J., Li J., Sun T., Wang L., Wu H., Yu G. (2018). Chitosan-Based nanomaterials for drug delivery. Molecules.

[12] Safdar R., Omar A.A., Arunagiri A., Regupathi I., Thanabalan M. (2019). Potential of chitosan and its derivatives for controlled drug release application—A review. J. Drug Deliv. Sci. Technol..

[13] Bakshi P.S., Selvakumar D., Kadirvelu K., Kumar N.S. (2020). Chitosan as an environment friendly biomaterial—A review on recent modififications and applications. Int. J. Biol. Macromol..

[14] Khan F., Pham D.T.N., Oloketuyi S.F., Manivasagan P., Oh J., Kim Y.M. (2020). Chitosan and their derivatives: Antibiofilm drugs against pathogenic bacteria. Colloids Surf. B Biointerfaces.

[15] Ma Y., Garrido-Maestu A., Jeong K.C. (2017). Application, mode of action, and in vivo activity of chitosan and its micro- and nanoparticles as microbial agents: A review. Carbohydr. Polym..

[16] Qin Y., Li P., Gou Z. (2020). Cationic chitosan derivatives as potential antifungals: A review of structural optimization and applications. Carbohydr. Polym..

[17] Hamedi H., Moradi S., Hudson M., Tonelli A.E. (2018). Chitosan based hydrogels and their applications for drug delivery in wound dressings: A review. Carbohydr. Polym..

[18] Ali A., Ahmed S. (2018). A review on chitosan and its nanocomposites in drug delivery. Int. J. Biol. Macromol..

[19] Negm N.A., Hefni H.H.H., Abd-Elaal A.A.A., Badr E.A., Abou Kana M.T.H. (2020). Advancement on modifification of chitosan biopolymer and its potential applications. Int. J. Biol. Macromol..

[20] Tian Y., Sun Y., Wang X., Kasparis G., Mao S., Grumezescu M.A. (2016). Chapter 15—Chitosan and its derivatives-based nano-formulations in drug delivery. Nanobiomaterials in Drug Delivery.

[21] Mateescu M.A., Ispas-Szabo P., Assaad E. (2015). Chitosan and its derivatives as self-assembled systems for drug delivery In Controlled Drug Delivery: The Role of Self-Assembling Multi-Task Excipients.

[22] Fonseca-Santos B., Chorilli M. (2017). An overview of carboxymethylated derivatives of chitosan: Their use as biomaterials and drug delivery systems. Mater. Sci. Eng. C.

[23] Dimassi S., Tabary N., Chai F., Blanchemain N., Martel B. (2018). Sulfonated and sulfated chitosan derivatives for biomedical applications: A review. Carbohydr. Polym..

[24] Tekade M., Maheshwari N., Youngren-Ortiz S.R., Pandey V., Chourasiya Y., Soni V., Deb P.K., Sharma M.C. (2019). Chapter 13—Thiolated-Chitosan: A novel mucoadhesive polymer for better-targeted drug delivery. Advances in Pharmaceutical Product Development and Research, Biomaterials and Bionanotechnology.

[25] Dang Y., Guan J. (2020). Nanoparticle-based drug delivery systems for cancer therapy. Smart Mater. Med..

[26] Wang W., Xue C., Mao X. (2020). Chitosan: Structural modification, biological activity and application. Int. J. Biol. Macromol..

[27] Chu L., Zhang Y., Feng Z., Yang J., Tian Q., Yao X., Zhao X., Tan H., Chen Y. (2019). Synthesis and application of a series of amphipathic chitosan derivatives and the corresponding magnetic nanoparticle-embedded polymeric micelles. Carbohydr. Polym..

[28] Burr S.J., Williams P.A., Ratcliffe I. (2018). Synthesis of cationic alkylated chitosans and an investigation of their rheological properties and interaction with anionic surfactant. Carbohydr. Polym..

[29] Nanda B., Manjappa A.S., Chuttani K., Balasinor N.H., Mishra A.K., Ramachandra Murthy R.S. (2019). Acylated chitosan anchored paclitaxel loaded liposomes: Pharmacokinetic and biodistribution study in Ehrlich ascites tumor bearing mice. Int. J. Biol. Macromol..

[30] Permadi R., Suk V.R.E., Misran M. (2020). Synthesis and Characterization of acylated low molecular weight chitosan and acylated low molecular weight phthaloyl chitosan. Sains Malaysiana.

[31] Mohamed N.A., Abd El-Ghany N.A., Abdel-Aziz M.M. (2021). Synthesis, characterization, anti-inflammatory and anti-*Helicobacter pylori* activities of novel benzophenone tetracarboxylimide benzoyl thiourea cross-linked chitosan hydrogels. Int. J. Biol. Macromol..

[32] Hanafy A.F., Abdalla A.M., Guda T.K., Gabr K.E., Royall P.G., Alqurshi A. (2019). Ocular anti-inflammatory activity of prednisolone acetate loaded chitosan-deoxycholate self-assembled nanoparticles. Int. J. Nanomed..

[33] Li J., Jin X., Yang Y., Zhang L., Liu R., Li Z. (2020). Trimethyl chitosan nanoparticles for ocular baicalein delivery: Preparation, optimization, in vitro evaluation, in vivo pharmacokinetic study and molecular dynamics simulation. Int. J. Biol. Macromol..

[34] Ravindran R., Mitra K., Arumugam S.K., Doble M. (2021). Preparation of Curdlan sulphate—Chitosan nanoparticles as a drug carrier to target Mycobacterium smegmatis infected macrophages. Carbohydr. Polym..

[35] Ravi Kumar M.N.V., Muzzarelli R.A.A., Muzzarelli C., Sashiwa H., Domb A.J. (2004). Chitosan chemistry and pharmaceutical perspectives. Chem. Rev..

[36] Palacio J., Monsalve Y., Ramírez-Rodríguez F., López B. (2020). Study of encapsulation of polyphenols on succinyl-chitosan nanoparticles. J. Drug Deliv. Sci. Technol..

[37] Han G., Liu S., Pan Z., Lin Y., Ding S., Li L., Luo B., Jiao Y., Zhou C. (2020). Sulfonated chitosan and phosphorylated chitosan coated polylactide T membrane by polydopamine-assisting for the growth and osteogenic differentiation of MC3T3-E1s. Carbohydr. Polym..

[38] Zheng Z., Zhang W., Sun W., Li X., Duan J., Cui J., Feng Z., Mansour H. (2013). Inflfluence of the carboxymethyl chitosan anti-adhesion solution on the TGF-β1 in a postoperative peritoneal adhesion rat. J. Mater. Sci. Mater. Med..

[39] Liu X., He Z., Chen Y., Zhou C., Wang C., Liu Y., Feng C., Yang Z., Li P. (2020). Dual drug delivery system of photothermal-sensitive carboxymethyl chitosan nanosphere for photothermal-chemotherapy. Int. J. Biol. Macromol..

[40] Wang C., Zhang Z., Chen B., Gu L., Li Y., Yu S. (2018). Design and evaluation of galactosylated chitosan/graphene oxide nanoparticles as a drug delivery system. J. Colloid Interface Sci..

[41] Sharma A.K., Gupta L., Sahu H., Qayum A., Singh S.K., Nakhate K.T., Ajazuddin, Gupta U. (2018). Chitosan engineered PAMAM dendrimers as nanoconstructs for the enhanced anti-cancer potential and improved in vivo brain pharmacokinetics of temozolomide. Pharm. Res..

[42] Evangelista T.F.S., Andrade G.R.S., Nascimento K.N.S., dos Santos S.B., Costa Santos M.F., D’Oca C.R.M., Estevam C.S., Gimenez I.F., Almeida L.E. (2020). Supramolecular polyelectrolyte complexes based on cyclodextrin-grafted chitosan and carrageenan for controlled drug release. Carbohydr. Polym..

[43] Yi Y., Wang Y., Liu H. (2003). Preparation of new crosslinked chitosan with crown ether and their adsorption for silver ion for antibacterial activities. Carbohydr. Polym..

[44] Guaresti O., Maiz–Fernández S., Palomares T., Alonso-Varona A., Eceiza A., Pérez-Álvarez L., Gabilondo N. (2020). Dual charged folate labelled chitosan nanogels with enhanced mucoadhesion capacity for targeted drug delivery. Eur. Polym. J..

[45] Adhikari H.S., Yadav P.N. (2018). Anticancer activity of chitosan, chitosan derivatives, and their mechanism of action. Int. J. Biomater..

[46] Qin Y., Xing R., Liu S., Li K., Meng X., Li R., Cui J., Li B., Li P. (2012). Novel thiosemicarbazone chitosan derivatives: Preparation, characterization, and antifungal activity. Carbohydr. Polym..

[47] Hsu C.V., Hsieh M.H., Xiao M.C., Chou Y.H., Wang T.H., Chiang W.H. (2020). pH-Responsive polymeric micelles self-assembled from benzoic-imine-containing alkyl-modifified PEGylated chitosan for delivery of amphiphilic drugs. Int. J. Biol. Macromol..

[48] Savin C.L., Popa M., Delaite C., Costuleanu M., Costin S., Peptu C.A. (2019). Chitosan grafted-poly(ethylene glycol) methacrylate nanoparticles as carrier for controlled release of bevacizumab. Mat. Sci. Eng. C.

[49] Yu S., Zhang X., Tan G., Tian L., Liu D., Liu Y., Yang X., Pan W. (2017). A novel pH-induced thermosensitive hydrogel composed of carboxymethyl chitosan and poloxamer cross-linked by glutaraldehyde for ophthalmic drug delivery. Carbohydr. Polym..

[50] Zhuang S., Zhang Q., Wang J. (2021). Adsorption of Co2+ and Sr2+ from aqueous solution by chitosan grafted with EDTA. J. Mol. Liq..

[51] Sreekumar S., Goycoolea F.M., Moerschbacher B.M., Rivera-Rodriguez G.R. (2018). Parameters influencing the size of chitosan-TPP nano- and microparticles. Sci. Rep..

[52] Garg U., Chauhan S., Nagaich U., Jain N. (2019). Current advances in chitosan nanoparticles based drug delivery and targeting. Adv. Pharm. Bull..

[53] Agnihotri S.A., Mallikarjuna N.N., Aminabhavi T.M. (2004). Recent advances on chitosan-based micro- and nanoparticles in drug delivery. J. Control. Release.

[54] Shoueir K.R., El-Desouky N., Rashad M.M., Ahmed M.K., Janowska I., El-Kemary M. (2021). Chitosan based-nanoparticles and nanocapsules: Overview, physicochemical features, applications of a nanofibrous scaffold, and bioprinting. Int. J. Biol. Macromol..

[55] Zhang A., Meng K., Liu Y., Pan Y., Qu W., Chen D., Xie S. (2020). Absorption, distribution, metabolism, and excretion of nanocarriers in vivo and their influences. Adv. Colloid Interface Sci..

[56] Cheng X., Tian X., Wu A., Li J., Tian J., Chong Y., Chai Z., Zhau Y., Chen C., Ge C. (2015). Protein corona influences cellular uptake of gold nanoparticles by phagocytic and nonphagocytic cells in a size-dependent manner. ACS Appl. Mater. Interfaces.

[57] Malhaire H., Gimel J., Roger E., Benoît J., Lagarce F. (2016). How to design the surface of peptide-loaded nanoparticles for efficient oral bioavailability?. Adv. Drug Deliv. Rev..

[58] Lee J.H., Sahu A., Jang C., Tae G. (2015). The effect of ligand density on in vivo tumor targeting of nanographene oxide. J. Control. Release.

[59] Zhao Z., Ukidve A., Krishnan V., Mitragotri S. (2019). Effect of physicochemical and surface properties on in vivo fate of drug nanocarriers. Adv. Drug Deliv. Rev..

[60] Sharma G., Valenta D.T., Altman Y., Harvey S., Xie H., Mitragotri S., Smith J.W. (2010). Polymer particle shape independently influences binding and internalization by macrophages. J. Control. Release.

[61] Banerjee A., Qi J., Gogoi R., Wong J., Mitragotri S. (2016). Role of nanoparticle size, shape and surface chemistry in oral drug delivery. J. Control. Release.

[62] Elci S.G., Jiang Y., Yan B., Kim S.T., Saha K., Moyano D.F., Tonga G.Y., Jackson L.C., Rotello V.M., Vachet R.W. (2016). Surface Charge Controls the Suborgan Biodistributions of Gold Nanoparticles. Nano.

[63] Xiao K., Li Y., Luo J., Lee J.S., Xiao W., Gonik A.M., Agarwal R.G., Lam K.S. (2011). The effect of surface charge on in vivo biodistribution of PEG-oligocholic acid based micellar nanoparticles. Biomaterials.

[64] Wu L., Liu M., Shan W., Zhu X., Li L., Zhang Z., Huymg Y. (2017). Bioinspired butyrate-functionalized nanovehicles for targeted oral delivery of biomacromolecular drugs. J. Control. Release.

[65] Castro A., Berois N., Malanga A., Ortega C., Oppezzo P., Pristch O., Mombrú A.W., Osinaga E., Pardo E. (2021). Docetaxel in chitosan-based nanocapsules conjugated with an anti-Tn antigen mouse/human chimeric antibody as a promising targeting strategy of lung tumors. Int. J. Biol. Macromol..

[66] Abid S., Hussain T., Nazir A., Zahir A., Khenoussi N. (2019). A novel double-layered polymeric nanofiber-based dressing with controlled drug delivery for pain management in burn wounds. Polym. Bull..

[67] Amiri N., Ajami S., Shahroodi A., Jannatabadi N., Darban S.A., Bazzaz B.S.F., Pishavar E., Kalalinia F., Movaffagh J. (2020). Teicoplanin-loaded chitosan-PEO nanofibers for local antibiotic delivery and wound healing. Int. J. Biol. Macromol..

[68] Quinones J.P., Peniche H., Peniche C. (2018). Chitosan based self-assembled nanoparticles in drug delivery. Polymers.

[69] Roy H., Nayak B.S., Nandi S. (2020). Chitosan anchored nanoparticles in current drug development utilizing computer-aided pharmacokinetic modeling: Case studies for target specific cancer treatment and future prospective. Curr. Pharm. Des..

[70] Hassani A., Hussain S.A., Abdullah N., Kmaruddin S. (2018). Review on micro-encapsulation with Chitosan for pharmaceutical applications. MOJ Curr. Res. Rev..

[71] Sang Z., Qian J., Han J., Deng X., Shen J., Li G., Xie Y. (2020). Comparison of three water-soluble polyphosphate tripolyphosphate, phytic acid, and sodium hexametaphosphate as crosslinking agents in chitosan nanoparticle formulation. Carbohydr. Polym..

[72] Pan C., Qian J., Zhao C., Yang H., Zhao X., Guo H. (2020). Study on the relationship between crosslinking degree and properties of TPP crosslinked chitosan nanoparticles. Carbohydr. Polym..

[73] Echeverri-Cuartas C.E., Gartner C., Lapitsky Y. (2020). PEGylation and folate conjugation effects on the stability of chitosan-tripolyphosphate nanoparticles. Int. J. Biol. Macromol..

[74] Abdelgawad A.M., Hudson S.M. (2019). Chitosan nanoparticles: Polyphosphates cross-linking and protein delivery properties. Int. J. Biol. Macromol..

[75] Cai Y., Lapitsky Y. (2020). Biomolecular uptake effects on chitosan/tripolyphosphate micro- and nanoparticle stability. Colloids Surf. B-Biointerfaces.

[76] Cai Y., Lapitsky Y. (2019). Pitfalls in analyzing release from chitosan/tripolyphosphate micro- and nanoparticles. Eur. J. Pharm. Biopharm..

[77] Wu D., Zhu L., Li Y., Zhang X., Xu S., Yang G., Delair T. (2020). Chitosan-Based colloidal polyelectrolyte complexes for drug delivery: A review. Carbohydr. Polym..

[78] Boudoukhani M., Yahoum M.M., Lefnaoui S., Moulai-Mostefa N., Banhobre M. (2019). Synthesis, characterization and evaluation of deacetylated xanthan derivatives as new excipients in the formulation of chitosan-based polyelectrolytes for the sustained release of tramadol. Saudi Pharm. J..

[79] Liu L., Yang H., Lou Y., Wu J.-Y., Miao J., Lu X.-Y., Gao J.-Q. (2019). Enhancement of oral bioavailability of salmon calcitonin through chitosan-modified, dual drug-loaded nanoparticles. Int. J. Pharm..

[80] Luesakul U., Puthong S., Sansanaphongpricha K., Muangsin N. (2020). Quaternizedchitosan-Coated nanoemulsions: A novel platform for improving the stability, anti-inflammatory, anti-cancer and transdermal properties of Plai extract. Carbohydr. Polym..

[81] Chaudhary S., Kumar S., Kumar V., Sharma R. (2020). Chitosan nanoemulsions as advanced edible coatings for fruits and vegetables: Composition, fabrication and developments in last decade. Int. J. Biol. Macromol..

[82] Shahab M.S., Rizwanullah M., Alshehri S., Imam S.S. (2020). Optimization to development of chitosan decorated polycaprolactone nanoparticles for improved ocular delivery of dorzolamide: In vitro, ex vivo and toxicity assessments. Int. J. Biol. Macromol..

[83] Krishna Sailaja A., Amareshwar P. (2011). Preparation of bovine serum albumin loaded chitosan nanoparticles using reverse micelle method. Res. J. Pharm. Biol. Chem. Sci..

[84] Zhang K., Xu Y., Lu L., Shi C., Huang Y., Mao Z., Duan C., Ren X., Guo Y., Huang C. (2021). Hydrodynamic cavitation: A feasible approach to intensify the emulsion cross-linking process for chitosan nanoparticle synthesis. Ultrason. Sonochem..

[85] Zhang H., Li X., Kang H. (2019). Chitosan coatings incorporated with free or nano-encapsulated *Paulownia Tomentosa* essential oil to improve shelf-life of ready-to-cook pork chops. LWT Food Sci. Technol..

[86] Riegger B.R., Bäurer B., Mirzayeva A., Tovar G.E.M., Bach M. (2018). A systematic approach of chitosan nanoparticle preparation via emulsion crosslinking as potential adsorbent in wastewater treatment. Carbohydr. Polym..

[87] Kumar S., Dilbaghi N., Saharan R., Bhanjana G. (2012). Nanotechnology as emerging tool for enhancing solubility of poorly water-soluble drugs. BioNanoScience.

[88] Seyam S., Nordin N.A., Alfatama M. (2020). Recent Progress of Chitosan and Chitosan Derivatives-Based Nanoparticles: Pharmaceutical Perspectives of Oral Insulin Delivery. Pharmaceuticals.

[89] Essa D., Choonara Y.E., Kondiah P.P.D., Pillay V. (2020). Comparative nanofabrication of PLGA-Chitosan-PEG systems employing microfluidics and emulsification solvent evaporation techniques. Polymers.

[90] Orellano M.S., Longo G.S., Porporatto C., Correa N.M., Falcone R.D. (2020). Role of micellar interface in the synthesis of chitosan nanoparticles formulated by reverse micellar method. Colloid Surf. A Physicochem. Eng. Asp..

[91] Haidar M.K., Demirbolat G.M., Timur S.S., Gürsoy R.N., Nemutlu E., Ulubayram K., Öner L., Eroğlu H. (2020). Atorvastatin-Loaded nanosprayed chitosan nanoparticles for peripheral nerve injury. Bioinspired Biomimetic Nanobiomater..

[92] Lucas J., Ralaivao M., Estevinho B.N., Rocha F. (2020). A new approach for the microencapsulation of curcumin by a spray drying method, in order to value food products. Powder Technol..

[93] Ozturk A.A., Kıyan H.T. (2020). Treatment of oxidative stress-induced pain and inflammation with dexketoprofen trometamol loaded different molecular weight chitosan nanoparticles: Formulation, characterization and anti-inflammatory activity by using in vivo HET-CAM assay. Microvasc. Res..

[94] Peng H.H., Hong D.X., Guan Y.X., Yao S.J. (2019). Preparation of pH-responsive DOX-loaded chitosan nanoparticles using supercritical assisted atomization with an enhanced mixer. Int. J. Pharm..

[95] Peng H.H., Wang Z.D., Guan Y.X., Yao S.J. (2021). Supercritical CO_2_ assisted preparation of chitosan-based nano-in-microparticles with potential for efficient pulmonary drug delivery. J. CO2 Util..

[96] Jalvo B., Faraldos M., Bahamonde A., Rosal R. (2018). Antibacterial surfaces prepared by electrospray coating of photocatalytic nanoparticles. Chem. Eng. J..

[97] Pawar A., Thakkar S., Misra M. (2018). A Bird’s Eye view of nanoparticles prepared by electrospraying: Advancements in drug delivery field. J. Control. Release..

[98] Kurakula M., Naveen N.R. (2021). Electrospraying: A facile technology unfolding the Chitosan based drug delivery and biomedical applications. Eur. Polym. J..

[99] Wang Y., Zhang R., Qin W., Dai J., Zhang Q., Lee K., Liu Y. (2020). Physicochemical properties of gelatin films containing tea polyphenol-loaded chitosan nanoparticles generated by electrospray. Mater. Des..

[100] Perera U.M.S.P., Rajapakse N. (2014). Chitosan nanoparticles: Preparation, characterization, and applications. Seafood Processing By-Products.

[101] Gao Y., Ma Q., Cao J., Wang Y., Yang X., Xu Q., Liang Q., Sun Y. (2021). Recent advances in microfluidic-aided chitosan-based multifunctional materials for biomedical applications. Int. J. Pharm..

[102] Ma Q., Cao J., Gao Y., Han S., Liang Y., Zhang T., Wang X., Sun Y. (2020). Microfluidic-mediated nano-drug delivery systems: From fundamentals to fabrication for advanced therapeutic applications. Nanoscale.

[103] Zhu C.Z., Yao R.Y., Chen Y.J., Feng M.R., Ma S., Zhang C.C. (2018). Self-assembly of fluorinated gradient copolymer in three-dimensional co-flow focusing microfluidic. J. Colloid Interface Sci..

[104] Collins D.J., Neild A., de Mello A., Liu A.Q., Ai Y. (2015). The Poisson distribution and beyond: Methods for microfluidic droplet production and single cell encapsulation. Lab. Chip.

[105] Gomez-Mascaraque L.G., Sanchez G., Lopez-Rubio A. (2016). Impact of molecular weight on the formation of electrosprayed chitosan microcapsules as delivery vehicles for bioactive compounds. Carbohydr. Polym..

[106] Lari A.S., Zahedi P., Ghourchian H., Khatibi A. (2021). Microfluidic-based synthesized carboxymethyl chitosan nanoparticles containing metformin for diabetes therapy: In vitro and in vivo assessments. Carbohydr. Polym..

[107] Farahani M., Moradikhah F., Shabani I., Soflou R.K., Seyedjafari E. (2021). Microfluidic fabrication of berberine-loaded nanoparticles for cancer treatment applications. J. Drug Deliv. Sci. Technol..

[108] Chisty A.H., Masud R.A., Hasan M.M., Khan M.N., Mallik A.K., Rahman M.M. (2020). Chapter 3—PEGylated chitin and chitosan derivatives. Handbook of Chitin and Chitosan.

[109] Malhotra M., Tomaro-Duchesneau C., Saha S., Prakash S. (2013). Systemic siRNA Delivery via peptide-tagged polymeric nanoparticles, targeting PLK1 gene in a mouse xenograft model of colorectal Cancer. Int. J. Biomater..

[110] Casey S.L., Wilson L.D. (2015). Investigation of Chitosan-PVA composite films and their adsorption properties. J. Geosci. Environ. Prot..

[111] Menazea A.A., Ismail A.M., Awwad N.S., Ibrahium H.A. (2020). Physical characterization and antibacterial activity of PVA/Chitosan matrix doped by selenium nanoparticles prepared via one-pot laser ablation route. J. Mater. Res. Technol..

[112] Sohail R., Abbas S.R. (2020). Evaluation of amygdalin-loaded alginate-chitosan nanoparticles as biocompatible drug delivery carriers for anticancerous effificacy. Int. J. Biol. Macromol..

[113] Wang F., Li J., Tang X., Huang K., Chen L. (2020). Polyelectrolyte three layer nanoparticles of chitosan/dextran sulfate/chitosan for dual drug delivery. Colloid Surf. B Biointerfaces.

[114] Hu Q., Hu S., Fleming E., Lee J.Y., Luo Y. (2020). Chitosan-Caseinate-Dextran ternary complex nanoparticles for potential oral delivery of astaxanthin with signifificantly improved bioactivity. Int. J. Biol. Macromol..

[115] Sabra R., Roberts C.J., Billa N. (2019). Courier properties of modified citrus pectinate-chitosan nanoparticles in colon delivery of curcumin. Colloid Interface Sci. Commun..

[116] Lopes M., Shrestha N., Correia A., Shahbazi M.A., Sarmento B., Hirvonen J., Veiga F., Seiça R., Ribeiro A., Santos H.A. (2016). Dual chitosan/albumin-coated alginate/dextran sulfate nanoparticles for enhanced oral delivery of insulin. J. Control. Release.

[117] Anirudhan T.S., Parvathy J. (2018). Novel Thiolated Chitosan-Polyethyleneglycol blend/Montmorillonite composite formulations for the oral delivery of insulin. Bioact. Carbohydr. Diet. Fibre.

[118] Li J., Tian S., Tao Q., Zhao Y., Gui R., Yang F., Zang L., Chen Y., Ping Q., Hou D. (2018). Montmorillonite/Chitosan nanoparticles as a novel controlled-release topical ophthalmic delivery system for the treatment of glaucoma. Int. J. Nanomed..

[119] Luo C., Yang Q., Lin X., Qi C., Li G. (2019). Preparation and drug release property of tanshinone IIA loaded chitosan montmorillonite microspheres. Int. J. Biol. Macromol..

[120] Hou Y.T., Wu K.C.E., Lee C.Y. (2020). Development of glycyrrhizin-conjugated, chitosan-coated, lysine-embedded mesoporous silica nanoparticles for hepatocyte-targeted liver tissue regeneration. Materialia.

[121] Chen C., Yao W., Sun W., Guo T., Lv H., Wang X., Ying H., Wang Y., Wang P. (2019). A self-targeting and controllable drug delivery system constituting mesoporous silica nanoparticles fabricated with a multi-stimuli responsive chitosan-based thin film layer. Int. J. Biol. Macromol..

[122] Qiu Q., Quan Z., Zhang H., Qin X., Wang R., Yu J. (2020). pH-triggered sustained drug release of multilayer encapsulation system with hollow mesoporous silica nanoparticles/chitosan/polyacrylic acid. Mater. Lett..

[123] Liao T., Liu C., Ren J., Chen H., Kuang Y., Jiang B., Chen J., Sun Z., Li C. (2021). A chitosan/mesoporous silica nanoparticle-based anticancer drug delivery system with a “tumor-triggered targeting” property. Int. J. Biol. Macromol..

[124] Piosik E., Klimczak P., Ziegler-Borowska M., Chełminiak-Dudkiewicz A. (2020). A detailed investigation on interactions between magnetite nanoparticles functionalized with aminated chitosan and a cell model membrane. Mater. Sci. Eng. C.

[125] Salmanian G., Hassanzadeh-Tabrizi S.A., Koupaei N. (2021). Magnetic chitosan nanocomposites for simultaneous hyperthermia and drug delivery applications: A review. Int. J. Biol. Macromol..

[126] Araujo H.C., Gomes da Silva A.C., Paião L.I., Magario M.K.W., Frasnelli F.C.T., Oliveira S.H.P., Pessan J.P., Monteiro D.R. (2020). Antimicrobial, antibiofilm and cytotoxic effects of a colloidal nanocarrier composed by chitosan-coated iron oxide nanoparticles loaded with chlorhexidine. J. Dent..

[127] Khmara I., Molcan M., Antosova A., Bednarikova Z., Zavisova V., Kubovcikova K., Jurikova A., Girman V., Baranovicova E., Koneracka M. (2020). Bioactive properties of chitosan stabilized magnetic nanoparticles—Focus on hyperthermic and anti-amyloid activities. J. Magn. Magn. Mater..

[128] Bandeira A.C., de Oliveira Matos A., Evangelista B.S., da Silva S.M., Nagib P.R.A., de Moraes Crespo A., Amaral A.C. (2019). Is it possible to track intracellular chitosan nanoparticles using magnetic nanoparticles as contrast agent?. Bioorg. Med. Chem..

[129] Anirudhan T.S., Sekhar V.C., Athira V.S. (2020). Graphene oxide based functionalized chitosan polyelectrolyte nanocomposite for targeted and pH responsive drug delivery. Int. J. Biol. Macromol..

[130] Rebekah A., Sivaselvam S., Viswanathan C., Prabhu D., Gautam R., Ponpandian N. (2021). Magnetic nanoparticle-decorated graphene oxide-chitosan composite as an efficient nanocarrier for protein delivery. Colloid Surf. A Physicochem. Eng. Asp..

[131] Baktash M.S., Zarrabi A., Avazverdi E., Reis N.M. (2021). Development and optimization of a new hybrid chitosan-grafted graphene oxide/magnetic nanoparticle system for theranostic applications. J. Mol. Liq..

[132] Khademi Z., Lavaee P., Ramezani M., Alibolandi M., Abnous K., Taghdisi S.M. (2020). Co-Delivery of doxorubicin and aptamer against Forkhead box M1 using chitosan-gold nanoparticles coated with nucleolin aptamer for synergistic treatment of cancer cells. Carbohydr. Polym..

[133] Salem D.S., Hegazy S.F., Obayya S.S.A. (2021). Nanogold-loaded chitosan nanocomposites for pH/light-responsive drug release and synergistic chemo-photothermal cancer therapy. Colloid Interface Sci. Commun..

[134] Saravanakumar K., Mariadoss A.V.A., Sathiyaseelan A., Wang M.H. (2020). Synthesis and characterization of nano-chitosan capped gold nanoparticles with multifunctional bioactive properties. Int. J. Biol. Macromol..

[135] López-Pérez G., Prado-Gotor R., Fuentes-Rojas J.A., Martin-Valero M.J. (2020). Understanding gold nanoparticles interactions with chitosan: Crosslinking agents as novel strategy for direct covalent immobilization of biomolecules on metallic surfaces. J. Mol. Liq..

[136] Wu H., Zhang J. (2018). Chitosan-based zinc oxide nanoparticle for enhanced anticancer effect in cervical cancer: A physicochemical and biological perspective. Saudi Pharm. J..

[137] Singh T.A., Das J., Sil P.C. (2020). Zinc oxide nanoparticles: A comprehensive review on its synthesis, anticancer and drug delivery applications as well as health risks. Adv. Colloid Interface Sci..

[138] George D., Maheswari P.U., Begum K.M.M.S. (2020). Cysteine conjugated chitosan based green nanohybrid hydrogel embedded with zinc oxide nanoparticles towards enhanced therapeutic potential of naringenin. React. Funct. Polym..

[139] Ghaffari S.B., Sarrafzadeh M.H., Salami M., Khorramizadeh M.R. (2020). A pH-sensitive delivery system based on N-succinyl chitosan-ZnO nanoparticles for improving antibacterial and anticancer activities of curcumin. Int. J. Biol. Macromol..

[140] Kandra R., Bajpai S. (2020). Synthesis, mechanical properties of fluorescent carbon dots loaded nanocomposites chitosan film for wound healing and drug delivery. Arab. J. Chem..

[141] Lin C., Sun K., Zhang C., Tan T., Xu M., Liu Y., Xu C., Wang Y., Li L., Whittaker A. (2020). Carbon dots embedded metal organic framework @ chitosan core-shell nanoparticles for vitro dual mode imaging and pH-responsive drug delivery. Microporous Mesoporous Mater..

[142] Sheng Y., Dai W., Gao J., Li H., Tan W., Wang J., Deng L., Kong Y. (2020). pH-sensitive drug delivery based on chitosan wrapped graphene quantum dots with enhanced flfluorescent stability. Mater. Sci. Eng. C.

[143] Dong X., Wei C., Liang J., Liu T., Kong D., Lv F. (2017). Thermosensitive hydrogel loaded with chitosan-carbon nanotubes for near infrared light triggered drug delivery. Colloid Surf. B Biointerfaces.

[144] Singh R.P., Sonali G.S., Singh S., Bharti S., Pandey B.L., Koch B., Muthu M.S. (2017). Chitosan-Folate decorated carbon nanotubes for site specific lung cancer delivery. Mater. Sci. Eng. C.

[145] Ghaffar I., Imran M., Perveen S., Kanwal T., Saifullah S., Bertino M.F., Ehrhardt C.J., Yadavalli V.K., Shah M.R. (2019). Synthesis of chitosan coated metal organic frameworks (MOFs) for increasing vancomycin bactericidal potentials against resistant S. *aureus* strain. Mater. Sci. Eng. C.

[146] Li L., Han S., Zhao S., Li X., Liuab B., Liu Y. (2020). Chitosan modified metal–organic frameworks as a promising carrier for oral drug delivery. RSC Adv..

[147] Xu T., Zhang J., Chi H., Cao F. (2016). Multifunctional properties of organic-inorganic hybrid nanocomposites based on chitosan derivatives and layered double hydroxides for ocular drug delivery. Acta Biomaterialia.

[148] Wang Y., Zhou L., Fang L., Cao F. (2020). Multifunctional carboxymethyl chitosan derivatives-layered double hydroxide hybrid nanocomposites for effifficient drug delivery to the posterior segment of the eye. Acta Biomaterialia.

[149] Elanchezhiyan S.S., Meenakshi S. (2017). Synthesis and characterization of chitosan/Mg-Al layered double hydroxide composite for the removal of oil particles from oil-in-water emulsion. Int. J. Biol. Macromol..

[150] Sabourian P., Tavakolian M., Yazdani H., Frounchi M., van de Ven T.G.M., Maysinger D., Kakkar A. (2020). Stimuli-responsive chitosan as an advantageous platform for efficient delivery of bioactive agents. J. Control. Release.

[151] Constantin M., Bucatariu S.M., Doroftei F., Fundueanu G. (2017). Smart composite materials based on chitosan microspheres embedded in thermosensitive hydrogel for controlled delivery of drugs. Carbohydr. Polym..

[152] Liu H., Liu J., Xie X., Li X. (2020). Development of photo-magnetic drug delivery system by facile-designed dual stimuli-responsive modifed biopolymeric chitosan capped nano-vesicle to improve efficiency in the anesthetic effect and its biological investigations. J. Photochem. Photobiol. B Biol..

[153] Daund V., Chalke S., Sherje A.P., Kale P.P. (2021). ROS responsive mesoporous silica nanoparticles for smart drug delivery: A review. J. Drug Deliv. Sci. Technol..

[154] Jiao J., Li X., Zhang S., Liu J., Di D., Zhang Y., Zhao Q., Wang S. (2016). Redox and pH dual-responsive PEG and chitosan-conjugated hollow mesoporous silica for controlled drug release. Mater. Sci. Eng. C.

[155] Lin J.T., Liu Z.K., Zhu Q.L., Rong X.H. (2017). Liang, C.L.; Wang, J.; Ma, D.; Sun, J.; Wan, G.H. Redox-Responsive nanocarriers for drug and gene co-delivery based on chitosan derivatives modifified mesoporous silica nanoparticles. Colloid Surf. B Biointerfaces.

[156] Rastegari B., Karbalaei-Heidari H.R., Zeinali S., Sheardown H. (2017). The enzyme-sensitive release of prodigiosin grafted β-cyclodextrin and chitosan magnetic nanoparticles as an anticancer drug delivery system: Synthesis, characterization and cytotoxicity studies. Colloid Surf. Biointerfaces.

[157] Baghbani F., Chegeni M., Moztarzadeh F., Hadian-Ghazvini S., Raz M. (2017). Novel ultrasound-responsive chitosan/perfluorohexane nanodroplets for image-guided smart delivery of an anticancer agent: Curcumin. Mat. Sci. Eng. C.

[158] Mathew S.A., Prakash P.A., Jaabir M.S.M., Dhanavel S., Manikandan R., Stephen A. (2021). Dopamine-Conjugated CuS/chitosan nanocomposite for targeted photothermal drug delivery: In vitro cytotoxicity study to establish bio-compatibility. J. Drug Deliv. Sci. Technol.

[159] Bhatta A., Krishnamoorthy G., Marimuthu N., Dihingia A., Manna P., Biswal H.T., Das M., Krishnamoorthy G. (2019). Chlorin e6 decorated doxorubicin encapsulated chitosan nanoparticles for photo-controlled cancer drug delivery. Int. J. Biol. Macromol..

[160] Sudhakar S., Chandran S.V., Selvamurugan N., Nazeer R.A. (2020). Biodistribution and pharmacokinetics of thiolated chitosan nanoparticles for oral delivery of insulin in vivo. Int. J. Biol. Macromol..

[161] Mumuni M.A., Kenechukwu F.C., Ofokansi K.C., Attama A.A., Díaz Díaz D. (2020). Insulin-loaded mucoadhesive nanoparticles based on mucin-chitosan complexes for oral delivery and diabetes treatment. Carbohydr. Polym..

[162] Tsai L.C., Chen C.H., Lin C.W., Ho Y.C., Mi F.L. (2019). Development of multifunctional nanoparticles self-assembled from trimethyl chitosan and fucoidan for enhanced oral delivery of insulin. Int. J. Biol. Macromol..

[163] Wong C.Y., Al-Salami H., Dass C.R. (2020). Formulation and characterisation of insulin-loaded chitosan nanoparticles capable of inducing glucose uptake in skeletal muscle cells in vitro. J. Drug Deliv. Sci. Technol..

[164] Abdel-Moneim A., El-Shahawy A., Yousef A.I., Abd El-Twab S.M., Elden Z.E., Taha M. (2020). Novel polydatin-loaded chitosan nanoparticles for safe and efficient type 2 diabetes therapy: In silico, in vitro and in vivo approaches. Int. J. Biol. Macromol..

[165] Coutinho A.J., Costa Lima S.A., Afonso C.M.M., Reis S. (2020). Mucoadhesive and pH responsive fucoidan-chitosan nanoparticles for the oral delivery of methotrexate. Int. J. Biol. Macromol..

[166] Xavier F.H., Gueutin C., Chacun H., Vauthier C., Egito E.S.T. (2019). Mucoadhesive paclitaxel-loaded chitosan-poly (isobutyl cyanoacrylate) core-shell nanocapsules containing copaiba oil designed for oral drug delivery. J. Drug Deliv. Sci. Technol..

[167] Du X., Yin S. (2020). Xu, L.; Ma, J.; Yu, H.; Wang, G.; Li, J. Polylysine and cysteine functionalized chitosan nanoparticle as an efficient platform for oral delivery of paclitaxel. Carbohydr. Polym..

[168] Tran P.H.L., Wang T., Yang C., Tran T.T.D., Duan W. (2020). Development of conjugate-by-conjugate structured nanoparticles for oral delivery of docetaxel. Mater. Sci. Eng. C.

[169] Sharma M., Sharma R., Jain D.K., Saraf A. (2019). Enhancement of oral bioavailability of poorly water soluble carvedilol by chitosan nanoparticles: Optimization and pharmacokinetic study. Int. J. Biol. Macromol..

[170] Pauluk D., Padilha A.K., Khalil N.M., Mainardes R.M. (2019). Chitosan-Coated zein nanoparticles for oral delivery of resveratrol: Formation, characterization, stability, mucoadhesive properties and antioxidant. Food Hydrocoll..

[171] Lin C., Kuo T.C., Lin J.C., Ho Y.C., Mi F.L. (2020). Delivery of polysaccharides from Ophiopogon japonicus (OJPs) using OJPs/chitosan/whey protein co-assembled nanoparticles to treat defective intestinal epithelial tight junction barrier. Int. J. Biol. Macromol..

[172] Ling K., Wu H., Neish A.S., Champion J.A. (2019). Alginate/Chitosan microparticles for gastric passage and intestinal release of therapeutic protein nanoparticles. J. Control. Release.

[173] Muye H., Chen Z., Huibing H., Yu J., Yanzuo C., Kaiyan L., Feng G. (2019). Cyclodextrin/chitosan nanoparticles for oral ovalbumin delivery: Preparation, characterization and intestinal mucosal immunity in mice. Asian J. Pharm. Sci..

[174] Cole H., Bryan D., Lancaster L., Mawas F., Vllasaliu D. (2018). Chitosan nanoparticle antigen uptake in epithelial monolayers can predict T mucosal but not systemic in vivo immune response by oral delivery. Carbohydr. Polym..

[175] Saraf S., Jain S., Sahoo R.N., Mallick S. (2020). Lipopolysaccharide derived alginate coated Hepatitis B antigen loaded chitosan nanoparticles for oral mucosal immunization. Int. J. Biol. Macromol..

[176] Yu X., Wen T., Cao P., Shan L., Li L. (2019). Alginate-chitosan coated layered double hydroxide nanocomposites for enhanced oral vaccine delivery. J. Colloid Interface Sci..

[177] Dong W., Wang X., Liu C., Zhang X., Zhang X., Chen X., Kou Y., Mao S. (2018). Chitosan based polymer-lipid hybrid nanoparticles for oral delivery of enoxaparin. Int. J. Pharm..

[178] Murthy A., Ravi P.R., Kathuria H., Vats R. (2020). Self-Assembled lecithin-chitosan nanoparticles improve the oral bioavailability and alter the pharmacokinetics of raloxifene. Int. J. Pharm..

[179] Yu A., Shi H., Liu H., Bao Z., Dai M., Lin D., Lin D., Xu X., Li X., Wang Y. (2020). Mucoadhesive dexamethasone-glycol chitosan nanoparticles for ophthalmic drug delivery. Int. J. Pharm..

[180] Khalil M., Hashmi U., Riaz R., Abbas S.R. (2020). Chitosan coated liposomes (CCL) containing triamcinolone acetonide for sustained delivery: A potential topical treatment for posterior segment Diseases. Int. J. Biol. Macromol..

[181] Ameeduzzafar, Imam S.S., Bukhari S.N.A., Ahmad J., Ali A. (2018). Formulation and optimization of levoflfloxacin loaded chitosan nanoparticle for ocular delivery: In-vitro characterization, ocular tolerance and antibacterial activity. Int. J. Biol. Macromol..

[182] Cheng Y.H., Ko Y.C., Chang Y.F., Huang S.H., Liu C.J. (2019). Thermosensitive chitosan-gelatin-based hydrogel containing curcuminloaded nanoparticles and latanoprost as a dual-drug delivery system for glaucoma treatment. Exp. Eye Res..

[183] Abruzzo A., Cerchiara T., Bigucci F., Zuccheri G., Cavallari C., Saladini B., Luppi B. (2019). Cromolyn-Crosslinked chitosan nanoparticles for the treatment of allergic Rhinitis. Eur. J. Pharm. Sci..

[184] Sun M., Yu X., Wang T., Bi S., Liu Y., Chen X. (2019). Nasal adaptive chitosan-based nano-vehicles for anti-allergic drug delivery. Int. J. Biol. Macromol..

[185] Dumkliang E., Pamornpathomkul B., Patrojanasophon P., Ngawhirunpat T., Rojanarata T., Yoksan S., Opanasopit P. (2021). Feasibility of chitosan-based nanoparticles approach for intranasal immunisation of live attenuated Japanese encephalitis vaccine. Int. J. Biol. Macromol..

[186] Piazzini V., Landucci E., D’Ambrosio M., Fasiolo L.T., Cinci L., Colombo G., Pellegrini-Giampietro D.E., Bilia A.R., Luceri C., Bergonzi M.C. (2019). Chitosan coated human serum albumin nanoparticles: A promising strategy for nose-to-brain drug delivery. Int. J. Biol. Macromol..

[187] Chatzitaki A.T., Karavasili S.J.C., Andreadis D., Fatouros D.G., Borges O. (2020). Chitosan-Coated PLGA nanoparticles for the nasal delivery of ropinirole hydrochloride: In vitro and ex vivo evaluation of efficacy and safety. Int. J. Pharm..

[188] Cassano R., Trapani A., Di Gioia M.L., Mandracchia D., Pellitteri R., Tripodo G., Trombino S., Di Gioia S., Conese M. (2020). Synthesis and characterization of novel chitosan-dopamine or chitosantyrosine conjugates for potential nose-to-brain delivery. Int. J. Pharm..

[189] Sava V., Fihurka O., Khvorova A., Sanchez-Ramos J. (2020). Enriched chitosan nanoparticles loaded with siRNA are effective in lowering Huntington’s disease gene expression following intranasal administration. Nanomed. Nanotechnol. Biol. Med..

[190] Khezri F.A.N.Z., Lakshmi C.S.R., Bukka R., Nidhi M., Nargund S.L. (2020). Pharmacokinetic study and brain tissue analysis of Zolmitriptan loaded chitosan nanoparticles in rats by LC-MS method. Int. J. Biol. Macromol..

[191] Bhattamisra S.K., Shak A.T., Xi L.W., Safian N.H., Choudhury H., Lim W.M., Shahzad N., Alhakamy N.A., Anwer K., Radhakrishnan A.K. (2020). Nose to brain delivery of rotigotine loaded chitosan nanoparticles in human SH-SY5Y neuroblastoma cells and animal model of Parkinson’s disease. Int. J. Pharm..

[192] Wang H., Holgate J., Bartlett S., Islam N. (2020). Assessment of nicotine release from nicotine-loaded chitosan nanoparticles dry powder inhaler formulations via locomotor activity of C57BL/6 mice. Eur. J. Pharm. Biopharm..

[193] Ahmad N., Ahmad R., Alrasheed R.A., Almatar H.M.A., Al-Ramadan A.S., Buheazah T.M., AlHomoud H.S., Al-Nasif H.A., Alam A. (2020). A Chitosan-PLGA based catechin hydrate nanoparticles used in targeting of lungs and cancer treatment. Saudi J. Biol. Sci..

[194] Vieira A.C.C., Chaves L.L., Pinheiro M., Costa Lima S., Rolim Neto P.J., Ferreira D., Sarmento B., Reis S. (2021). Lipid nanoparticles coated with chitosan using a one-step association method to target rifampicin to alveolar macrophages. Carbohydr. Polym..

[195] Pardeshi C.V., Agnihotri V.V., Patil K.Y., Pardeshi S.R., Surana S.J. (2020). Mannose-Anchored *N*,*N*,*N*-trimethyl chitosan nanoparticles for pulmonary administration of etofylline. Int. J. Biol. Macromol..

[196] Dhayanandamoorthy Y., Antoniraj M.G., Kandregula C.A.B., Kandasamy R. (2020). Aerosolized hyaluronic acid decorated, ferulic acid loaded chitosan nanoparticle: A promising asthma control strategy. Int. J. Pharm..

[197] Rahbarian M., Mortazavian E., Dorkoosh F.A., Tehrani M.R. (2018). Preparation, evaluation and optimization of nanoparticles composed of thiolated triethyl chitosan: A potential approach for buccal delivery of Insulin. J. Drug Deliv. Sci. Technol..

[198] Stie M.B., Gätke J.R., Wan F., Chronakis J.S., Jacobsen J., Nielsen H.M. (2020). Swelling of mucoadhesive electrospun chitosan/polyethylene oxide nanofibers facilitates adhesion to the sublingual mucosa. Carbohydr. Polym..

[199] Martin V., Ribeiro I.A.C., Alves M.M., Gonçalves L., Almeida A.J., Grenho L., Fernandes M.H., Santos C.F., Gomes P.S., Bettencourt A.F. (2019). Understanding intracellular trafficking and anti-inflammatory effects of minocycline chitosan-nanoparticles in human gingival fibroblasts for periodontal disease treatment. Int. J. Pharm..

[200] Dos Santos D.M., Chagas P.A.M., Leite I.S., Inada N.M., de Annunzio S.R., Fontana C.R., Campana-Filho S.P., Correa D.S. (2020). Core-sheath nanostructured chitosan-based nonwovens as a potential drug delivery system for periodontitis treatment. Int. J. Biol. Macromol..

[201] Abd-Allah H., Abdel-Aziz R.T.A., Nasr M. (2020). Chitosan nanoparticles making their way to clinical practice: A feasibility study on their topical use for acne treatment. Int. J. Biol. Macromol..

[202] Ushirobira C.Y., Afiune L.A.F., Pereira M.N., Cunha-Filho M., Gelfuso G.M., Gratieri T. (2020). Dutasteride nanocapsules for hair follicle targeting: Effect of chitosan-coating and physical stimulus. Int. J. Biol. Macromol..

[203] Takeuchi I., Suzuki T., Makino K. (2020). Iontophoretic transdermal delivery using chitosan-coated PLGA nanoparticles for transcutaneous immunization. Colloid Surf. A Physicochem. Eng. Asp..

[204] Morad H., Jahanshahi M., Akbari J., Saeedi M., Gill P., Enayatifard R. (2021). Novel topical and transdermal delivery of colchicine with chitosan based biocomposite nanofiberous system; formulation, optimization, characterization, ex vivo skin deposition/permeation, and anti-melanoma evaluation. Mater. Chem. Phys..

[205] Nawaz A., Wong T.W. (2017). Microwave as skin permeation enhancer for transdermal drug delivery of chitosan-5-fluorouracil nanoparticles. Carbohydr. Polym..

[206] Fahimirad S., Abtahi H., Satei P., Ghaznavi-Rad E., Moslehi M., Ganji A. (2021). Wound healing performance of PCL/Chitosan based electrospun nanofiber electrosprayed with curcumin loaded chitosan nanoparticles. Carbohydr. Polym..

[207] Shafique M., Sohail M., Minhas M.U., Khaliq T., Kousar M., Khan S., Hussain Z., Mahmood A., Abbasi M., Aziz H.C. (2021). Bio-Functional hydrogel membranes loaded with chitosan nanoparticles for accelerated wound healing. Int. J. Biol. Macromol..

[208] Manne A.A., Arigela B., Giduturi A.K., Komaravolu R.K., Mangamuri U., Poda S. (2021). *Pterocarpus marsupium* Roxburgh heartwood extract/chitosan nanoparticles loaded hydrogel as an innovative wound healing agent in the diabetic rat model. Mater. Today Commun..

[209] Amaral A.C., Saavedra P.H.V., Souza A.C.O., de Melo M.T., Tedesco A.C., Morais P.C., Felipe M.S.S., Bocca A.L. (2019). Miconazole loaded chitosan-based nanoparticles for local treatment of vulvovaginal candidiasis fungal infections. Colloids Surf. B Biointerfaces.

[210] Marciello M., Rossi S., Caramella C., Remunán-López C. (2017). Freeze-Dried cylinders carrying chitosan nanoparticles for vaginal peptide delivery. Carbohydr. Polym..

[211] Arumugam G., Rajendran R. (2020). Callophycin A loaded chitosan and spicules based nanocomposites as an alternative strategy to overcome vaginal candidiasis. Int. J. Biol. Macromol..

[212] Abdel Allah N.H., Gaber Y., Rashed M.E., Azmy A.F., Abou-Taleb H.A., Abdel Ghani S. (2020). Alginate-Coated chitosan nanoparticles act as effective adjuvant for hepatitis A vaccine in mice. Int. J. Biol. Macromol..

[213] Rebbouh F., Martin-Eauclaire M.F., Laraba-Djebari F. (2020). Chitosan nanoparticles as a delivery platform for neurotoxin II from *Androctonus australis* hector scorpion venom: Assessment of toxicity and immunogenicity. Acta Tropica.

[214] Abdelhamid H.N., Dowaidar M., Langel U. (2020). Carbonized chitosan encapsulated hierarchical porous zeolitic imidazolate frameworks nanoparticles for gene delivery. Microporous Mesoporous Mater..

[215] Babii O., Wang Z., Liu G., Martinez E.C., van den Hurk S.D.L., Chen L. (2020). Low molecular weight chitosan nanoparticles for CpG oligodeoxynucleotides delivery: Impact of molecular weight, degree of deacetylation, and mannosylation on intracellular uptake and cytokine induction. Int. J. Biol. Macromol..

[216] Rahmani S., Hakimi S., Esmaeily A., Samadi F.Y., Mortazavian E., Nazari M., Mohammadi Z., Tehrani N.R., Tehrani M.R. (2019). Novel chitosan based nanoparticles as gene delivery systems to cancerous and noncancerous cells. Int. J. Pharm..

[217] Mobarakeh V.I., Modarressi M.H., Rahimi P., Bolhassani A., Arefian E., Atyabi F., Vahabpour R. (2019). Optimization of chitosan nanoparticles as an anti-HIV siRNA delivery vehicle. Int. J. Biol. Macromol..

[218] Baghaei M., Tekie F.S.M., Khoshayand M.R., Varshochian R., Hajiramezanali M., Kachousangi M.J., Dinarvand R., Atyabiet F. (2021). Optimization of chitosan-based polyelectrolyte nanoparticles for gene delivery, using design of experiment: In vitro and in vivo study. Mater. Sci. Eng. C.

[219] Yan L., Gao S., Shui S., Liu S., Qu H., Liu C., Zheng L. (2020). Small interfering RNA-loaded chitosan hydrochloride/carboxymethyl chitosan nanoparticles for ultrasound-triggered release to hamper colorectal cancer growth in vitro. Int. J. Biol. Macromol..

[220] Tang Y., Liu Y., Xie Y., Chen J., Dou Y. (2020). Apoptosis of A549 cells by small interfering RNA targeting survivin delivery using poly-β-amino ester/guanidinylated O-carboxymethyl chitosan nanoparticles. Asian J. Pharm. Sci..

[221] Hajizadeh F., Ardebili S.M., Moornani M.B., Masjedi A., Atyabi F., Kiani M., Namdar A., Karpisheh V., Izadi S., Baradaran B. (2020). Silencing of HIF-1α/CD73 axis by siRNA-loaded TAT-chitosan-spion nanoparticles robustly blocks cancer cell progression. Eur. J. Pharmacol..

[222] Salimifard S., Kiani F.K., Eshaghi F.S., Izadi S., Shahdadnejad K., Masjedi A., Heydari M., Ahmadi A., Hojjat-Farsangi M., Hassannia H. (2020). Codelivery of BV6 and anti-IL6 siRNA by hyaluronate-conjugated PEGchitosan-lactate nanoparticles inhibits tumor progression. Life Sci..

[223] Nikkhoo A., Rostami N., Farhadi S., Esmaily M., Ardebili S.M., Atyabi F., Baghaei M., Haghnavaz N., Yousefi M., Aliparasti M.R. (2020). Codelivery of STAT3 siRNA and BV6 by carboxymethyl dextran trimethyl chitosan nanoparticles suppresses cancer cell progression. Int. J. Pharm..

[224] Masjedi A., Ahmadi A., Atyabi F., Farhadi S., Irandoust M., Khazaei-Poul Y., Chaleshtari M.G., Fathabad M.E., Baghaei M., Haghnavaz N. (2020). Silencing of IL-6 and STAT3 by siRNA loaded hyaluronate-*N*,*N*,*N*-trimethyl chitosan nanoparticles potently reduces cancer cell progression. Int. J. Biol. Macromol..

[225] Yan T., Zhu S., Hui W., He J., Liu Z., Cheng J. (2020). Chitosan based pH-responsive polymeric prodrug vector for enhanced tumor targeted co-delivery of doxorubicin and siRNA. Carbohydr. Polym..

[226] Xue Y., Wang N., Zeng Z., Huang J., Xiang Z., Guan J.Q. (2020). Neuroprotective effect of chitosan nanoparticle gene delivery system grafted with acteoside (ACT) in Parkinson’s disease models. J. Mater. Sci. Technol..

[227] Lang X., Wang T., Sun M., Chen X., Liu Y. (2020). Advances and applications of chitosan-based nanomaterials as oral delivery carriers. Int. J. Biol. Macromol..

[228] Alhajj N., Zakaria Z., Naharudin I., Ahsan F., Li W., Wong T.W. (2020). Critical physicochemical attributes of chitosan nanoparticles admixed lactose-PEG 3000 microparticles in pulmonary inhalation. Asian J. Pharm. Sci..

[229] Macedo A.S., Castro P.M., Roque L., Thome N.G., Reis C.P., Pintado M.E., Fonte P. (2020). Novel and revisited approaches in nanoparticle systems for buccal drug delivery. J. Control. Release.

[230] Matos B.N., Pereira M.N., Bravo M.O., Cunha-Filho M., Saldanha-Araújo F., Gratieri T., Gelfuso G. (2020). Chitosan nanoparticles loading oxaliplatin as a mucoadhesive topical treatment of oral tumors: Iontophoresis further enhances drug delivery ex vivo. Int. J. Biol. Macromol..

[231] Pornpitchanarong C., Rojanarata T., Opanasopit P., Ngawhirunpat T., Patrojanasophon P. (2020). Catechol-modified chitosan/hyaluronic acid nanoparticles as a new avenue for local delivery of doxorubicin to oral cancer cells. Colloids Surf. B Biointerfaces.

[232] Sah A.K., Dewangan M., Suresh P.K. (2019). Potential of chitosan-based carrier for periodontal drug delivery. Colloids Surf. B Biointerfaces.

[233] Nair S.S. (2019). Chitosan-based transdermal drug delivery systems to overcome skin barrier functions. J. Drug Deliv. Ther..

[234] Park J., Ramanathan R., Pham L., Kim B.S., Woodrow A. (2017). Chitosan enhances nanoparticle delivery from the reproductive tract to target draining lymphoid organs. Nanomed. Nanotechnol. Biol. Med..

